# Identification of Loci and Pathways Associated with Heifer Conception Rate in U.S. Holsteins

**DOI:** 10.3390/genes11070767

**Published:** 2020-07-08

**Authors:** Justine M. Galliou, Jennifer N. Kiser, Kayleen F. Oliver, Christopher M. Seabury, Joao G. N. Moraes, Gregory W. Burns, Thomas E. Spencer, Joseph Dalton, Holly L. Neibergs

**Affiliations:** 1 Department of Animal Sciences and Center for Reproductive Biology, Washington State University, Pullman, WA 99164, USA; justine.galliou@wsu.edu (J.M.G.); jennifer.kiser@wsu.edu (J.N.K.); kayleen.oliver@wsu.edu (K.F.O.); 2 Department of Veterinary Pathobiology, College of Veterinary Medicine, Texas A&M University, College Station, TX 77843, USA; CSeabury@cvm.tamu.edu; 3 Division of Animal Sciences, University of Missouri, Columbia, MO 65211, USA; jnmpt6@missouri.edu (J.G.N.M.); gbvd6@mail.missouri.edu (G.W.B.); spencerte@missouri.edu (T.E.S.); 4 Department of Animal and Veterinary Sciences, University of Idaho, Caldwell, ID 83844, USA; jdalton@uidaho.edu

**Keywords:** dairy heifer, genomic selection, loci, conception rate

## Abstract

Heifer conception rate (HCR) is defined as the percentage of inseminated heifers that become pregnant at each service. The genome-wide association analyses in this study focused on identifying the loci associated with Holstein heifer (*n* = 2013) conception rate at first service (HCR1) and the number of times bred (TBRD) to achieve a pregnancy. There were 348 unique loci associated (*p* < 5 × 10^−8^) with HCR1 and 615 unique loci associated (*p* < 5 × 10^−8^) with TBRD. The two phenotypes shared 302 loci, and 56 loci were validated in independent cattle populations. There were 52 transcription factor binding sites (TFBS) and 552 positional candidate genes identified in the HCR1- and TBRD-associated loci. The positional candidate genes and the TFBS associated with HCR1 and TBRD were used in the ingenuity pathway analysis (IPA). In the IPA, 11 pathways, 207 master regulators and 11 upstream regulators were associated (*p* < 1.23 × 10^−5^) with HCR1 and TBRD. The validated loci associated with both HCR1 and TBRD make good candidates for genomic selection and further investigations to elucidate the mechanisms associated with subfertility and infertility.

## 1. Introduction

The United States dairy industry has struggled with subfertility since the 1950s. Poor reproductive performance leads to decreased productivity and profitability due to elevated veterinary costs, additional inseminations and increased culling rates [1,2]. The economic burden of poor fertility in the dairy industry has reached over one billion dollars annually [3]. Due to the importance of fertility, dairy producers use several measures of reproductive performance for cows and heifers. Daughter pregnancy rate, defined as the percentage of a bull’s daughters who become pregnant in a 21-day period, is one of those measures [4]. Another reproductive performance measure is conception rate, defined as the percentage of inseminated cows and heifers that become pregnant at each service. Heifer conception rate (HCR) declined from approximately 60% in 1950 to 52% in the early 2000s [5]. This decline is likely due, in part, to selection for production traits without emphasis on selection for fertility traits [6,7,8]. Indeed, HCR has increased 6 percentage points since it was included in selection indices in 2014 [5]. However, the genomic structure underlying the physiological mechanisms responsible for subfertility is not well known.

Identifying loci associated with HCR may be performed through a genome-wide association analysis (GWAA). The GWAA may also provide insight into how variants may elicit physiological changes leading to pregnancy losses. Associated loci, once identified, may be used for genomic selection to improve HCR. Therefore, the objectives of this study were to identify (1) the loci associated with heifer conception rate in U.S. Holstein heifers; (2) the positional candidate genes and transcription factor binding sites (TFBS) within the associated loci; (3) the potential interactions and functions of the positional candidate genes and regulatory elements of the loci associated with HCR; and (4) the validated loci associated with HCR. The overarching goal of this research is to provide the foundation for the identification of causal mutations associated with HCR, and facilitate genomic selection for increased heifer fertility in the dairy industry.

## 2. Materials and Methods

### 2.1. Study Animals and Phenotypes

Two groups of heifers were combined for this study after receiving approval from Washington State University’s Institutional Animal Use and Care Committee (#4295). The first group consisted of 3060 heifers that were raised together at a heifer raising facility in Idaho, USA, as described by Kiser et al. 2019 (a) [9]. The second group consisted of 1071 two-year-old cows whose conception data was obtained as yearling heifers [10]. For both groups, heifers were only bred by artificial insemination (AI) once per estrous cycle. For the heifers that were evaluated as yearlings in the first group, the conception rates were 53% (1621/3060) to the first AI, 39% (567/1439) to the second AI, 30% (262/872) to the third AI, 57% (349/610) to the fourth AI, 3% (7/261) to the fifth AI and 8% (254/3060) were infertile (never conceived).

The second group of heifers were from six dairies in Washington, USA [10]. As these animals were sampled at their two-year-old year, only those animals that had conceived as a heifer remained in the dairy herds. This biased the sample toward animals that conceived early in the breeding season as 86% of the animals remaining in the herd conceived to the first or second AI. When all AI attempts were considered, the conception rates were 65% (692/1071) to the first AI, 60% (227/379) to the second AI, 59% (90/152) to the third AI, 71% (44/62) to the fourth AI, 67% (12/18) to the fifth AI, 83% (5/6) to the sixth AI and the remaining heifer conceived to the seventh AI.

From these two populations of heifers, genotypes were obtained from 1185 heifers that conceived to the first AI, 225 heifers that conceived to the second AI, 314 heifers that conceived to the third AI, 251 heifers that conceived to the fourth AI, 19 heifers that conceived to the fifth AI, 5 heifers that conceived to the sixth AI and 1 heifer that conceived to the seventh AI. One hundred and ninety infertile heifers were also genotyped.

Group 1 heifers were bred following observed estrus after reaching approximately 11–13 months of age, a minimum height of 129 cm at the withers and a minimum weight of 390 kg. Pregnancy was determined via rectal palpation 35 days after AI, and the DairyComp305 (Valley Agricultural Software, Tulare, CA, USA) health records were used to eliminate heifers that subsequently aborted their calf. One thousand different heifers were sampled in each month of December of 2012, May of 2013 and April 2015. A smaller number of samples (200 or less) were taken on June 2013, January 2014 and March 2015. Genotyping occurred as conception data became available, so the majority (70%) of heifers genotyped were from the December 2012 sampling. Heifers in the sub-fertile and infertile categories were less frequent and were more likely to be taken from samples collected over multiple years.

For Group 2 heifers, age at breeding and health records were assessed by DairyComp305 records, but physical characteristics, such as weight and height, were not available. The average age at first conception was 13.5 months. Pregnancy was also determined by rectal palpation after AI, and no heifers that subsequently aborted their calf were included in Group 2.

Two phenotypes were used for the analysis. The first phenotype was the heifer conception rate to the first AI (HCR1) and compared heifers that conceived to the first AI (*n* = 1185) to all heifers that conceived at a later AI (*n* = 815) or were infertile (*n* = 190). The second phenotype was the number of times bred (TBRD) required to achieve a pregnancy. In this phenotype, the number of times bred evaluated were conception to the first (*n* = 1185), second (*n* = 225), third (*n* = 314) or four or more (276) AI. Infertile cattle were not included in this phenotype.

### 2.2. DNA Extraction and Genotyping

Blood samples (~16 mL) were collected from the tail vein by venipuncture and were stored in EDTA tubes. DNA was extracted from white blood cell pellets using the Puregene DNA extraction kit (Gentra, Minneapolis, MN, USA) following the manufacturer’s instructions. A NanoDrop 1000 spectrophotometer (ThermoFisher Scientific, Wilmington, DE, USA) was used to quantify the DNA extracted, which was then genotyped at Neogen Laboratories (Lincoln, NE, USA). Heifers (*n* = 1544) representing the extreme ends of the phenotypic distribution, those conceiving to the first and fourth–seventh AI and infertile heifers, were genotyped with the Illumina (San Diego, CA, USA) BovineHD BeadChip. This chip contains 777,962 single nucleotide polymorphisms (SNPs) with an average distance of 3.43 kb between SNPs (http:/bovinegenome.org). Heifers that achieved pregnancy at the second or third AI were genotyped with the Neogen Bovine GGP50 BeadChip that contains 48,268 SNPs and shared over 44,000 SNPs with the Illumina BovineHD BeadChip (Neogen Laboratories). The GGP50 genotypes were imputed to include the BovineHD SNPs, based on a Holstein reference population of 5000 U.S. Holsteins using the Beagle imputation method [11]. Imputation was successful for 633,444 SNPs with an accuracy of 99% and used for the GWAA.

### 2.3. Quality Control

The heifers were filtered based on the percentage of genotypes that were successful and the SNPs were also filtered based on the percentage of genotypes called, minor allele frequency and failure of Hardy–Weinberg equilibrium. Out of the 5374 heifers used for the blood and data collection, a total of 2190 heifers were genotyped. The heifers that were not genotyped were due to health issues or because they fell into a large group of heifers that became pregnant after the first AI. Five hundred randomly selected heifers that became pregnant after the first AI were chosen for inclusion in the study to make the number of animals in each AI group more equal. After genotyping, heifers (*n* = 71) and SNPs (*n* = 7051) with a call rate < 0.90 were removed. Additional quality filters for heifers included samples that were duplicated (*n* = 13) and those that were bred to a bull or had phenotypic discrepancies (*n* = 93). Discrepancies between genotypic sex and phenotypic sex were tested for but no animals were removed. Removal of SNPs included SNPs with rare (<0.01) minor allele frequencies (*n* = 5141) and those that failed (*p* < 10 × 10^−50^) Hardy–Weinberg equilibrium testing (*n* = 556). After quality control, 2013 heifers and 611,685 SNPs remained for the analysis.

### 2.4. Genome-Wide Association Analysis

A GWAA was performed for HCR1 and TBRD using an efficient mixed-model association eXpedited statistical approach with an identity-by-state kinship matrix (EMMAX-IBS) using the SNP and Variation Suite (SVS) software v 8.1 (Golden Helix, Bozeman, MT, USA). The GWAA was performed using an additive, dominant and recessive model. The model is defined as  y=Xβ+Zu+ϵ, where *y* explains the *n* × 1 vector of observed phenotypes; X is a *n* × *f* matrix of fixed effects (*f*); β is a *f* × 1 vector containing the fixed effect coefficients; Z  is a *n* × *t* matrix relating the random effect (*t*) to the phenotype; and u is the random effect to the mixed model [12]. The model assumes residuals to be independent with an identical distribution such that $Var\left(u\right)=\sigma_{g}{}^{2}K$ and $Var\left(\epsilon \right)=\sigma_{e}{}^{2}I$, and such that $\;Var\left(y\right)=\sigma_{g}{}^{2}ZKZ^{\prime }+\sigma_{e}{}^{2}I\;$ [13]. For this study, *K* is a matrix of pairwise genomic relationships and *Z* is the identity matrix, *I* [14]. Associations (*p* < 5 × 10^−8^) were tested for, and evidence for significance was based on the International HapMap Consortium [15,16]. The genomic inflation factor lambda (λ_gc_) was calculated for each model to identify any potential biases present within the association results. Lambda is defined as the median of the observed median chi-squared test statistics divided by the expected median of the chi-squared distribution [17].

An ANOVA was performed to determine whether the year (2010 to 2015) or season (winter—December, January and February; spring—March, April and May; summer—June, July and August; and fall—September, October and November) when bred affected the conception rate. There was no difference in conception rate by year (*p* = 0.47) or by season (*p* = 0.41) in the population. The service sire used to breed the heifers influenced conception rate (*p* = 5.3 × 10^−6^) and the choice of service sires was influenced by the dairy that owned the heifers. Fortunately, heifers inseminated multiple times were rarely bred to the same sire on subsequent breedings, reducing the potential for repeated service sire bias. The use of the dairy that owned the heifers as a covariate corrected for these differences. The AI technician was also a possible source of differences in conception rate between heifers. There were 14 technicians performing AI in the Group 1 heifers and no differences in conception rates were observed (*p* = 0.14). No information was available to test if technicians affected the conception rates in Group 2 heifers.

Boundaries between loci were identified by using a linkage disequilibrium (LD) threshold of D’ > 0.7. Any SNP in LD with a SNP associated with fertility was considered to encompass the same locus. The D’ threshold was chosen as it falls within thresholds previously used to characterize SNPs within a locus [18,19,20]. Heritability was estimated using the average information algorithm (AI-REML), which is computationally demanding but commonly used [18]. This method uses a low-rank representation of the identity-by-descent matrix in SVS [21]. Ensembl [22] was used to determine if loci associated with fertility were within copy number variants (CNVs).

### 2.5. Positional Candidate Genes

To identify positional candidate genes within loci associated with fertility, the average haplotype block size of 19 kb for the Holstein heifers was calculated as described by Gabriel (2002) [23]. Genes within 19 kb of the associated SNP (5′ or 3′) were identified as positional candidate genes based on the bovine UMD 3.1 genome assembly [24].

### 2.6. Transcription Factor Binding Sites

PROMO and TRANSFAC were used to identify the presence of TFBS within 15 bp 5′ or 3′ of SNPs associated with HCR1 or TBRD [25,26,27]. The identification of TFBS was done twice, once with each SNP allele to determine if the SNP allele altered the TFBS. When one of the SNP alleles altered the TFBS, the random expectation query significance threshold was *p* < 0.05.

### 2.7. Pathway Analysis

Relationships between positional candidate genes and TFBS were examined using ingenuity pathway analysis (IPA; Qiagen, Redwood City, CA, USA). A total of 552 unique positional candidate genes and 52 unique TFBS were included in the IPA to identify the upstream regulators, master regulators, and canonical pathways. The canonical pathway analysis determines networks that are affected by gene expression changes, while the analyses for master and upstream regulators investigate molecules that indirectly and directly affected gene expression (for a review see Krämer et al. [28]). Significance thresholds were determined for each analysis using a Bonferroni correction. Significance thresholds were *p* < 1.25 × 10^−5^ for the upstream regulators, *p* < 1.23 × 10^−5^ for the master regulators and *p* < 1.33 × 10^−4^ for the canonical pathways. Significance thresholds varied due to the total number of pathways or molecules under evaluation.

## 3. Results

### 3.1. Genome-Wide Association Analysis

**Figure 1:**
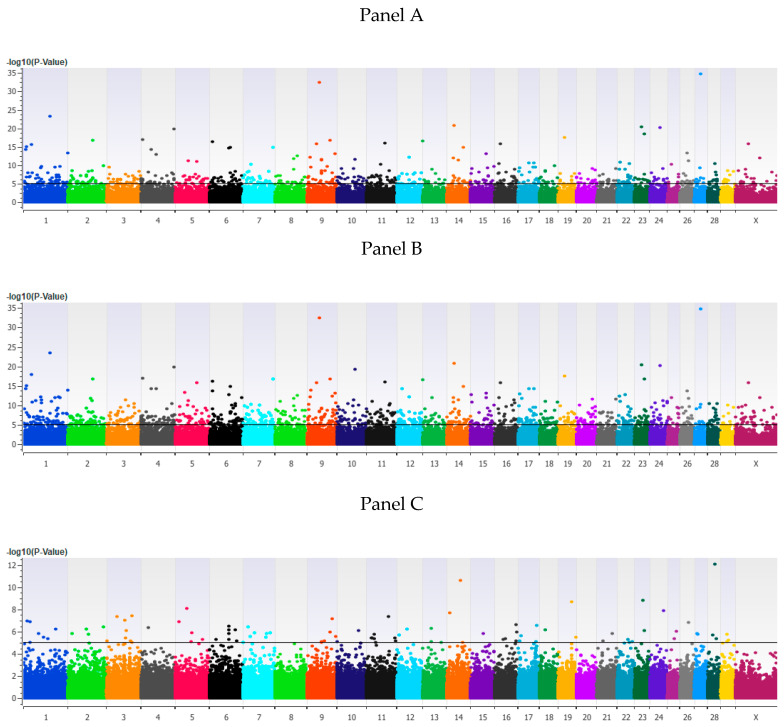
Loci associated with heifer conception rate at first service in Holstein heifers. Loci associated with heifer conception rate in an additive model (**Panel A**), a dominant model (**Panel B**) or a recessive model (**Panel C**) using efficient mixed-model association eXpedited statistical approach with an identity-by-state kinship matrix (EMMAX-IBS) and an uncorrected significance threshold of *p* < 5 × 10^−8^ (black line). All figures have bovine chromosomes on the *x* axis with −log10 *p* values on the *y* axis. As all of the animals were heifers, the Y chromosome is not shown.

The estimated heritability for HCR1 was 0.36 ± 0.04. The GWAA for HCR1 identified 146, 317 and 9 loci associated (*p* < 5 × 10^−8^) with the additive, dominant and recessive models, respectively (Figure 1 and Table S1). The genomic inflation factor lambda (λ_gc_) calculated for each model and both phenotypes indicated that there were no inherent biases in the associated results. The additive, dominant and recessive models for HCR1 had lambda values of 0.98, 0.98 and 1.00, respectively. The same models had values of 0.98, 0.98 and 0.97, respectively, for TBRD.

A total of 514 positional candidate genes and 98 TFBS were associated within the HCR1 loci (Tables S1 and S3). There were 114 loci shared between the additive and dominant models, one locus shared between the additive and recessive models and, as expected, no loci shared between the dominant and recessive models. Of the loci associated with HCR1, 42 loci overlapped with CNVs (Table S1). There were no loci within CNVs identified in the recessive model although 12% of the loci in the dominant model and 9% of the loci in the additive model were within CNVs. As CNVs account for only 2.5% of the bovine genome, CNVs are over-represented in loci associated with HCR1 [29].

**Figure 2:**
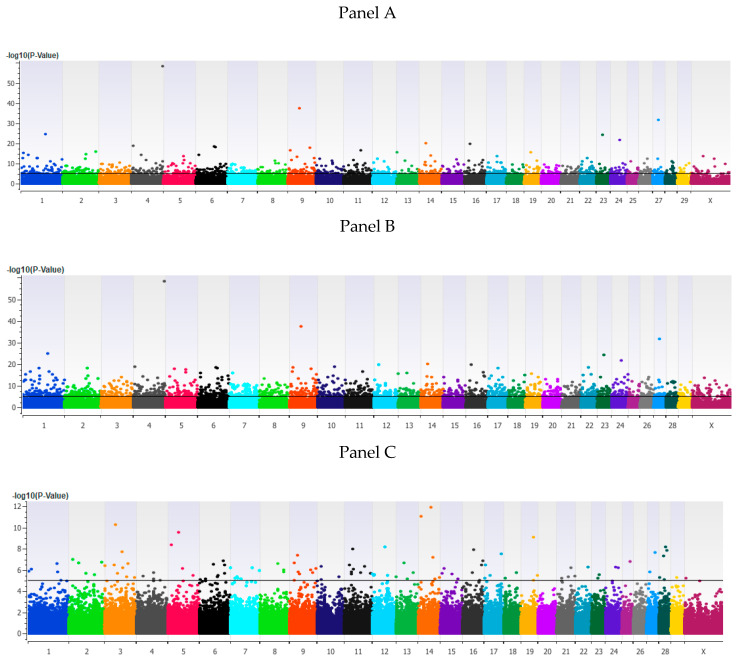
Loci associated with the number of times bred to achieve a pregnancy in Holstein heifers. Loci associated with the number of times bred to achieve a pregnancy in Holstein heifers in an additive model (**Panel A**), a dominant model (**Panel B**) or a recessive model (**Panel C**) of EMMAX using an IBS matrix and an uncorrected significance threshold of *p* < 5 × 10^−8^ (black line). All figures have bovine chromosomes on the *x* axis with −log10 *p* value on the *y* axis. As all of the animals were heifers, the Y chromosome is not shown.

The estimated heritability for TBRD was 0.53 ± 0.04. The GWAA for TBRD identified 246, 579 and 16 loci associated (*p* < 5 × 10^−8^) with additive, dominant and recessive models, respectively (Figure 2 and Table S2). The additive and dominant models shared 174 loci, while the additive and recessive models shared 3 loci (Table S2). As expected, no loci were shared between the dominant and recessive models for TBRD. A total of 360 positional candidate genes and 52 TFBS were identified in the loci associated with TBRD (Tables S2 and S3). Of the 615 unique loci associated with TBRD, 74 loci were present within CNVs (Table S2). The CNVs were almost equally divided between models, with 10%, 12% and 12% of loci identified in the additive, dominant and recessive models, respectively.

**Figure 3:**
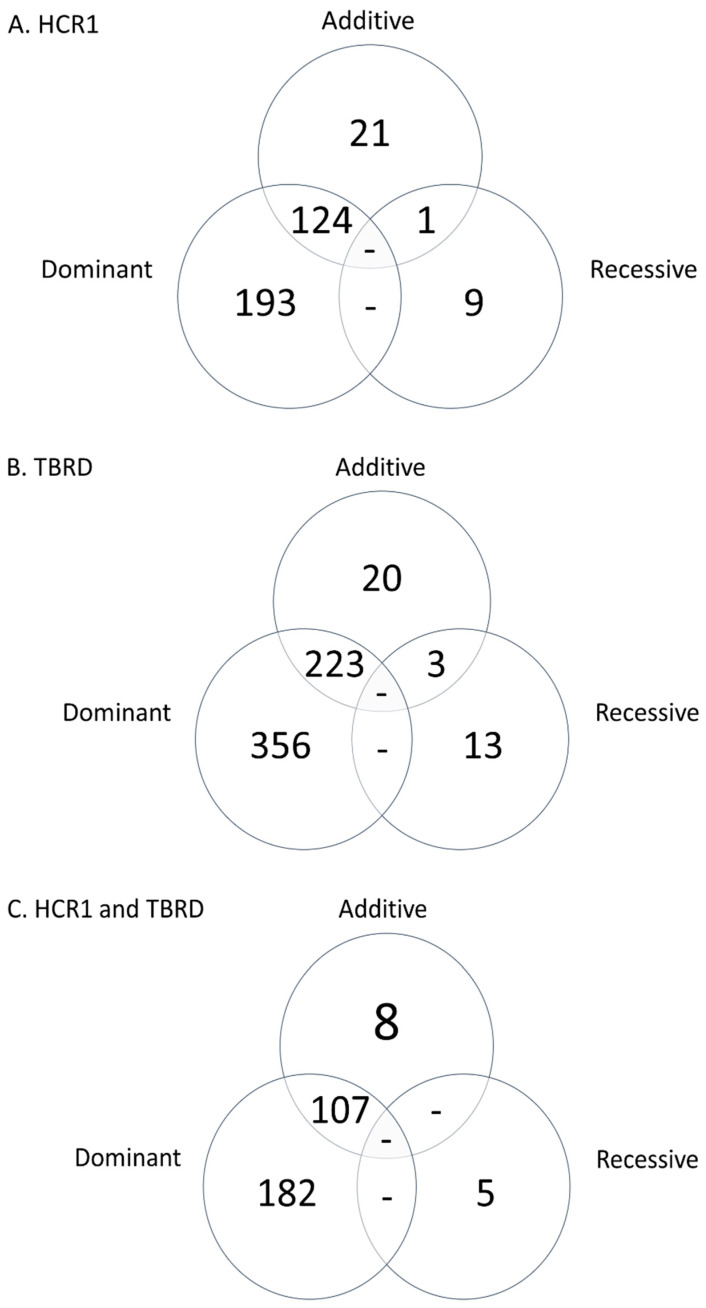
Venn diagram of the additive, dominant and recessive loci associated with conception rate to first service (HCR1) and the number of times bred (TBRD) to achieve a pregnancy in Holstein heifers. The number of loci associated in each model for heifer conception rate to first service (HCR1; **Panel A**), number of times bred before a pregnancy was achieved (TBRD; **Panel B**) and number of loci associated that were shared in HCR1 and TBRD (**Panel C**) are shown.

When the loci in HCR1 and TBRD were compared, 302 loci were shared across all models (Table 1 and Figure 3). The ten most significant loci shared across phenotypes were located on BTA1, BTA4, BTA5, BTA9, BTA10, BTA14 (two loci), BTA19, BTA23 and BTA27 (Table 1). Positional candidate genes for the ten most significant loci shared between HCR1 and TBRD were identified on BTA1 (*NLGN1*), BTA4 (*DPP6*), BTA5 (*MUC19*), BTA19 (*EFCAB3*) and BTA23 (*PKHD1*). The additive and dominant model for HCR1 and TBRD shared eight of the ten most significant loci (Table 1). The recessive model for HCR1 and TBRD shared five of the ten most significant loci (Table 1).

### 3.2. Validation of Associated Loci

Loci associated with HCR1 or TBRD were compared to fertility studies with independent cattle populations for validation (Table 2). To determine if loci were shared (validated), the SNP with the most significant association at each locus, in each study, was evaluated to identify if it was in LD by using a threshold of D’ > 0.70, as previously described [18,31,32]. In total, 56 loci associated with either HCR1 or TBRD were validated (Table 2). These validated loci represent robust associations with fertility as the phenotypes were highly diverse and the studies represented multiple cattle breeds (Holsteins, Nordic Jersey, Nordic Red, Fleckvieh and Angus) and geographical locations in the Holstein populations. The identification of loci across fertility phenotypes and breed is significant as it indicates that the loci have broad effects on fertility and may be close to causal mutations due to the variable LD distances across breeds surrounding each associated SNP. Discovering causal mutations associated with fertility would allow for major improvements in genomic selection for the cattle industry, and a better understanding of the molecular mechanisms associated with pregnancy maintenance and loss.

Comparisons were also made with positional candidate genes identified by the GWAA and studies that have identified genes that were differentially expressed in the endometrium of pregnant and nonpregnant fertility-classified cattle [43,44]. There were 31 positional candidate genes that were differentially expressed in the endometrium of pregnant and nonpregnant fertility-classified cattle (Table 3). Some of the differentially expressed genes have functions relating to fertility through cellular signaling and regulation of the immune system (*PLCB1, PIK3R1, EPHA3* and *CD109*) or embryonic development (*TIAM1* and *SYNE1*). Others had functions relating to cellular adhesion (*ABLIM3, CADPS, MEG3, SDK2, STC1* and *STX16*), DNA binding and repair (*AHDC1* and *POLD3*)*,* cellular signaling events (*DCP1A, FNIP2, PIK3R1, PKHD1, PLCB1* and *NTRK2*) and metabolism (*FHIT*) [45,46,47,48,49,50,51,52]. Unfortunately, some of the differentially expressed candidate gene functions are still uncharacterized *(CHD9, KIAA0825, MAP6, PRKG1, RAB3C, ROBO1, UPK1B* and *ZCCHC14)*.

### 3.3. Pathway Analysis

Positional candidate genes and TFBS associated with HCR1 and TBRD were used to identify the canonical pathways associated with fertility. Eleven canonical pathways were identified through IPA (Table 4). The three most significant canonical pathways had functions relating to nervous system signaling, cytokine signaling and cellular immune response. Eleven upstream regulators were identified that directly affect the positional candidate genes by altering gene expression, cell cycle, and cellular assembly, organization, maintenance and development (Table S4). Three out of the five most significant pathways (Neuropathic Pain Signaling in Dorsal Horn Neurons, *p* = 0.002; PI3K Signaling in B Lymphocytes, *p* = 0.002; Role of Macrophages, *p* = 0.003; and Fibroblasts and Endothelial Cells in Rheumatoid Arthritis, *p* = 0.004) are involved in the immune system regulation.

There were 207 master regulators affecting positional candidate genes and TFBS within loci associated with HCR1 and TBRD (Table S5). Master regulators act through upstream regulators to change gene expression. The most significant master regulator molecules were cannabinol (*p* = 2.01 × 10^−9^), RGS1 (*p* = 1.7 × 10^−8^) and WWC1 (*p* = 1.74 × 10^−8^). This analysis also identified 11 upstream regulators with the three most significant upstream regulators being calcium (*p* = 4.17 × 10^−8^), MT2 (*p* = 2.08 × 10^−7^) and LIF (*p* = 5.12 × 10^−7^; Table S4).

## 4. Discussion

The identification of SNPs associated with fertility traits such as HCR1 and TBRD is a starting point to determine the causal mutations responsible for specific phenotypes. Causal mutations are most frequently identified in regions associated with gene regulation that are outside of the gene itself but are in linkage disequilibrium with the regulated gene [53]. The identification of the positional candidate genes or regulatory regions such as TFBS, found within a haplotype block of the associated SNP, are potential sites for causal mutations or are sites likely to be affected by a causal mutation. Additionally, SNPs and the regions around them that are common to multiple phenotypes are more likely to have large effects on fertility through changes in gene regulation. It is therefore crucial to identify the shared DNA regions associated with fertility in multiple studies or phenotypes as mutations within those regions are likely to contain causal mutations that alter gene function, and ultimately reproduction.

The five positional candidate genes (*USH2A*, *VWC2*, *DPP6*, *NLGN1* and *PKHD1*) shared between HCR1 and TBRD are involved in cellular signaling and cell–cell interactions. The SNPs associated with these genes were all found within exons or introns as opposed to flanking regions. One of the most significant SNPs associated with HCR1 and TBRD on BTA1 was located approximately 1 kb from Neuroligin 1 (*NLGN1*), which produces neuronal cell surface proteins and is involved in cellular signaling changes (Table S2). The uterine epithelium contains a wide variety of adhesion molecules, which become particularly important during early pregnancy for conceptus implantation [54,55,56]. During the establishment of pregnancy and implantation, the uterine epithelium undergoes many changes. Mutations in *NLGN1* may produce proteins that disrupt the transportation of cell adhesion molecules to endometrial sites, resulting in early embryonic loss.

Dipeptidyl peptidase-like protein 6 (*DPP6*) is a positional candidate gene containing an associated SNP within an intron and produces a protein that is involved in cellular signaling and adhesion (Table 1) [57]. Dipeptidyl peptidase-like protein 6 codes for a single-pass type II transmembrane protein that binds to voltage-gated potassium channels from the Kv4 family [58,59]. Potassium channels play an important role in fertility due to their actions on uterine function, adaptation to pregnancy and contractility [60,61,62]. Early pregnancy is characterized by the suppression of uterine motility and high concentrations of progesterone, whereas late pregnancy is dependent on increased motility and estrogen during the last 14 days of pregnancy [63]. Estradiol has a stimulatory effect on potassium channels causing increased excitability, whereas progesterone has an inhibitory effect [55]. The inhibition of potassium channels due to increased progesterone levels leads to reduced contractions necessary for maintenance of pregnancies. Mutations in *DPP6* could lead to dysregulation of myometrial contractions potentially resulting in pregnancy loss.

The SNP associated with HCR1 and TBRD on BTA5 is located within an intron of Mucin 19 (*MUC19*) and is a member of the mucin glycoprotein family, which are responsible for the gel-like properties of mucus. The mucin 19 protein is abundantly expressed in the vagina, cervix and endometrium, as well as in the luminal epithelium [64]. The anti-adhesive properties of mucin 19 can block embryo attachment, so *MUC1* is downregulated by the blastocyst to reduce the mucins present on the epithelial cells in order to facilitate implantation [65,66]. Mutations in *MUC19* may lead to an overstimulation of anti-adhesive properties during the implantation window, thus causing early embryonic loss.

The most significant SNP associated with HCR1 and TBRD on BTA19 was located within an intron of *EFCAB3* (Table 1 and Table S1). The positional candidate gene, *EFCAB3*, produces a calcium-binding protein called EF-hand calcium-binding domain 3 [67]. In the myometrium, changes in calcium signals regulate contractility, which is critical both during the early and late stages of pregnancy. Increased uterine contractility during early pregnancy often results in pregnancy loss as normal regulation of calcium is crucial in maintaining uterine quiescence during early pregnancy [68]. Additionally, calcium is a secondary messenger to various pathways regulating reproductive processes, such as implantation and placentation [69] 

The fibrocystin or polyductin gene (*PKHD1)* on BTA23 harbors a SNP within an intron that is associated with HCR1 and TBRD. The fibrocystin gene produces a protein that spans the membrane of kidney cells and acts as a receptor by binding cells participating in the FAK/Src pathway (Table 1 and Table S1) [70,71]. This pathway regulates cell proliferation, migration and invasion during pregnancy by changing the expression of adhesion molecules E-cadherin and MMP2 in trophoblast cells [72,73,74]. Mutations in *PKHD1* may affect the FAK/Src signaling pathway leading to implantation failure. Variants of *PKHD1* are associated with chronic inflammation [40]. The regulation of immune response and inflammation that is crucial for normal pregnancy establishment may be disrupted by dysregulation of *PKHD1* through its effect on tumor necrosis factor-α (TNF-α). Tumor necrosis factor-α is an important inflammatory cytokine that plays essential roles in steroidogenesis, uterine cyclicity and placental differentiation. However, its over-expression can also be detrimental to pregnancies if it increases apoptosis, leading to an inhibition of the development of the fetus as seen in the mouse [52]. Therefore, the regulation of concentrations of inflammatory cytokines, such as TNF-α, due to a mutation in *PKHD1* may lead to early pregnancy losses.

### 4.1. Supporting SNPs in Fertility Associated Loci

In most of the GWAA results, the SNP defining an associated locus is accompanied by nearby supporting associated SNPs that create SNP association “trees”. However, many of the SNPs associated with HCR1 and TBRD did not show the expected surrounding associated SNPs. Several factors may account for this lack of supporting associated SNPs. Traits with dominant or recessive effects may not exhibit the typical pattern expected from additive effects. Associations were identified using three different models to distinguish the gene effects for each locus. The majority of loci associated with HCR1 and TBRD demonstrated dominant effects, and many exhibited dominant and additive effects although the significance tended to be greater with the dominant model. For loci associated with fertility in the recessive model, a similar pattern was present where some loci were associated in the recessive and additive models with the significance tending to be greater with the recessive, rather than the additive model.

The presence of a region of SNPs in association with fertility requires a strong LD between the surrounding SNPs. A lack of LD could explain the lack of supporting associated SNPs and represent a DNA region sparsely represented by SNPs. The sparseness of SNPs around the associated loci was investigated and revealed that there was a sufficient number of SNPs flanking each locus. Therefore, a lack of LD due to SNP density was not the cause for the paucity of supporting SNPs.

Other factors, such as CNVs, may influence the presence of associations in flanking SNPs. As many of the loci associated with HCR1 and TBRD were within CNVs, the disruption of LD around CNVs may have resulted in a lack of supporting SNP associations at these loci. The CNVs may alter the LD of flanking and associated SNPs if surrounding SNPs lie outside of the DNA region that is duplicated in a CNV. In this study, our estimates of the loci affected by CNVs are conservative, as only SNPs that were within a CNV were identified, even though the associated SNPs near a CNV breakpoint would also potentially disrupt LD. Therefore, more loci associated with the phenotypes may be affected by CNVs than are reported in this study. Previous studies identified specific CNVs associated with reproductive performance in Nordic Red and in *Bos indicus* cattle [52,75]. Copy number variants are also over-represented in the olfactory receptor gene family that has been associated with uterine capacity in beef cattle and has roles related to cell–cell communication during embryogenesis, tissue growth and regeneration, leading to additional support for their role in fertility [76,77].

Finally, a lack of a cluster of associated SNPs within a DNA region can be indicative of false positives. False positives are more likely to occur with a low association significance threshold, with rare minor allele frequencies, or if the locus has never been identified in similar studies. To determine if rare minor allele frequencies led to spurious results with a single SNP association in a DNA region, the minor allele frequency of the associated SNPs was determined. Three fertility-associated loci on BTA4 (*rs137620917*), BTA9 (*rs41609220*) and BTA27 (*rs132728892*) were identified to have rare minor allele frequencies (0.01–0.04). Therefore, the associations of these loci should be further investigated. To reduce false positives due to a low association significance threshold, a stringent association significance threshold was used (*p* < 5 × 10^−8^). Additionally, to reduce the possibilities of false positive associations, loci associated with HCR1 and TBRD were compared to loci associated with fertility in independent cattle populations and 56 were validated as they were identified in multiple populations.

The lack of supporting SNPs surrounding many of the loci identified as associated with HCR1 and TBRD is unique. Further examination of the results identified that this may be due to several factors. The mode of inheritance and the presence of associated loci within CNVs contributed significantly to this lack of supporting SNPs. Three hundred and twenty-seven HCR1-associated loci and 595 TBRD-associated loci were defined as dominant or recessive, where the presence of SNP trees is not expected. Copy number variants represented 42 loci associated with HCR1 and 74 loci associated with TBRD. An additional 56 loci associated with HCR1 and/or TBRD were supported by independent association studies, reducing the probability that these findings represent a false association. This leaves 30 loci associated with HCR1 and/or TBRD remaining that are not supported by a cluster of associated SNPs, including the 3 loci associated with fertility on BTA4, BTA9 and BTA27 that had rare minor alleles. The association of these 30 loci without surrounding associated SNPs is unexplained and warrants that their association with fertility be interpreted with caution until their association is validated in an independent population of cattle.

### 4.2. Ingenuity Pathway Analysis

Many of the regulators and pathways identified had functions involving the immune system. Immunity during pregnancy is of crucial importance, as altered immune function is one of the main causes of pregnancy loss [78]. The immune system of the pregnant heifer needs to accept the genetically foreign fetus and so failure of the heifer to recognize pregnancy can lead to reproductive failure. This carefully controlled process involves macrophages, B lymphocytes and pro- and anti-inflammatory cytokines [78].

The master regulator cannabinol is a chemical compound found in cannabis and is an indirect regulator of *AP-1* [79]. The AP-1 complex consists of a heterodimer of FOS and JUN and is involved in regulation of cell proliferation, differentiation and apoptosis [77]. The *C*-*FOS* and *JUN* proteins are involved in the regulation of the catabolite activator protein (*CAP)* gene in the myometrium that is responsible for the onset of labor [80]. Expression of *C-FOS* and *JUN* genes are low during early gestation and increase until parturition. Expression of *C-FOS* and *JUN* was altered with the administration of progesterone, suggesting that these transcription factors are necessary for the onset of parturition and are at least partially regulated by steroid hormones [80].

The second most significant master regulator, regulator of G protein signaling 1 (*RGS1)*, is responsible for decreasing the activity of G-protein signaling by binding to the α subunit of G proteins to increase the conversion of guanosine triphosphate (GTP) to guanosine diphosphate (GDP) [81]. In fertility, G-proteins are involved in GnRH signaling, which is necessary for normal ovulation and production of steroid hormones. Additionally, G-protein-coupled receptors are involved in the response of B lymphocytes, which are an important player in the maternal immune response to pregnancy. The *RGS1* proteins downregulate the signaling of B lymphocytes and affect the migration of lymphoid cells. This suggests *RGS1* may play a role in regulating the maternal immune response. Mutations of *RGS1* may lead to early embryonic loss due to impairment of cell signaling and immune system regulation.

The third most significant master regulator, the WW and C2 domain-containing 1 (*WWC1*) gene, produces a phosphoprotein. It has functions in restricting proliferation and promoting apoptosis, as well as in regulating transcription of estrogen receptor α (*ESR1*) [82]. The *WWC1* gene is part of the hippo pathway, which also regulates cell proliferation and apoptosis and plays a role in position sensing and lineage specification in preimplantation embryos [83,84]. Disruption of this pathway results in failure of embryo development post-implantation.

Upstream regulators have direct effects on target genes. The most significant upstream regulator is calcium, which has effects on uterine contractility and implantation. A study conducted by Banerjee et al. [85] reported that blocking calcium channels interferes with normal implantation. This is due to the need for calcium influx into smooth muscle vascular cells that leads to the activation of prostaglandin synthesis [82]. Prostaglandin is an important mediator of the inflammatory response to allow implantation of the embryo [84].

The second most common upstream regulator is the melatonin receptor (*MT2*). The melatonin receptor is a G-protein-coupled receptor that carries out the effects of melatonin. Differential expression of *MT2* influences myometrial contractility by acting through the oxytocin receptor and enhancing contractions [85]. Melatonin increases estrogen concentrations, thus favoring implantation, and has effects on embryo quality by indirectly reducing the rate of apoptosis [82]. Reductions in melatonin and expression of its receptors has been linked to pregnancy complications and neonatal neurological disabilities [86]. Finally, melatonin acts on p53, a tumor-suppressor protein, in order to regulate the cell cycle and control the estrogen mediator leukemia inhibitory factor *(LIF*) by regulating transcription.

The LIF protein is crucial for implantation and is the third most common upstream regulator in this analysis. Leukemia inhibitory factor production has been linked to embryo implantation due to its effects on uterine receptivity, decidualization in mice, blastocyst growth, interaction between the embryo and endometrium and maternal immunity changes [87]. Leukemia inhibitory factor carries out its many actions through the JAK/STAT and PI3K/AKT pathways, which were involved in many of the canonical pathways associated with fertility [88].

Overall, the functions of the canonical pathways, master regulators, upstream regulators and target genes reveal the complexity of mechanisms involved in pregnancy establishment. The recurrent biological activities included cell proliferation, migration and adhesion control, as well as regulation of the immune system. These processes are all crucial to proper implantation, placentation and communication between embryo and dam. This suggests that these events should be the focus of functional studies to determine how to improve the heifer conception rate most effectively.

## 5. Conclusions

This study focused on identifying and analyzing the loci associated with heifer conception rate in U.S. Holstein cattle. The GWAA revealed a multitude of loci associated with HCR1 and TBRD. Fifty-six loci identified in this study were associated with fertility phenotypes in independent cattle populations. The validation of the fertility loci across studies, phenotypes and breeds make them excellent candidates for genomic selection and ultimately to identify causal mutations that alter fertility. Further analysis of positional candidate genes and TFBS using IPA established the importance of regulation of early pregnancy events, such as implantation and placentation. Understanding the interaction of the positional candidate genes and TFBS in associated loci is a crucial step towards choosing the best candidates for further functional analyses and achieving a comprehensive view of early embryonic loss in heifers. Overall, this study provides a foundation to improve the accuracy of genomic selection in the dairy industry and to better understand fertility and embryonic loss.

## Figures and Tables

**Table 1:** The ten most significant loci shared between heifer conception rate at first Service (HCR1) and the number of times bred to achieve a pregnancy (TBRD).

BTA ^1^	BP Position ^2^	SNP ID ^3^	Model ^4^	HCR1 *p*-Value ^5^	TBRD *p*-Values ^6^	Positional Candidate Genes ^7^
1	94,300,486	*rs41606324*	additive dominant	6.28 × 10^−24^ 3.65 × 10^−24^	3.97 × 10^−25^ 2.01 × 10^−25^	*NLGN1*
4	117,606,629	*rs137620917*	additive dominant	1.47 × 10^−20^ 1.47 × 10^−20^	4.67 × 10^−59^ 4.67 × 10^−59^	*DPP6*
5	40,576,651	*rs43434026*	recessive	8.48 × 10^−9^	3.03 × 10^−10^	*MUC19*
9	45,148,825	*rs41609220*	additive	4.89 × 10^−33^	5.72 × 10^−38^	*-*
10	63,217,046	*rs133388647*	dominant	6.98 × 10^−20^	1.36 × 10^−19^	*-*
14	25,633,578	*rs134826452*	additive dominant	1.67 × 10^−21^ 1.67 × 10^−21^	7.83 × 10^−21^ 7.83 × 10^−21^	*-*
14	50,291,072	*rs41913814*	recessive	2.64 × 10^−11^	1.21 × 10^−21^	*-*
19	47,475,942	*rs41917870*	recessive	2.14 × 10^−9^	8.34 × 10^−10^	*EFCAB3*
23	23,965,902	*rs41634508*	additive dominant	4.77 × 10^−21^ 4.77 × 10^−21^	5.55 × 10^−25^ 5.55 × 10^−25^	*PKHD1*
27	21,375,791	*rs132728892*	additive dominant	1.84 × 10^−35^ 1.84 × 10^−35^	2.90 × 10^−32^ 2.90 × 10^−32^	-

^1^ Chromosome location of the locus. ^2^ Single nucleotide polymorphism (SNP) location as measured by numbered nucleotides (base pairs; bp) in reference to the UMD 3.1 genome assembly [24]^3^ The most significant SNP in the locus associated with heifer conception rate is listed. The *rs* number is the assigned SNP identity from the National Center for Biotechnology Information SNP database [30]. ^4^ Genome-wide association analysis model. ^5^ Significance (*p-*value) of the most significant SNP associated with heifer conception rate at first service (HCR1). ^6^ Significance (*p-*value) of the most significant SNP associated with number of times bred to achieve a pregnancy (TBRD) in heifers. ^7^ Positional candidate genes are genes located within 17.8 kb 5′ or 3′ of the associated SNP(s).

**Table 2:** Loci associated with bovine fertility in previous studies.

BTA ^1^	BP Position ^2^	SNP ID ^3^	Study ^4^	Trait in Other Studies ^5^
1	1–6	*rs136767715*	Pausch et al. 2015 [33]	FH
1	25–27	*rs133334228*	Moore et al. 2016 [34]	GMF
1	129–130	*rs136894301*	Cole et al. 2011 [35]	DPR
1	61–62	*rs42760220* *rs42675527*	Hoglund 2014 [36]	AISC
1	61–63	*rs43652271*	Fonseca et al. 2018 [37]	PLAF
1	84–85	*rs109101339*	Hoglund 2014 [36]	AISC/AISH
1	94–96	*rs135160436*	Hoglund 2014 [36]	AISH
1	88–89	*rs109891698*	Hoglund 2014 [36]	AISC
3	32–33	*rs43336503*	Fonseca et al. 2018 [37]	PLAF
3	119–121	*rs41615294*	Hoglund 2014 [36]	AISH
3	96–97	*rs41585055*	Hoglund 2014 [36]	AISC
3	98–99	*rs43356386*	Hoglund 2014 [36]	AISC
4	29–30	*rs43181427*	Hoglund 2014 [36]	AISC
4	37–38	*rs133417267*	Fonseca et al. 2018 [37]	PLAF
5	88–89	*rs137120693*	Nayeri et al. 2016 [38]	CTFS
6	92–94	*rs110063753* *rs43477811*	Hoglund 2014 [36]	AISC
7	12–13	*rs133465587*	Cole et al. 2011 [35]	FH
7	57–59	*57,914,820*	Hoglund 2014 [36]	AISC
8	59–61	*rs134066757*	Hoglund 2014 [36]	AISC
8	72–74	*rs133191466*	Hoglund 2014 [36]	AISC
8	84–86	*rs43137599*	Hoglund 2014 [36]	AISC
9	28–30	*rs109636996*	Hoglund 2014 [36]	AISC
9	61–61	*rs43601819*	Hoglund 2014 [36]	AISC
9	90–91	*rs43608400*	Hoglund 2014 [36]	AISC
10	26–35	*rs110325782*	Cole et al. 2011 [35]	FH
10	63–65	*rs133765760*	Hoglund 2014 [36]	AISC
11	17–18	*rs134981474*	Hoglund 2014 [36]	AISC
11	21–22	*rs43669974*	Moore et al. 2016 [34]	GMF
11	57–59	*rs42234541* *rs136444067*	Hoglund 2014 [36]	AISH
11	93–95	*rs134709354*	Hoglund 2014 [36]	AISC
11	101–102	*rs136026124*	Fonseca et al. 2018 [37]	PLAF
12	81–82	*rs135307240*	Fonseca et al. 2018 [37]	PLAF
13	45–46	*rs42628484*	Hoglund 2014 [36]	AISC
14	45–46	*rs41630614*	Moore et al. 2016 [34]	GMF
14	44–46	*rs136545426*	Minnozi et al. 2013 [39]	NR56
15	63–64	*rs135885524*	Fonseca et al. 2018 [37]	PLAF
16	16–17	*rs133881641*	Hoglund 2014 [36]	AISC
16	21–21	*rs108994652*	Neupane et al. 2017 [40]	ET
16	69–70	*rs42385478*	Hoglund 2014 [36]	AISC
17	14–15	*rs110372003*	Hoglund 2014 [36]	AISC
17	57–58	*rs137751476*	Hoglund 2014 [36]	AISC
18	25–26	*rs135881758*	Cochran 2013 [41]	DPR
18	25–26	*rs135881758*	Ortega et al. 2016 [42]	CCR
18	48–49	*rs137310621*	Minnozi et al. 2013 [39]	NR56
20	46–47	*rs41949865*	Hoglund 2014 [36]	AISC
20	53–54	*rs135839614*	Hoglund 2014 [36]	AISC
21	52–54	*rs137802601*	Nayeri et al. 2016 [38]	CTFS
26	6–8	*rs133146678*	Fonseca et al. 2018 [37]	PLAF
26	25–26	*rs110088444*	Hoglund 2014 [36]	AISC
26	40–41	*rs136057362*	Cole et al. 2011 [35]	FH
X	0–1	*rs42069602*	Cole et al. 2011 [35]	DPR

^1^ Chromosome location of the associated locus. ^2^ Location of shared loci as measured by numbered nucleotides (bp) in reference to the UMD 3.1 genome assembly [24]). ^3^ The *rs* number is the assigned SNP identity from the National Center for Biotechnology Information SNP database [30]. ^4^ Study in which the shared loci were identified. ^5^ Fertility traits used in each study: FH: Fertility haplotype; GMF: Genetic merit for fertility; DPR: Daughter pregnant rate; AISC: Number of inseminations per conception in cows; AISH: Number of inseminations per conception in heifers; PLAF: Pleiotropic loci associated with fertility; CTFS: Calving to first service; ET: Embryo transfer; NR56: Non return rate; CCR: Cow conception rate.

**Table 3:** Differentially expressed genes associated with heifer conception rate at first service (HCR1) and the number of times bred to achieve a pregnancy (TBRD).

Genes ^1^	Tissue ^2^	Phenotype ^3^
*ABLIM3*	Pregnant Endometrium HF vs. SF	TBRD
*AHCYL2*	HF vs. SF Conceptuses	TBRD
*AHDC1*	HF—Pregnant vs. Open -Endometrium	HCR1 TBRD
*CADPS*	SF—Pregnant vs. Open Endometrium	HCR1 TBRD
*CD109*	HF—Pregnant vs. Open Endometrium	HCR1 TBRD
*CHD9*	HF vs. SF Conceptuses	HCR1 TBRD
*DCP1A*	HF vs. SF Conceptuses	HCR1 TBRD
*EPHA3*	Pregnant Endometrium HF vs. SF	TBRD
*FHIT*	HF vs. SF Conceptuses	TBRD
*FNIP2*	HF vs. SF Conceptuses	HCR1 TBRD
*GRIA4*	HF—Pregnant vs. Open Endometrium	HCR1 TBRD
*KIAA0825*	HF vs. SF Conceptuses	TBRD
*MAP6*	HF—Pregnant vs. Open Endometrium	HCR1 TBRD
*MEG3*	HF—Pregnant vs. Open Endometrium HF vs. SF Conceptuses	HCR1 TBRD
*NTRK2*	Pregnant Endometrium HF vs. SF and HF—Pregnant vs. Open Endometrium SF—Pregnant vs. Open Endometrium	HCR1 TBRD
*PIK3R1*	HF—Pregnant vs. Open Endometrium	HCR1 TBRD
*PKHD1*	HF vs. SF Conceptuses	HCR1 TBRD
*PLCB1*	HF—Pregnant vs. Open Endometrium and SF—Pregnant vs. Open Endometrium	HCR1 TBRD
*POLD3*	SF—Pregnant vs. Open Endometrium	HCR1 TBRD
*PRKG1*	HF vs. SF Conceptuses	HCR1 TBRD
*RAB3C*	SF—Pregnant vs. Open Endometrium	TBRD
*ROBO1*	SF—Pregnant vs. Open Endometrium	TBRD
*SDK2*	HF—Pregnant vs. Open Endometrium	HCR1 TBRD
*SORCS3*	HF vs. SF Conceptuses	HCR1 TBRD
*STC1*	SF—Pregnant vs. Open Endometrium	TBRD
*STX16*	HF vs. SF Conceptuses	TBRD
*SYNE1*	HF—Pregnant vs. Open Endometrium	HCR1 TBRD
*TDRD1*	HF vs. SF Conceptuses	HCR1 TBRD
*TIAM1*	HF vs. SF Conceptuses	HCR1 TBRD
*UPK1B*	SF—Pregnant vs. Open Endometrium HF vs. SF Conceptuses Open Endometrium HF vs. SF	HCR1 TBRD
*ZCCHC14*	HF vs. SF Conceptuses	TBRD

^1^ Name of genes identified in Moraes et al. 2018 [44] and the current study. ^2^ Tissues in which the genes were differentially expressed—HF: Highly fertile; SF: Subfertile. ^3^ Phenotype in which the genes were identified in the current study.

**Table 4:** Canonical pathways identified by ingenuity pathway analysis for heifer conception rate at first service (HCR1) and the number of times bred to achieve a pregnancy (TBRD).

Ingenuity Canonical Pathways ^1^	Significance (*p*-Value) ^2^	Number of Target Molecules ^3^
Neuropathic Pain Signaling In Dorsal Horn Neurons	0.002213	12
PI3K Signaling in B Lymphocytes	0.002291	13
Axonal Guidance Signaling	0.003428	25
Role of Macrophages, Fibroblasts and Endothelial Cells in Rheumatoid Arthritis	0.003673	20
Prolactin Signaling	0.003981	10
VDR/RXR Activation	0.005821	9
Thrombin Signaling	0.005888	15
P2Y Purigenic Receptor Signaling Pathway	0.006026	12
Aryl Hydrocarbon Receptor Signaling	0.006237	12
Endothelin-1 Signaling	0.007943	14
Gap Junction Signaling	0.00912	14

^1^ Name of canonical pathways identified by ingenuity pathway analysis for HCR1 and TBRD. ^2^ *p*-value with Bonferroni correction (*p* < 1.33 × 10^−4^). ^3^ Positional candidate genes and transcription binding factor sites present in the canonical pathway.

## Supplementary Materials

The following are available online at https://www.mdpi.com/2073-4425/11/7/767/s1, Table S1: Loci Associated with Heifer Conception Rate at First Breeding, Table S2: Loci Associated with Number of Times Bred to Achieve Pregnancy; Table S3: Loci Associated with Conception Rate at First Breeding (HCR1) and The Number of Times Bred to Achieve Pregnancy (TBRD) that Contained Transcription Factor Binding Sites, Table S4: Upstream Regulators and their Targets Identified in the Ingenuity Pathway Analysis for Heifer Conception Rate, Table S5: Master Regulators and their Targets Identified in the Ingenuity Pathway Analysis for Heifer Conception Rate.
