# Supplementary material for: Identification of Loci and Pathways Associated with Heifer Conception Rate in U.S. Holsteins
Source: Genes (Basel). 2020 Jul 8;11(7):767. doi: 10.3390/genes11070767 (PMC7397161; doi:10.3390/genes11070767)
Supplement: Supplementary file 1 [file genes-11-00767-s001.pdf]

Supplementary Materials:

Supplementary Table 1. Loci Associated with Heifer Conception Rate at First Breeding

BTA[1]	UMD 3.1 Position[2]	SNP ID[3,4]	p-value[5]	Models[6]	Positional Candidate Genes[7]	Proportion of Variance Explained[8]
1	3,546,098	rs136767715	7.56 X 10^{-10}	dominant	TIAM1[‡]	0.02
1	7,862,063	rs135020930	6.11 X 10^{-15}<br>6.11 X 10^{-15}	additive<br>dominant		0.03<br>0.03
1	11,250,683	rs110647268*	9.77 X 10^{-16}<br>9.77 X 10^{-16}	additive<br>dominant		0.03<br>0.03
1	11,812,309	rs109447046	8.50 X 10^{-9}	dominant		0.02
1	16,446,932	rs110075902*	1.29 X 10^{-8}<br>3.07 X 10^{-8}	additive<br>dominant		0.02<br>0.02
1	29,618,772	rs43224096	2.35 X 10^{-16}<br>1.67 X 10^{-18}	additive<br>dominant		0.03<br>0.04
1	32,700,496	rs43226666*	1.09 X 10^{-8}<br>1.55 X 10^{-11}	additive<br>dominant	CADM2[‡‡]	0.02<br>0.02
1	45,719,982	rs43682172	5.72 X 10^{-12}	dominant	ABI3BP[‡]	0.02
1	62,056,267	rs42760220	3.48 X 10^{-9}	dominant		0.02
1	62,158,499	rs42675527	5.50 X 10^{-10}<br>6.96 X 10^{-13}	additive<br>dominant	LOC538060	0.02<br>0.03
1	64,591,504	rs43652271	2.57 X 10^{-10}<br>4.01 X 10^{-12}	additive<br>dominant	UPK1B[‡]	0.02<br>0.02
1	64,894,430	rs42911131	2.48 X 10^{-11}	dominant	CD80[‡]	0.02
1	86,830,692	rs110457263	1.40 X 10^{-8}	additive		0.02
1	94,300,486	rs41606324*	6.28 X 10^{-24}<br>3.65 X 10^{-24}	additive<br>dominant	NLGN1[‡]	0.05<br>0.05
1	94,998,936	rs135160436	1.17 X 10^{-8}	dominant	SPATA16[‡]	0.02
1	99,584,226	rs110848318	1.05 X 10^{-11}	dominant		0.02
1	112,961,101	rs110435444	4.04 X 10^{-10}<br>1.09 X 10^{-12}	additive<br>dominant	PLCH1[‡]	0.02<br>0.03
1	117,359,373	rs134846486	1.78 X 10^{-9}	dominant		0.02
1	122,822,402	rs110884711*	1.82 X 10^{-8}	dominant	PLSCR5[‡]	0.02
1	123,956,245	rs132851673	6.18 X 10^{-10}	dominant		0.02
1	126,235,693	rs136458590*	8.06 X 10^{-13}	dominant	SLC9A9[‡]	0.03

1	130,576,374	rs110960328	2.10 X 10^{-10}<br>1.00 X 10^{-12}	additive<br>dominant	KPNA6‡<br>LOC104971035‡<br>RBP1	0.02<br>0.03
1	139,950,787	rs43278206	1.25 X 10^{-8}	dominant	MRPL3	0.02
1	154,853,461	rs134813046	1.52 X 10^{-8}	dominant		0.02
1	157,576,622	rs136888926*	1.87 X 10^{-8}	dominant		0.02
1	158,010,340	rs43289085	4.94 X 10^{-14}<br>1.28 X 10^{-14}	additive<br>dominant		0.03<br>0.03
2	15,775,602	rs136049910	3.82 X 10^{-9}	additive	TRNASTOP-<br>UCA UBE2E3	0.02
2	32,783,586	rs135787342	4.20 X 10^{-8}	dominant	FIGN‡	0.01
2	36,425,765	rs136627254	2.78 X 10^{-8}	dominant	PLA2R1‡	0.02
2	65,493,191	rs109322207	4.40 X 10^{-8}<br>4.96 X 10^{-10}	additive<br>dominant		0.01<br>0.02
2	72,248,936	rs110344076	6.08 X 10^{-9}<br>6.08 X 10^{-9}	additive<br>dominant	EPB41L5‡	0.02<br>0.02
2	80,429,623	rs134254979	1.98 X 10^{-12}	dominant	LOC104971267‡	0.02
2	84,127,565	rs110922881*	8.14 X 10^{-9}	dominant		0.02
2	85,872,885	rs134179168	4.02 X 10^{-9}<br>7.47 X 10^{-12}	additive<br>dominant	PGAP1‡	0.02<br>0.02
2	87,872,337	rs41616585*	4.44 X 10^{-9}	dominant		0.02
2	88,137,677	rs135769112*	1.66 X 10^{-17}<br>1.66 X 10^{-17}	additive<br>dominant		0.04<br>0.04
2	126,326,843	rs132632350	1.22 X 10^{-10}	additive	AHDC1	0.02
3	7,783,286	rs135239306	3.61 X 10^{-10}	additive	ATF6‡	0.02
3	12,500,599	rs133108396	2.97 X 10^{-8}	additive	LOC531264	0.02
3	14,665,964	rs136725222	8.54 X 10^{-9}	dominant	SEMA4A‡	0.02
3	32,269,604	rs43336503	3.39 X 10^{-8}	dominant	CHI3L2‡	0.02
3	37,781,561	rs137663076	4.61 X 10^{-8}	recessive		0.01
3	38,557,710	rs109265034	1.36 X 10^{-8}	dominant		0.02
3	42,093,978	rs134200102	3.78 X 10^{-9}	dominant		0.02
3	49,686,970	rs136708725	3.25 X 10^{-10}	dominant	ABCA4<br>LOC783590	0.02
3	56,967,816	rs137196969	5.89 X 10^{-9}	dominant		0.02
3	66,777,217	rs133073532	3.25 X 10^{-8}<br>4.64 X 10^{-12}	additive<br>dominant	GIPC2<br>LOC100140103	0.02<br>0.02
3	77,163,315	rs136533238	1.25 X 10^{-10}	dominant	RPE65‡	0.02

3	89,365,476	rs137362504	3.77×10^{-8}	recessive	DAB1ǂ	0.02
3	97,680,312	rs41585055	3.86×10^{-10}	dominant	AGBL4ǂ	0.02
3	98,855,342	rs43356386	4.83×10^{-11}	dominant	LOC101906301 TRABD2B	0.02
3	101,247,684	rs43361651	1.28×10^{-8}	dominant	TESK2ǂ	0.02
3	116,328,811	rs133498551	4.36×10^{-9}<br>2.67×10^{-9}	additive<br>dominant	ASB18ǂ	0.02<br>0.02
4	5,954,962	rs137299312	1.04×10^{-17}<br>1.04×10^{-17}	additive<br>dominant	VWC2ǂ	0.04<br>0.04
4	17,611,774	rs41659165	6.09×10^{-9}	dominant	NXPH1ǂ	0.02
4	27,465,970	rs109930822	6.77×10^{-9}	additive	HDAC9ǂ	0.02
4	37,235,676	rs133417267	5.77×10^{-15}<br>5.77×10^{-15}	additive<br>dominant	SEMA3Eǂ	0.03<br>0.03
4	54,918,207	rs42421993	1.63×10^{-13}<br>5.31×10^{-15}	additive<br>dominant	PPP1R3A	0.03<br>0.03
4	93,277,289	rs109721084	8.71×10^{-10}	dominant	LEP	0.02
4	109,038,482	rs132930684	1.27×10^{-8}<br>4.59×10^{-11}	additive<br>dominant		0.02<br>0.02
4	110,200,691	rs110149102	4.48×10^{-8}	dominant		0.01
4	114,535,577	rs43415527	2.78×10^{-8}	additive	AGAP3 ASB10 GBX1ǂ	0.02
4	117,606,629	rs137620917	1.47×10^{-20}<br>1.47×10^{-20}	additive<br>dominant	DPP6ǂ	0.04<br>0.04
4	118,148,549	rs133872172	3.10×10^{-8}	dominant	RBM33ǂ	0.02
4	119,201,399	rs43420944	2.62×10^{-8}	dominant		0.02
5	17,173,888	rs134628502	2.76×10^{-10}	dominant		0.02
5	17,207,930	rs135145406	2.58×10^{-8}	dominant	LOC782748ǂ	0.02
5	29,350,321	rs110713926	1.63×10^{-8}	dominant	DIP2Bǂ	0.02
5	33,240,014	rs135651218	6.68×10^{-14}	dominant	PCED1Bǂ	0.03
5	40,576,651	rs43434026	8.48×10^{-9}	recessive	MUC19ǂ	0.02
5	44,853,575	rs134622788	8.77×10^{-11}	dominant		0.02
5	48,012,423	rs135945780	8.42×10^{-12}<br>8.42×10^{-12}	additive<br>dominant		0.02<br>0.02
5	64,125,159	rs110969021	2.60×10^{-10}	dominant	ANKS1Bǂ	0.02
5	72,903,919	rs132740823	2.44×10^{-8}<br>2.28×10^{-8}	additive<br>dominant		0.02<br>0.02
5	76,167,701	rs109300805*	2.11×10^{-9}	dominant	ELFN2	0.02

5	77,199,853	rs43439753*	1.14 X 10^{-11}<br>1.82 X 10^{-16}	additive<br>dominant		0.02<br>0.03
5	94,325,298	rs135948685	1.25 X 10^{-8}	dominant	DERA‡	0.02
5	107,407,965	rs133201447	5.13 X 10^{-10}	dominant	FOXM1 ITFG2<br>LOC104972569<br>LOC782076<br>NRIP2	0.02
5	111,790,193	rs109912186	2.03 X 10^{-9}	dominant	GRAP2‡	0.02
6	11,154,927	rs43452214	4.28 X 10^{-17}<br>6.63 X 10^{-17}	additive<br>dominant		0.03<br>0.03
6	11,301,952	rs29019244	7.11 X 10^{-10}	dominant		0.02
6	11,640,946	rs108963104*	2.42 X 10^{-14}	dominant		0.03
6	13,154,909	rs43450481	1.63 X 10^{-8}	dominant	CAMK2D‡	0.02
6	42,265,935	rs110203806	3.60 X 10^{-8}	dominant	KCNIP4‡	0.02
6	68,709,723	rs135139162	2.65 X 10^{-15}<br>1.89 X 10^{-13}	additive<br>dominant	SLAIN2‡	0.03<br>0.03
6	73,503,493	rs109467082	1.89 X 10^{-15}<br>1.89 X 10^{-15}	additive<br>dominant	PAICS‡ PPAT<br>SRP72	0.03<br>0.03
6	73,853,804	rs43468439	1.17 X 10^{-8}	dominant	REST‡	0.02
6	74,910,779	rs110648584	1.28 X 10^{-8}<br>5.82 X 10^{-9}	additive<br>dominant		0.02<br>0.02
6	78,372,811	rs136660262	4.77 X 10^{-9}	dominant	ADGRL3	0.02
6	81,047,108	rs43125352*	8.28 X 10^{-11}	dominant		0.02
6	85,034,144	rs29004040	3.93 X 10^{-8}<br>5.10 X 10^{-10}	additive<br>dominant	STAP1‡ UBA6	0.01<br>0.02
6	92,587,809	rs110063753	5.21 X 10^{-9}<br>1.44 X 10^{-10}	additive<br>dominant	CXCL9 SDAD1	0.02<br>0.02
6	93,263,835	rs43476570	3.35 X 10^{-11}	dominant	SHROOM3‡	0.02
6	93,290,585	rs43477811*	1.61 X 10^{-8}	dominant	SHROOM3‡	0.02
6	114,510,383	rs109630077	1.08 X 10^{-12}	dominant		0.03
7	6,118,322	rs132731242	4.64 X 10^{-8}<br>1.70 X 10^{-10}	additive<br>dominant	F2RL3 SIN3B‡	0.01<br>0.02
7	12,213,252	rs137569961	1.89 X 10^{-8}	dominant	ADGRE3‡	0.02
7	13,721,441	rs41588240	2.24 X 10^{-8}	dominant	GADD45GIP1‡	0.02
7	15,034,214	rs110743590	9.70 X 10^{-9}<br>3.89 X 10^{-10}	additive<br>dominant	LOC509510<br>LOC530825<br>LOC787383	0.02<br>0.02

7	19,341,834	rs110943465	$5.40 X 10^{-9}$<br>$2.45 X 10^{-8}$	additive<br>dominant	ACER1ǂ	0.02<br>0.02
7	28,375,798	rs43734065	$5.79 X 10^{-11}$<br>$5.66 X 10^{-11}$	additive<br>dominant	mar 3ǂ<br>LOC104969129ǂ	0.02<br>0.02
7	32,634,417	rs132887259	$3.70 X 10^{-9}$	dominant		0.02
7	54,413,989	rs137316807	$2.57 X 10^{-9}$	dominant		0.02
7	57,914,820	rs42282884	$1.04 X 10^{-10}$	dominant		0.02
7	58,055,640	rs133170783	$1.40 X 10^{-8}$	dominant		0.02
7	62,311,969	rs134893796	$1.27 X 10^{-8}$	dominant		0.02
7	64,472,010	rs43519903	$2.90 X 10^{-8}$	dominant	CCDC69	0.02
7	69,881,920	rs135934765	$4.36 X 10^{-8}$	dominant	SGCDǂ	0.01
7	75,775,268	rs43530606	$4.81 X 10^{-8}$	dominant	GABRA1	0.01
7	92,286,550	rs133820271	$5.35 X 10^{-9}$<br>$8.35 X 10^{-9}$	additive<br>dominant	CETN3<br>LOC101904644	0.02<br>0.02
7	93,415,439	rs136096285	$1.08 X 10^{-8}$	dominant	LOC104972872ǂ	0.02
7	108,044,827	rs132834691	$1.92 X 10^{-15}$<br>$2.44 X 10^{-17}$	additive<br>dominant		0.03<br>0.04
8	2,353,560	rs110390603	$1.26 X 10^{-9}$	dominant		0.02
8	12,163,981	rs136342892	$4.03 X 10^{-9}$	dominant		0.02
8	19,092,309	rs136161638*	$1.21 X 10^{-11}$	dominant		0.02
8	22,202,557	rs110186355	$1.05 X 10^{-8}$	dominant		0.02
8	27,874,312	rs136472729	$3.46 X 10^{-9}$	dominant	BNC2ǂ	0.02
8	28,583,167	rs133541436	$2.39 X 10^{-8}$	dominant	CCDC171ǂ	0.02
8	36,504,354	rs137050402	$5.23 X 10^{-9}$	dominant	PTPRDǂ	0.02
8	37,065,088	rs133042175	$2.82 X 10^{-8}$	dominant		0.02
8	66,863,804	rs136859732	$2.08 X 10^{-12}$<br>$2.08 X 10^{-12}$	additive<br>dominant		0.02<br>0.02
8	67,682,317	rs109114609	$1.47 X 10^{-9}$<br>$1.79 X 10^{-10}$	additive<br>dominant		0.02<br>0.02
8	72,863,847	rs133191466	$1.83 X 10^{-9}$	dominant		0.02
8	79,684,843	rs132830182	$3.79 X 10^{-13}$<br>$3.79 X 10^{-13}$	additive<br>dominant	NTRK2ǂ	0.03<br>0.03
8	97,074,254	rs42788579	$2.21 X 10^{-9}$	dominant	FKTNǂ	0.02
8	108,601,170	rs43580717	$2.42 X 10^{-9}$	dominant		0.02
9	5,919,500	rs133149639*	$7.03 X 10^{-11}$	dominant		0.02
9	6,866,406	rs136100571	$1.50 X 10^{-9}$	dominant		0.02

9	9,963,570	rs133966433	7.75 X 10^{-13}<br>7.75 X 10^{-13}	additive<br>dominant	SDHAF4	0.03<br>0.03
9	13,446,584	rs136206718	4.32 X 10^{-10}<br>1.55 X 10^{-14}	additive<br>dominant	CD109‡	0.02<br>0.03
9	35,028,074	rs133188284	2.10 X 10^{-16}<br>2.10 X 10^{-16}	additive<br>dominant	FRK NT5DC1	0.03<br>0.03
9	44,226,465	rs133609612	6.18 X 10^{-9}	dominant	LOC104972981‡	0.02
9	44,384,391	rs110770076	3.63 X 10^{-9}	dominant	PRDM1	0.02
9	45,148,825	rs41609220	4.89 X 10^{-33}<br>4.89 X 10^{-33}	additive<br>dominant		0.07<br>0.07
9	52,338,179	rs110821231	2.96 X 10^{-9}	additive	TRNAY-GUA	0.02
9	52,348,239	rs110294260	2.94 X 10^{-12}<br>4.66 X 10^{-10}	additive<br>dominant	TRNAY-GUA	0.02<br>0.02
9	52,350,552	rs109542104	1.27 X 10^{-8}	additive	TRNAY-GUA	0.02
9	52,351,567	rs109662298	1.47 X 10^{-8}	additive	TRNAY-GUA	0.02
9	52,352,425	rs136330197	1.27 X 10^{-8}	additive	TRNAY-GUA	0.02
9	52,353,122	rs133125776	1.61 X 10^{-8}	additive	TRNAY-GUA	0.02
9	52,354,187	rs110831114	5.16 X 10^{-12}<br>7.39 X 10^{-10}	additive<br>dominant	TRNAY-GUA	0.02<br>0.02
9	52,360,536	rs110029213	1.27 X 10^{-8}	additive	TRNAY-GUA	0.02
9	60,713,665	rs43601819	3.76 X 10^{-8}	dominant		0.02
9	65,306,072	rs43602336	4.06 X 10^{-9}<br>1.92 X 10^{-8}	additive<br>dominant	LOC101907349‡	0.02<br>0.02
9	72,452,687	rs135308951	2.81 X 10^{-10}<br>1.85 X 10^{-9}	additive<br>dominant	EYA4‡	0.02<br>0.02
9	83,077,381	rs41664363	1.72 X 10^{-17}<br>1.72 X 10^{-17}	additive<br>dominant	UTRN‡	0.04<br>0.04
9	90,375,296	rs43608400	1.38 X 10^{-8}<br>5.45 X 10^{-13}	additive<br>dominant	SYNE1‡	0.02<br>0.03
9	91,451,664	rs109821677*	1.16 X 10^{-9}	dominant		0.02
9	92,428,092	rs133498424*	8.25 X 10^{-9}	dominant		0.02
9	98,344,328	rs41588208	3.08 X 10^{-8}	dominant	AGPAT4‡	0.02
9	100,434,182	rs109080389	8.24 X 10^{-14}<br>8.24 X 10^{-14}	additive<br>dominant	QKI‡	0.03<br>0.03
9	101,345,498	rs136913747	2.43 X 10^{-11}	dominant		0.02
9	102,010,624	rs136546097	2.53 X 10^{-9}	dominant	PDE10A‡	0.02
10	1,832,559	rs134719504*	1.15 X 10^{-8}	dominant		0.02

10	3,261,109	rs42963736	2.56 X 10^{-10}	dominant	KCNN2‡	0.02
10	7,344,600	rs134147845	1.57 X 10^{-9}	dominant	SV2C‡	0.02
10	15,063,352	rs133889389	9.45 X 10^{-10}	additive	FEM1B<br>LOC104973054‡<br>TRNAC-GCA	0.02
10	20,784,448	rs134679432	1.72 X 10^{-8}<br>1.92 X 10^{-9}	additive<br>dominant	CHMP4A‡<br>GMPR2 IPO4<br>MDP1 NEDD8<br>TM9SF1 TSSK4	0.02<br>0.02
10	29,865,159	rs110325782	9.85 X 10^{-9}	dominant	FMN1‡	0.02
10	38,402,225	rs134456240*	4.33 X 10^{-11}	dominant	UBR1‡	0.02
10	57,343,779	rs137655014	1.23 X 10^{-10}	dominant		0.02
10	59,882,200	rs110617366*	7.74 X 10^{-10}<br>5.12 X 10^{-12}	additive<br>dominant	TRPM7‡	0.02<br>0.02
10	63,194,703	rs135485894	6.58 X 10^{-9}	dominant		0.02
10	63,217,046	rs133388647	2.90 X 10^{-12}<br>6.98 X 10^{-20}	additive<br>dominant		0.02<br>0.04
10	77,116,010	rs133464670	8.68 X 10^{-11}	dominant	LOC104973208	0.02
10	92,088,852	rs137187232	1.01 X 10^{-15}<br>2.89 X 10^{-15}	additive<br>dominant		0.015<br>0.015
10	93,250,721	rs109703777	3.70 X 10^{-8}	dominant	CEP128‡	0.02
10	104,219,388	rs41653547	7.63 X 10^{-10}	dominant	C10H15orf43<br>LOC104973278<br>TRIM69	0.02
11	2,125,437	rs133812771	6.00 X 10^{-9}	dominant	FAHD2A‡	0.02
11	11,691,665	rs134158654	1.60 X 10^{-8}	dominant	EXOC6B‡	0.02
11	21,211,676	rs43669974	1.24 X 10^{-11}	dominant	DHX57‡	0.02
11	30,340,497	rs133968736	2.24 X 10^{-8}	dominant		0.02
11	35,881,102	rs109807092	6.37 X 10^{-9}	dominant		0.02
11	40,920,900	rs134495076	4.12 X 10^{-9}	dominant		0.02
11	50,339,491	rs110204195*	1.36 X 10^{-8}	dominant		0.02
11	51,483,571	rs109310019	7.43 X 10^{-11}<br>1.32 X 10^{-9}	additive<br>dominant		0.02<br>0.02
11	58,149,253	rs136444067	4.49 X 10^{-8}	dominant		0.01
11	64,957,407	rs137130860	1.20 X 10^{-16}<br>1.20 X 10^{-16}	additive<br>dominant		0.03<br>0.03
11	76,549,622	rs110937755	3.08 X 10^{-9}	dominant	LOC107132946‡	0.02

11	81,885,400	rs133374668	2.74 X 10^{-9}<br>8.59 X 10^{-11}	additive<br>dominant		0.02<br>0.02
11	83,038,073	rs137538390	3.88 X 10^{-11}	dominant	NBAS[ǂ]	0.02
11	86,400,864	rs110541854	1.11 X 10^{-8}	dominant		0.02
11	91,325,669	rs133468684	3.98 X 10^{-8}	dominant		0.01
11	94,120,538	rs134709354	7.59 X 10^{-9}	dominant	RABGAP1[ǂ]	0.02
11	96,474,351	rs110930462	2.04 X 10^{-8}	dominant	MAPKAP1[ǂ]	0.02
11	101,115,391	rs136026124	8.53 X 10^{-9}	dominant	ABL1[ǂ]	0.02
12	4,302,535	rs135803901*	4.36 X 10^{-9}	dominant		0.02
12	14,220,951	rs42421894	1.92 X 10^{-9}	dominant		0.02
12	17,136,529	rs136450224	1.22 X 10^{-8}	dominant		0.02
12	19,384,047	rs133433961	2.37 X 10^{-8}<br>6.01 X 10^{-15}	additive<br>dominant	KPNA3[ǂ]<br>LOC783060	0.02<br>0.03
12	21,798,093	rs110859101	2.80 X 10^{-8}	dominant	LOC516736<br>SLC25A15	0.02
12	22,621,092	rs136870702	3.69 X 10^{-8}	dominant		0.02
12	36,866,277	rs133031236	3.48 X 10^{-8}	additive	LOC100295315<br>RNF17[ǂ]	0.02
12	43,939,457	rs133296292	6.90 X 10^{-13}<br>6.90 X 10^{-13}	additive<br>dominant		0.03<br>0.03
12	44,369,482	rs136399659	4.20 X 10^{-9}	dominant	KLHL1[ǂ]	0.02
12	58,518,230	rs110718934	2.68 X 10^{-8}	additive		0.02
12	63,506,888	rs42401835*	1.31 X 10^{-8}<br>1.31 X 10^{-8}	additive<br>dominant		0.02<br>0.02
12	82,705,374	rs109399990	9.93 X 10^{-9}	dominant	FGF14[ǂ]	0.02
12	88,309,027	rs135467227	1.33 X 10^{-8}<br>8.61 X 10^{-9}	additive<br>dominant	MYO16[ǂ]	0.02<br>0.02
13	1,247,948	rs43711088	2.51 X 10^{-17}<br>2.51 X 10^{-17}	additive<br>dominant	PLCB1[ǂ]	0.04<br>0.04
13	8,920,866	rs41679483	8.63 X 10^{-10}	dominant	MACROD2[ǂ]	0.02
13	31,920,795	rs109574513	3.79 X 10^{-8}	dominant	TRDMT1[ǂ]	0.02
13	32,879,609	rs109617842	1.12 X 10^{-9}<br>1.27 X 10^{-12}	additive<br>dominant	CACNB2[ǂ]	0.02<br>0.02
13	42,137,191	rs41687892	2.56 X 10^{-8}	dominant	TRNAS-GGA	0.02
13	45,484,656	rs42628484	2.19 X 10^{-8}<br>1.34 X 10^{-8}	additive<br>dominant	PFKP RNF17<br>PITRM1[ǂ]	0.02<br>0.02
14	12,011,754	rs109257200	2.02 X 10^{-8}	recessive		0.02

14	14,183,625	rs135029808	1.12×10^{-8}	dominant		0.02
14	20,508,616	rs136484948	6.23×10^{-9}	dominant		0.02
14	23,508,165	rs41730395	1.18×10^{-12}	additive	ATP6V1H	0.02
			1.18×10^{-12}	dominant		0.02
14	25,425,357	rs41722033	1.74×10^{-11}	dominant	LOC101907667	0.02
14	25,633,578	rs134826452	1.67×10^{-21}	additive		0.04
			1.67×10^{-21}	dominant		0.04
14	28,634,983	rs135570111	9.25×10^{-10}	dominant	CLVS1‡	0.02
14	41,600,948	rs137036103*	7.29×10^{-9}	dominant		0.02
14	43,099,166	rs42856301	3.77×10^{-12}	additive		0.02
			3.77×10^{-12}	dominant		0.02
14	43,953,144	rs134492410	3.13×10^{-9}	dominant	PKIA‡	0.02
14	45,667,262	rs136545426	3.09×10^{-8}	dominant	LOC100138499	0.02
14	50,291,072	rs41913814	2.64×10^{-11}	recessive		0.02
14	60,686,566	rs137829593	1.89×10^{-15}	additive		0.03
			1.89×10^{-15}	dominant		0.03
15	2,135,130	rs136034166	2.24×10^{-8}	additive	GRIA4‡	0.02
			2.49×10^{-13}	dominant		0.03
15	5,208,201	rs41662040	8.93×10^{-10}	additive		0.02
			1.63×10^{-11}	dominant		0.02
15	31,040,001	rs133729246	7.26×10^{-9}	additive		0.02
			7.26×10^{-9}	dominant		0.02
15	54,722,255	rs41770954	1.03×10^{-12}	dominant	POLD3‡	0.03
15	55,552,388	rs137402563	8.10×10^{-14}	additive	MAP6‡	0.03
			8.10×10^{-14}	dominant		0.03
15	61,669,862	rs109416226	5.81×10^{-10}	additive		0.02
			5.71×10^{-10}	dominant		0.02
15	63,247,583	rs135885524	3.15×10^{-8}	dominant	ELP4‡	0.02
15	67,825,769	rs133312284	2.86×10^{-9}	dominant	RAG1‡	0.02
15	74,287,401	rs133748740	3.82×10^{-8}	dominant	API5	0.02
15	84,195,776	rs29021878	2.73×10^{-10}	additive	LOC101907407	0.02
			8.54×10^{-11}	dominant	PATL1	0.02
16	13,537,834	rs137170596	1.82×10^{-8}	additive	RGS21‡	0.02
			9.94×10^{-10}	dominant		0.02
16	15,473,841	rs135148389	1.09×10^{-8}	additive		0.02
			1.09×10^{-8}	dominant		0.02

16	15,481,171	rs134711585	4.86×10^{-11}<br>1.46×10^{-12}	additive<br>dominant		0.02<br>0.02
16	20,500,260	rs110701354	1.53×10^{-16}<br>1.53×10^{-16}	additive<br>dominant	USH2A‡	0.03<br>0.03
16	35,683,918	rs134810606	1.83×10^{-8}	dominant	BECN2<br>LOC104974409<br>LOC104974410	0.02
16	40,004,888	rs41804723	7.62×10^{-9}<br>2.45×10^{-10}	additive<br>dominant	VAMP4‡	0.02<br>0.02
16	60,351,587	rs109008645	9.99×10^{-11}	dominant	LOC104974482‡<br>TRNAG-CCC	0.02
16	65,396,450	rs133026423	1.85×10^{-9}	dominant	DHX9‡	0.02
16	67,766,937	rs42465725	4.70×10^{-12}	dominant	IVNS1ABP‡	0.02
16	69,808,920	rs42378772	1.64×10^{-9}<br>2.28×10^{-11}	additive<br>dominant		0.02<br>0.02
17	4,548,256	rs110253313	5.01×10^{-9}<br>1.63×10^{-10}	additive<br>dominant		0.02<br>0.02
17	7,238,905	rs42503264	5.47×10^{-10}<br>1.64×10^{-13}	additive<br>dominant	LRBA‡<br>MAB21L2	0.02<br>0.03
17	14,147,211	rs135563413*	4.10×10^{-8}<br>2.01×10^{-12}	additive<br>dominant	TRNAG-UCC	0.01<br>0.02
17	40,345,468	rs110378748	2.09×10^{-11}<br>7.20×10^{-15}	additive<br>dominant		0.02<br>0.03
17	41,089,654	rs110704136	9.09×10^{-9}<br>1.46×10^{-9}	additive<br>dominant	FNIP2‡	0.02<br>0.02
17	47,203,601	rs41844776	3.63×10^{-9}	dominant	ADGRD1<br>TRNAW-CCA	0.02
17	54,321,728	rs136973422	4.37×10^{-8}	dominant	DDX55‡	0.01
17	55,237,847	rs136539859	3.56×10^{-10}<br>3.56×10^{-10}	additive<br>dominant	CLIP1	0.02<br>0.02
17	57,905,713	rs41846781	2.90×10^{-11}<br>6.67×10^{-15}	additive<br>dominant	CIT‡<br>LOC104974648	0.02<br>0.03
17	57,934,917	rs137751476	1.05×10^{-8}	dominant	CIT‡	0.02
17	62,614,798	rs110449993	3.17×10^{-10}<br>3.17×10^{-10}	additive<br>dominant		0.02<br>0.02
17	64,006,908	rs134002184	2.43×10^{-8}	dominant	PTPN11	0.02
18	5,461,878	rs109191862	1.71×10^{-8}	dominant	WWOX‡	0.02

18	21,080,949	rs132676005	3.15×10^{-9}	dominant	TOX3ǂ	0.02
18	21,805,397	rs135055053*	4.08×10^{-9}	additive	CHD9ǂ	0.02
18	21,929,721	rs41872094	4.18×10^{-9}<br>1.27×10^{-11}	additive<br>dominant	AKTIPǂ RBL2	0.02<br>0.02
18	55,406,165	rs41888920	1.34×10^{-10}<br>1.99×10^{-9}	additive<br>dominant	BOSTAUV1R403<br>BOSTAUV1R404<br>LOC100847353	0.02<br>0.02
18	65,112,331	rs133139781*	1.60×10^{-11}	dominant	LOC790271ǂ	0.02
19	8,114,894	rs109147231	2.30×10^{-8}<br>1.32×10^{-10}	additive<br>dominant		0.02<br>0.02
19	12,537,367	rs133524472	2.68×10^{-9}	additive		0.023
19	24,125,771	rs137270020	4.16×10^{-18}<br>4.16×10^{-18}	additive<br>dominant	LOC100336161<br>PAFAH1B1ǂ	0.04<br>0.04
19	36,438,190	rs109963616*	1.15×10^{-8}	dominant	SPAG9	0.02
19	47,475,942	rs41917870	2.14×10^{-9}	recessive	EFCAB3ǂ	0.02
19	48,613,006	rs41921835	4.54×10^{-9}	dominant	MAP3K3ǂ	0.02
19	56,555,839	rs137804276	1.06×10^{-8}	dominant	RECQL5ǂ	0.02
19	58,347,709	rs109193093	1.56×10^{-8}<br>1.56×10^{-8}	additive<br>dominant	SDK2ǂ	0.02<br>0.02
20	11,320,893	rs133688167	2.20×10^{-8}<br>9.47×10^{-11}	additive<br>dominant	PIK3R1	0.02<br>0.02
20	42,618,265	rs137766956*	8.11×10^{-9}	dominant		0.02
20	46,054,426	rs41949865*	1.76×10^{-8}	dominant		0.02
20	56,531,652	rs135191929	6.65×10^{-10}	dominant	MYO10ǂ	0.02
20	57,113,135	rs41956232	7.87×10^{-10}<br>3.34×10^{-12}	additive<br>dominant	11-marǂ	0.02<br>0.02
20	57,773,680	rs135111737	2.71×10^{-8}	dominant	FBXL7ǂ	0.02
20	60,649,127	rs42536899	2.27×10^{-8}	dominant		0.02
20	66,434,683	rs41960759	2.87×10^{-9}	dominant		0.02
20	67,303,443	rs41977686	2.45×10^{-9}<br>2.27×10^{-10}	additive<br>dominant		0.02<br>0.02
21	9,179,282	rs135625205	9.58×10^{-9}	dominant		0.02
21	14,800,548	rs41619364	1.38×10^{-8}	dominant	LOC785523	0.02
21	19,191,643	rs41968656*	7.95×10^{-9}	dominant		0.02
21	21,479,311	rs133287618	3.19×10^{-8}	additive	KIF7<br>TICRR	0.02

21	22,547,601	rs133488994	5.33 X 10^{-9}	dominant	IQGAP1‡	0.02
21	40,692,579	rs110658904	6.82 X 10^{-9}	dominant	PRKD1‡	0.02
21	53,293,514	rs133762978	6.89 X 10^{-9}	dominant		0.02
21	67,397,173	rs109800519	1.04 X 10^{-9}<br>3.31 X 10^{-12}	additive<br>dominant	MEG3	0.02<br>0.02
22	6,613,617	rs134241191	4.48 X 10^{-8}	dominant	TRNAG-CCC	0.01
22	7,883,649	rs134321851	1.42 X 10^{-9}<br>4.20 X 10^{-13}	additive<br>dominant	CLASP2‡	0.02<br>0.03
22	9,972,687	rs42578220	1.74 X 10^{-11}<br>1.33 X 10^{-11}	additive<br>dominant		0.02<br>0.02
22	28,846,011	rs136708885	1.45 X 10^{-9}<br>2.47 X 10^{-13}	additive<br>dominant	GXYLT2‡	0.02<br>0.03
22	38,612,397	rs109985339	2.28 X 10^{-10}	dominant		0.02
22	38,679,433	rs109836331	3.45 X 10^{-8}	dominant	CADPS	0.02
22	44,912,994	rs42011047	4.81 X 10^{-11}<br>6.11 X 10^{-9}	additive<br>dominant	FAM208A‡	0.02<br>0.02
22	48,223,561	rs41624710	8.50 X 10^{-10}	dominant	DCP1A‡	0.02
23	23,965,902	rs41634508	4.77 X 10^{-21}<br>4.77 X 10^{-21}	additive<br>dominant	PKHD1‡	0.04<br>0.04
23	28,746,821	rs134213841	1.56 X 10^{-9}	recessive	LOC101906691<br>LOC107131735<br>MOG‡ ZFP57	0.02
23	34,332,434	rs133368886	4.23 X 10^{-19}<br>1.97 X 10^{-17}	additive<br>dominant	LOC780995	0.04<br>0.04
23	34,453,204	rs110105721*	3.60 X 10^{-8}<br>1.34 X 10^{-9}	additive<br>dominant	PRP8	0.02<br>0.02
23	34,505,837	rs133953512*	4.25 X 10^{-8}	dominant	PRP6	0.01
23	40,664,436	rs133879598	3.81 X 10^{-9}	dominant	GMPR‡	0.02
24	6,039,397	rs43146766*	1.18 X 10^{-8}<br>4.43 X 10^{-10}	additive<br>dominant		0.02<br>0.02
24	15,172,605	rs109757717	7.89 X 10^{-9}	dominant		0.02
24	19,100,872	rs110602508	2.37 X 10^{-11}	dominant		0.02
24	23,442,843	rs110264722	1.69 X 10^{-9}	dominant	NOL4‡	0.02
24	36,648,988	rs109498992	6.71 X 10^{-21}<br>6.71 X 10^{-21}	additive<br>dominant		0.04<br>0.04
24	37,585,202	rs136377593	3.20 X 10^{-8}	dominant	LPIN2‡	0.02

24	46,912,208	rs109156282	9.22×10^{-10}<br>6.90×10^{-12}	additive<br>dominant	ST8SIA5‡	0.02<br>0.02
24	49,798,018	rs109472635	1.32×10^{-10}	dominant		0.02
24	50,503,943	rs110770205	1.38×10^{-8}	recessive		0.02
24	61,573,806	rs133176874	2.87×10^{-11}	dominant	PHLPP1‡	0.02
24	62,451,691	rs133737149	1.08×10^{-11}	dominant	LOC511106<br>LOC519132<br>LOC786410	0.02
25	13,523,513	rs134250904*	6.15×10^{-11}<br>1.28×10^{-12}	additive<br>dominant	PARN‡	0.02<br>0.02
25	34,478,989	rs135016010	4.06×10^{-10}	dominant	HIP1‡	0.02
25	37,064,123	rs134623086	1.22×10^{-8}	dominant	LOC101904303‡	0.02
25	39,846,521	rs109120989	6.45×10^{-10}	dominant	FOXK1<br>LOC101907846<br>MIR2890	0.02
25	40,432,330	rs134578134	2.83×10^{-8}<br>1.93×10^{-8}	additive<br>dominant	SDK1‡	0.02<br>0.02
26	7,709,388	rs133146678	3.29×10^{-10}	dominant	PRKG1‡	0.02
26	13,829,714	rs42293525	1.85×10^{-8}<br>1.19×10^{-8}	additive<br>dominant	5-Mar	0.02<br>0.02
26	20,714,265	rs42086190	3.41×10^{-9}	dominant	DNMBP‡	0.02
26	25,589,629	rs110088444	5.16×10^{-14}<br>2.34×10^{-14}	additive<br>dominant	SORCS3‡	0.03<br>0.03
26	31,743,621	rs110951772	7.91×10^{-12}<br>1.72×10^{-12}	additive<br>dominant	BBIP1 MIR4680<br>MIR6524<br>PDCD4‡ SHOC2	0.02<br>0.02
26	33,257,060	rs42095933*	2.81×10^{-10}	dominant	VTI1A‡	0.02
26	41,354,357	rs136057362	4.36×10^{-8}	dominant	LOC101907208‡	0.01
27	7,688,299	rs42439113	8.25×10^{-10}	dominant	AGA<br>LOC101904157	0.02
27	18,991,970	rs109928360	5.84×10^{-10}<br>3.54×10^{-10}	additive<br>dominant	LOC107131905<br>MTMR7‡<br>TRNAE-UUC	0.02<br>0.02
27	21,083,026	rs110750240	7.00×10^{-11}	dominant		0.02
27	21,375,791	rs132728892	1.84×10^{-35}<br>1.84×10^{-35}	additive<br>dominant		0.07<br>0.07
28	6,330,810	rs135488223	4.19×10^{-11}	dominant		0.02

28	22,005,009	rs110596728*	4.56×10^{-10}	dominant		0.02
28	26,832,602	rs133727040	4.80×10^{-11}<br>8.41×10^{-13}	additive<br>recessive		0.02<br>0.03
28	27,785,987	rs109367560	1.31×10^{-9}	dominant	CDH23ǂ	0.02
28	29,116,183	rs136830696	4.74×10^{-8}	dominant	PLA2G12Bǂ	0.01
28	29,592,323	rs136467650	6.87×10^{-9}<br>6.87×10^{-9}	additive<br>dominant	ANXA7<br>MSS51ǂ	0.02<br>0.02
28	30,040,605	rs135182095	4.05×10^{-8}	dominant	VCL	0.01
28	31,582,482	rs109038837	4.64×10^{-8}	additive		0.01
28	34,346,919	rs110585405	4.28×10^{-11}	dominant		0.02
29	25,819,074	rs110160296	3.42×10^{-9}<br>1.01×10^{-10}	additive<br>dominant		0.02<br>0.02
29	27,820,784	rs43170197*	1.32×10^{-8}	dominant	LOC787694ǂ	0.02
29	31,640,269	rs42708672	3.96×10^{-8}	additive		0.01
29	44,722,284	rs42188587	4.33×10^{-9}<br>3.46×10^{-10}	additive<br>dominant	LOC104976284<br>SART1<br>TSGA10IPǂ	0.02<br>0.02
X	10,845,015	rs110621747	2.78×10^{-9}<br>3.46×10^{-10}	additive<br>dominant		0.02<br>0.02
X	21,807,318	rs109220334	9.94×10^{-9}	dominant		0.02
X	21,827,945	rs110658504	2.72×10^{-10}	dominant	MAGEA11	0.02
X	36,569,364	rs136840493*	3.49×10^{-8}	additive	KIR3DL1<br>LOC107131295ǂ	0.02
X	36,574,505	rs135593222*	1.24×10^{-9}<br>9.02×10^{-11}	additive<br>dominant	KIR3DL1ǂ<br>LOC107131295	0.02<br>0.02
X	45,175,649	rs134819091	2.20×10^{-16}<br>2.20×10^{-16}	additive<br>dominant		0.03<br>0.03
X	62,588,913	rs137706243	3.54×10^{-9}	dominant		0.02
X	65,081,915	rs133502506	7.50×10^{-9}	dominant	LOC100294935	0.02
X	86,160,589	rs133939924	1.33×10^{-12}<br>1.33×10^{-12}	additive<br>dominant	FAM155B<br>LOC104970534	0.02<br>0.02
X	101,652,487	rs135383970	2.57×10^{-9}	dominant	ARHGEF9ǂ	0.02
X	128,845,516	rs110577907	6.78×10^{-9}<br>3.68×10^{-10}	additive<br>dominant		0.02<br>0.02
X	147,800,877	rs136023557	2.56×10^{-8}	dominant	NLGN4Xǂ	0.02

[1]Chromosome location of the locus

[2]SNP location as measured by numbered nucleotides in reference to the UMD 3.1 genome assembly (http://bovinegenome.org/?q=node/61)

[3]The most significant SNP in the locus associated with cow conception rate as identified by *rs* number which is a reference number assigned to markers submitted to the National Center for Biotechnology Information SNP database (https://www.ncbi.nlm.nih.gov/projects/SNP/)

[4]SNP located within previously identified copy number variations (CNVs) are denoted with an *.

[5]Significance (*P*-value) of the most significant SNP associated with cow conception rate.

[6]Genome-wide association model.

[7]Positional candidate gene(s) located within +/- 19 kb from associated SNP(s)

ǂ Genes with SNPs identified within their coding sequence

[8]Contribution of each SNP to the total variance

Supplementary Table 2. Loci Associated with Number of Times Bred to Achieve Pregnancy

BTA[1]	UMD 3.1 Position (bp)[2]	SNP ID[3,4]	Models[5]	p-Values[6]	Positional Candidate Genes[7]	Proportion of variance explained[8]
1	3,546,098	*rs136767715*	additive<br>dominant	1.52×10^{-8}<br>7.89×10^{-13}	*TIAM1*ǂ	0.02<br>0.02
1	7,862,063	*rs135020930*	additive<br>dominant	2.62×10^{-13}<br>2.62×10^{-13}		0.016<br>0.016
1	11,250,683	*rs110647268**	additive<br>dominant	5.88×10^{-16}<br>5.88×10^{-16}		0.018<br>0.022
1	11,812,309	*rs109447046*	additive<br>dominant	2.37×10^{-9}<br>1.70×10^{-13}		0.027<br>0.019
1	13,923,360	*rs134204993**	dominant	4.53×10^{-8}		0.016
1	26,755,718	*rs133334228*	dominant	1.89×10^{-8}	*ROBO1*ǂ	0.018
1	29,618,772	*rs43224096*	additive<br>dominant	4.21×10^{-15}<br>4.60×10^{-17}		0.017<br>0.029
1	32,700,496	*rs43226666**	additive<br>dominant	3.60×10^{-9}<br>2.50×10^{-13}	*CADM2*ǂ	0.016<br>0.023
1	36,710,870	*rs136512158*	dominant	2.06×10^{-9}	*EPHA3*	0.018
1	36,925,522	*rs136668233**	dominant	4.07×10^{-11}	*EPHA3*ǂ	0.018
1	45,719,982	*rs43682172*	additive<br>dominant	2.87×10^{-8}<br>1.14×10^{-12}	*ABI3BP*ǂ	0.022<br>0.025
1	45,909,194	*rs110537421*	dominant	3.94×10^{-8}		0.018
1	52,895,536	*rs109491771**	dominant	2.09×10^{-9}		0.016
1	56,582,506	*rs110092138*	dominant	2.53×10^{-8}		0.022<br>0.017
1	62,056,267	*rs42760220*	dominant	9.99×10^{-12}		0.019
1	62,158,499	*rs42675527*	additive<br>dominant	2.22×10^{-13}<br>8.03×10^{-19}	*LOC538060*	0.021<br>0.021
1	64,591,504	*rs43652271*	additive<br>dominant	2.76×10^{-13}<br>1.87×10^{-15}	*UPK1B*ǂ	0.019<br>0.019
1	64,894,430	*rs42911131*	additive<br>dominant	4.02×10^{-9}<br>1.31×10^{-13}	*CD80*ǂ<br>*TIMMDC1*	0.023<br>0.023
1	65,061,560	*rs43234278*	dominant	1.40×10^{-8}	*MAATS1*ǂ	0.017

1	84,709,807	*rs109101339**	dominant	2.86×10^{-8}	*ATP11B*<br>*DCUN1D1*	0.023<br>0.02
1	89,279,321	*rs109891698*	dominant	1.49×10^{-8}		0.022
1	94,300,486	*rs41606324**	additive<br>dominant	3.97×10^{-25}<br>2.01×10^{-25}	*NLGN1*‡	0.017<br>0.017
1	94,998,936	*rs135160436*	additive<br>dominant	3.73×10^{-8}<br>5.42×10^{-11}	*SPATA16*‡	0.019<br>0.027
1	99,584,226	*rs110848318*	additive<br>dominant	1.41×10^{-9}<br>3.04×10^{-17}		0.017<br>0.017
1	108,986,133	*rs42282809*	dominant	3.53×10^{-9}		0.016
1	112,961,101	*rs110435444*	additive<br>dominant	1.27×10^{-11}<br>8.70×10^{-16}	*PLCH1*‡	0.02<br>0.02
1	117,359,373	*rs134846486*	additive<br>dominant	8.31×10^{-9}<br>3.75×10^{-13}		0.022<br>0.024
1	117,571,948	*rs133089690*	dominant	1.10×10^{-9}	*MED12L*‡	0.041
1	118,817,882	*rs43260026*	dominant	8.09×10^{-10}	*LOC1049710*<br>*06*	0.017
1	123,956,245	*rs132851673*	dominant	5.14×10^{-11}		0.04
1	126,235,693	*rs136458590**	additive<br>dominant	1.38×10^{-10}<br>3.46×10^{-14}	*SLC9A9*‡	0.016<br>0.022
1	129,836,154	*rs136894301*	dominant	6.08×10^{-9}	*CLSTN2*‡	0.017
1	130,576,374	*rs110960328*	additive<br>dominant	7.24×10^{-10}<br>2.32×10^{-13}	*KPNA6*‡<br>*LOC1049710*<br>*35*‡ *RBP1*	0.027<br>0.027
1	139,950,787	*rs43278206*	dominant	2.36×10^{-9}	*MRPL3*	0.016
1	154,853,461	*rs134813046*	additive<br>dominant	8.74×10^{-9}<br>1.05×10^{-11}		0.017<br>0.017
1	157,576,622	*rs136888926**	dominant	1.08×10^{-8}		0.017
1	158,010,340	*rs43289085*	additive<br>dominant	1.21×10^{-12}<br>2.18×10^{-13}		0.017<br>0.024
2	6,611,760	*rs109028820*	dominant	2.28×10^{-8}	*ASNSD1*‡	0.016
2	10,440,119	*rs110576003*	dominant	5.26×10^{-10}		0.024
2	11,744,112	*rs133600796*	additive<br>dominant	1.69×10^{-9}<br>3.93×10^{-10}	*ZNF804A*‡	0.021 0.033

2	15,775,602	*rs136049910*	additive	1.98×10^{-9}	*TRNASTOP-UCA*<br>*UBE2E3*	0.016
2	26,960,403	*rs137188435*	dominant	3.85×10^{-8}	*LRP2*‡	0.018
2	32,783,586	*rs135787342*	dominant	2.77×10^{-10}	*FIGN*‡	0.021
2	36,425,765	*rs136627254*	additive<br>dominant	1.24×10^{-8}<br>1.09×10^{-10}	*PLA2R1*‡	0.054<br>0.054
2	39,686,326	*rs134790315*	dominant	1.24×10^{-9}		0.017
2	55,050,085	*rs42416884*	dominant	1.72×10^{-8}		0.025
2	55,236,847	*rs111005641*	additive	4.50×10^{-8}		0.023
2	58,258,750	*rs42354740*	additive	1.35×10^{-8}		0.018
2	65,451,280	*rs133761371*	additive	1.02×10^{-8}		0.02
2	65,493,191	*rs109322207*	dominant	3.14×10^{-9}		0.016
2	73,212,371	*rs137104904*	dominant	2.70×10^{-8}		0.03
2	79,240,905	*rs109507969*	dominant	1.77×10^{-9}	*GYPC*	0.021
2	80,429,623	*rs134254979*	dominant	3.94×10^{-14}	*LOC104971267*‡	0.022
2	84,127,565	*rs110922881**	dominant	2.82×10^{-8}		0.022
2	85,462,609	*rs109342415*	dominant	4.28×10^{-10}	*LOC531691*‡	0.018
2	85,872,885	*rs134179168*	additive<br>dominant	6.17×10^{-13}<br>7.72×10^{-19}	*PGAP1*‡	0.016<br>0.022
2	87,872,337	*rs41616585**	additive<br>dominant	2.47×10^{-8}<br>1.37×10^{-10}		0.03<br>0.032
2	88,137,677	*rs135769112**	additive<br>dominant	2.20×10^{-15}<br>2.20×10^{-15}		0.022<br>0.024
2	88,201,219	*rs110322233*	dominant	3.96×10^{-8}		0.032
2	94,405,242	*rs109065091*	dominant	1.30×10^{-11}	*PARD3B*	0.017
2	98,167,527	*rs41641857*	dominant	4.02×10^{-9}	*UNC80*‡	0.017
2	103,031,098	*rs135057060*	dominant	9.08×10^{-9}	*VWC2L*‡	0.017
2	126,326,843	*rs132632350*	additive<br>dominant	1.58×10^{-16}<br>4.89×10^{-14}	*AHDC1*	0.018<br>0.016
2	134,379,046	*rs109722054*	dominant	2.93×10^{-8}	*ALDH4A1*‡	0.021
3	2,328,452	*rs137454379*	dominant	2.80×10^{-8}		0.017
3	7,783,286	*rs135239306*	additive<br>dominant	1.48×10^{-10}<br>4.92×10^{-8}	*ATF6*‡	0.018<br>0.024
3	12,500,599	*rs133108396*	additive<br>dominant	1.58×10^{-10}<br>2.14×10^{-10}	*LOC531264*	0.016<br>0.022

3	13,676,438	*rs135949924*	dominant	3.44×10^{-8}	*TRNAS-GGA*	0.016<br>0.023
3	14,665,964	*rs136725222*	dominant	3.00×10^{-10}	*SEMA4A*ǂ	0.019
3	32,269,604	*rs43336503*	dominant	6.43×10^{-12}	*CHI3L2*ǂ	0.025
3	35,996,083	*rs111017447*	dominant	4.12×10^{-11}	*NTNG1*ǂ	0.016
3	38,557,710	*rs109265034*	dominant	1.98×10^{-10}		0.018
3	39,163,108	*rs134279953*	additive	2.82×10^{-10}		0.034
3	41,684,925	*rs135507420*	recessive	5.47×10^{-11}		0.016
3	42,093,978	*rs134200102*	dominant	1.89×10^{-9}		0.019
3	48,737,120	*rs133995663*	additive	1.01×10^{-8}		0.016
3	49,686,970	*rs136708725*	additive<br>dominant	1.02×10^{-9}<br>6.53×10^{-13}	*ABCA4*<br>*LOC783590*	0.02
3	50,041,196	*rs137661970*	additive<br>dominant	4.03×10^{-9}<br>2.94×10^{-9}	*BCAR3*ǂ<br>*LOC101905185*	0.017
3	50,121,744	*rs135149460*	additive<br>dominant	3.74×10^{-10}<br>1.59×10^{-9}	*FNBP1L*ǂ	0.023<br>0.027
3	56,967,816	*rs137196969*	dominant	1.11×10^{-8}		0.016
3	60,726,800	*rs43342616*	dominant	4.99×10^{-10}		0.018
3	66,777,217	*rs133073532*	additive<br>dominant	7.68×10^{-9}<br>4.44×10^{-13}	*GIPC2*<br>*LOC100140103*	0.018<br>0.024
3	67,152,286	*rs41659269*	recessive	1.95×10^{-8}	*FAM73A*<br>*USP33*	0.02<br>0.026
3	74,585,417	*rs42228228*	dominant	4.40×10^{-9}	*PTGER3*ǂ	0.034
3	77,163,315	*rs136533238*	additive<br>dominant	3.61×10^{-11}<br>1.68×10^{-14}	*LOC101907544 RPE65*ǂ	0.016
3	84,157,548	*rs137498683*	dominant	1.69×10^{-11}	*INADL*ǂ	0.018
3	97,680,312	*rs41585055*	additive<br>dominant	1.22×10^{-8}<br>9.07×10^{-13}	*AGBL4*ǂ	0.018<br>0.019
3	98,096,750	*rs29024665*	dominant	1.70×10^{-8}	*AGBL4*<br>*BEND5*	0.018
3	98,855,342	*rs43356386*	dominant	2.37×10^{-12}	*LOC101906301*<br>*TRABD2B*	0.016
3	101,247,684	*rs43361651*	additive<br>dominant	5.81×10^{-9}<br>6.24×10^{-11}	*LOC107132349 TESK2*ǂ	0.016<br>0.016

3	115,692,498	*rs109046293**	dominant	5.97×10^{-10}		0.02<br>0.02
3	116,328,811	*rs133498551*	additive<br>dominant	1.14×10^{-9}<br>1.11×10^{-11}	*ASB18*ǂ	0.017
3	119,504,166	*rs41615294*	dominant	3.17×10^{-9}	*LOC1003364<br>76*	0.022
4	5,954,962	*rs137299312*	additive<br>dominant	2.15×10^{-19}<br>2.15×10^{-19}	*VWC2*ǂ	0.016<br>0.03
4	6,150,669	*rs136754108**	additive	2.00×10^{-8}		0.017
4	6,371,195	*rs133733598*	dominant	2.27×10^{-11}		0.023
4	6,502,615	*rs137736954*	dominant	3.89×10^{-8}		0.023
4	17,088,259	*rs133291985*	dominant	9.43×10^{-11}	*GLCCI1<br>LOC1071323<br>81*	0.016
4	17,611,774	*rs41659165*	dominant	4.93×10^{-11}	*NXPH1*ǂ	0.018
4	18,435,856	*rs43376515*	dominant	3.31×10^{-8}		0.021 0.036
4	24,223,070	*rs43385175*	dominant	2.30×10^{-9}	*LOC1002949<br>09*	0.027 0.028
4	24,761,362	*rs137563859*	dominant	2.15×10^{-10}	*ISPD*ǂ	0.019 0.022
4	25,579,450	*rs42345007*	dominant	2.42×10^{-8}	*LOC781774*	0.023
4	29,794,883	*rs43181427*	dominant	4.15×10^{-9}		0.017
4	34,132,924	*rs41592806*	additive<br>dominant	3.18×10^{-8}<br>2.93×10^{-10}		0.016
4	37,235,676	*rs133417267*	additive<br>dominant	4.99×10^{-15}<br>4.99×10^{-15}	*SEMA3E*ǂ	0.022
4	51,437,631	*rs109335929*	dominant	1.68×10^{-8}	*WNT2*ǂ	0.018
4	54,918,207	*rs42421993*	additive<br>dominant	2.65×10^{-12}<br>2.96×10^{-13}	*PPP1R3A*	0.017
4	66,232,207	*rs108957740*	dominant	2.76×10^{-9}	*NOD1*ǂ<br>*GGCT*	0.021
4	78,821,054	*rs133821212*	additive<br>dominant	2.37×10^{-8}<br>8.50×10^{-11}	*C4H7orf25<br>MRPL32*ǂ<br>*PSMA2*	0.022
4	80,320,092	*rs43407832*	dominant	4.85×10^{-9}		0.019
4	85,958,373	*rs135340912*	dominant	3.65×10^{-8}	*KCND2*ǂ	0.017

4	93,277,289	*rs109721084*	additive<br>dominant	6.58×10^{-11}<br>3.42×10^{-14}	*LEP*	0.02
4	94,023,459	*rs133540884*	dominant	8.16×10^{-9}	*AHCYL2*ǂ	0.017 0.023
4	94,120,438	*rs133223938*	additive<br>dominant	4.83×10^{-8}<br>7.70×10^{-12}	*AHCYL2*ǂ<br>*LOC783260*	0.017 0.017
4	97,756,608	*rs110356330*	additive<br>dominant	1.19×10^{-8}<br>2.14×10^{-8}	*LOC787799*ǂ	0.015 0.024
4	98,336,209	*rs135524891*	dominant	3.48×10^{-8}	*EXOC4*ǂ	0.017
4	100,040,158	*rs133842860*	dominant	9.65×10^{-10}	*CNOT4*ǂ	0.017
4	102,090,604	*rs133499092*	dominant	4.90×10^{-8}	*DGKI*ǂ	0.017 0.027
4	109,038,482	*rs132930684*	additive<br>dominant	9.62×10^{-10}<br>2.17×10^{-11}		0.025 0.025
4	110,200,691	*rs110149102*	dominant	2.72×10^{-10}		0.019
4	111,213,416	*rs136229180*	dominant	4.97×10^{-8}	*CNTNAP2*ǂ	0.017 0.016
4	114,052,951	*rs43686023**	dominant	3.15×10^{-9}	*GIMAP6*<br>*LOC768255*	0.016
4	116,534,717	*rs133516143*	additive<br>dominant	1.87×10^{-8}<br>2.97×10^{-10}		0.016
4	117,606,629	*rs137620917*	additive<br>dominant	4.67×10^{-59}<br>4.67×10^{-59}	*DPP6*ǂ	0.055 0.055
4	118,148,549	*rs133872172*	additive<br>dominant	2.74×10^{-9}<br>5.09×10^{-11}	*RBM33*ǂ	0.016
4	118,420,186	*rs136974031*	additive<br>dominant	7.49×10^{-12}<br>7.49×10^{-12}		0.018
4	119,201,399	*rs43420944*	dominant	8.21×10^{-11}		0.016
5	6,290,970	*rs132725080**	dominant	3.88×10^{-8}	*CSRP2*<br>*LOC1049723*<br>*27*<br>*LOC1049723*<br>*29*	0.016
5	13,208,260	*rs135881737*	recessive	4.62×10^{-9}	*LOC1049723*<br>*47*ǂ	0.016
5	16,995,758	*rs136714577*	dominant	6.16×10^{-10}		0.017 0.021
5	17,173,888	*rs134628502*	additive<br>dominant	2.56×10^{-9}<br>5.72×10^{-15}		0.036 0.036
5	29,350,321	*rs110713926*	additive<br>dominant	6.69×10^{-10}<br>5.25×10^{-10}	*ATF1*<br>*DIP2B*ǂ	0.016 0.016

5	33,240,014	*rs135651218*	additive<br>dominant	8.49 X 10^{-11}<br>1.56 X 10^{-18}	*PCED1B*[ǂ]	0.02
5	37,947,429	*rs133375087*	additive<br>dominant	1.23 X 10^{-10}<br>6.69 X 10^{-13}		0.021
5	40,576,651	*rs43434026*	recessive	3.03 X 10^{-10}	*MUC19*[ǂ]	0.016
5	44,853,575	*rs134622788*	dominant	3.60 X 10^{-12}		0.048 0.048
5	46,675,202	*rs136875517*	dominant	2.06 X 10^{-8}		0.019
5	46,946,293	*rs134326497*	dominant	3.96 X 10^{-9}	*LOC1003374<br>78*[ǂ]	0.021
5	47,230,538	*rs133350338*	dominant	1.06 X 10^{-10}	*LOC1003374<br>78*[ǂ]	0.019 0.019
5	48,012,423	*rs135945780*	additive<br>dominant	2.15 X 10^{-9}<br>2.15 X 10^{-9}		0.019
5	51,059,426	*rs29022233*	dominant	3.98 X 10^{-8}	*PPM1H*[ǂ]	0.016
5	69,086,128	*rs135161024*	dominant	3.51 X 10^{-8}	*APPL2*[ǂ]	0.022
5	72,465,721	*rs109476686*	dominant	1.52 X 10^{-8}	*LARGE*[ǂ]	0.027
5	72,903,919	*rs132740823*	additive<br>dominant	9.72 X 10^{-11}<br>1.10 X 10^{-10}		0.018
5	76,167,701	*rs109300805**	additive<br>dominant	1.94 X 10^{-14}<br>6.24 X 10^{-17}	*ELFN2*	0.017
5	76,599,679	*rs43054854**	dominant	3.19 X 10^{-8}		0.018
5	77,199,853	*rs43439753**	additive<br>dominant	2.65 X 10^{-12}<br>2.80 X 10^{-18}		0.016
5	88,633,335	*rs137120693*	dominant	3.85 X 10^{-8}		0.021
5	94,325,298	*rs135948685*	additive<br>dominant	9.47 X 10^{-9}<br>6.51 X 10^{-12}	*DERA*[ǂ]	0.022
5	100,303,435	*rs108959142*	additive<br>dominant	7.81 X 10^{-11}<br>1.02 X 10^{-10}	*CLEC1A<br>CLEC7A*	0.023
5	107,407,965	*rs133201447*	additive<br>dominant	2.54 X 10^{-8}<br>2.15 X 10^{-12}	*FOXM1<br>ITFG2<br>LOC1049725<br>69<br>LOC782076<br>NRIP2*	0.018 0.027
5	111,790,193	*rs109912186*	dominant	2.73 X 10^{-13}	*GRAP2*[ǂ]	0.028

5	119,989,587	*rs133819714*	additive<br>dominant	2.05 X 10^{-9}<br>4.59 X 10^{-9}	*ADM2 LOC101903087 LOC104972615 MIOX SBF1*‡	0.017 0.027
5	120,478,584	*rs109532733*	dominant	8.66 X 10^{-11}		0.02 0.028
6	11,154,927	*rs43452214*	additive<br>dominant	6.85 X 10^{-15}<br>9.34 X 10^{-15}		0.019 0.021
6	11,301,952	*rs29019244*	dominant	6.16 X 10^{-9}		0.018 0.022
6	11,640,946	*rs108963104**	dominant	1.59 X 10^{-16}		0.016
6	13,154,909	*rs43450481*	dominant	3.73 X 10^{-10}	*CAMK2D*‡	0.019
6	18,311,190	*rs42489971*	dominant	8.45 X 10^{-9}	*LOC107132549*‡	0.016 0.019
6	28,624,189	*rs133675848*	dominant	6.73 X 10^{-9}	*LOC782977*	0.017
6	32,399,521	*rs110488959*	dominant	1.00 X 10^{-9}	*LOC536367*‡	0.027 0.028
6	42,265,935	*rs110203806*	dominant	3.83 X 10^{-10}	*KCNIP4*‡	0.017
6	42,942,951	*rs134604999*	additive	3.45 X 10^{-8}	*KCNIP4*‡	0.029 0.029
6	44,529,753	*rs132958625*	dominant	5.29 X 10^{-11}	*LOC104972733*‡	0.017
6	48,243,519	*rs134948194*	dominant	1.31 X 10^{-8}		0.016
6	61,601,562	*rs109303139*	dominant	3.25 X 10^{-8}	*APBB2*‡	0.016 0.022
6	68,709,723	*rs135139162*	additive<br>dominant	2.92 X 10^{-19}<br>3.76 X 10^{-19}	*SLAIN2*‡	0.016
6	73,503,493	*rs109467082*	additive<br>dominant	6.85 X 10^{-19}<br>6.85 X 10^{-19}	*PAICS*‡<br>*PPAT*<br>*SRP72*	0.018
6	73,853,804	*rs43468439*	dominant	1.10 X 10^{-11}	*REST*‡	0.017
6	74,910,779	*rs110648584*	additive<br>dominant	6.05 X 10^{-9}<br>2.14 X 10^{-9}		0.016 0.031
6	78,372,811	*rs136660262*	additive<br>dominant	2.75 X 10^{-8}<br>3.96 X 10^{-14}	*ADGRL3*	0.02 0.021
6	81,047,108	*rs43125352**	dominant	1.11 X 10^{-11}		0.022
6	85,034,144	*rs29004040*	additive<br>dominant	2.58 X 10^{-8}<br>1.59 X 10^{-10}	*STAP1*‡<br>*UBA6*	0.015 0.025
6	88,153,418	*rs43473246*	dominant	5.70 X 10^{-9}		0.016 0.016

6	92,423,076	*rs42553812*	dominant	3.20×10^{-8}		0.024
6	92,587,809	*rs110063753*	additive<br>dominant	1.71×10^{-10}<br>1.43×10^{-14}	*CXCL9*<br>*SDAD1*	0.017
6	93,263,835	*rs43476570*	additive<br>dominant	2.45×10^{-9}<br>2.64×10^{-13}	*SHROOM3*ǂ	0.03<br>0.036
6	97,543,454	*rs135934271*	additive	5.12×10^{-9}		0.022 0.022
6	102,407,680	*rs134284115*	dominant	1.80×10^{-8}	*ARHGAP24*ǂ	0.016
6	107,302,731	*rs42919834**	dominant	1.11×10^{-8}	*LOC1019021*<br>*06 NSG1*<br>*RGS12*	0.015 0.024
6	107,617,673	*rs137693158**	additive<br>dominant	2.15×10^{-9}<br>4.98×10^{-8}	*LOC1019084*<br>*03*ǂ	0.015
6	114,510,383	*rs109630077*	additive<br>dominant	1.57×10^{-10}<br>2.81×10^{-15}		0.018
6	117,439,794	*rs110817413*	dominant	6.56×10^{-12}		0.021<br>0.03
6	118,987,625	*rs135802025*	additive<br>dominant	1.33×10^{-8}<br>8.24×10^{-14}	*AFAP1*ǂ	0.018
7	6,118,322	*rs132731242*	additive<br>dominant	3.48×10^{-8}<br>2.55×10^{-11}	*F2RL3*<br>*SIN3B*ǂ	0.017 0.027
7	12,213,252	*rs137569961*	additive<br>dominant	3.66×10^{-8}<br>3.19×10^{-10}	*ADGRE3*ǂ<br>*LOC1001387*<br>*23*	0.016 0.021
7	13,024,481	*rs133465587*	dominant	9.61×10^{-10}		0.016
7	13,721,441	*rs41588240*	additive<br>dominant	2.83×10^{-10}<br>9.78×10^{-17}	*CALR*<br>*GADD45*<br>*GIP1*ǂ<br>*RAD23A*<br>*TRNAG-*<br>*CCC*	0.024
7	15,034,214	*rs110743590*	additive<br>dominant	8.77×10^{-10}<br>3.95×10^{-11}	*LOC509510*<br>*LOC530825*<br>*LOC787383*	0.016
7	16,070,246	*rs134710953*	dominant	1.04×10^{-9}	*FDX1L*ǂ	0.022
7	19,341,834	*rs110943465*	additive<br>dominant	2.50×10^{-10}<br>3.17×10^{-9}	*ACER1*ǂ	0.019
7	20,209,433	*rs109079807*	dominant	2.67×10^{-10}	*PTPRS*ǂ	0.028 0.041

7	28,375,798	*rs43734065*	additive<br>dominant	5.04 X 10^{-10}<br>4.84 X 10^{-10}	*43527*ǂ<br>*LOC104969129*ǂ	0.016
7	32,634,417	*rs132887259*	dominant	1.04 X 10^{-10}		0.022
7	52,052,097	*rs109062580**	additive	1.55 X 10^{-8}	*SIL1*ǂ	0.021
7	54,413,989	*rs137316807*	dominant	6.30 X 10^{-11}		0.019
7	57,914,820	*rs42282884*	dominant	4.64 X 10^{-11}		0.022
7	58,055,640	*rs133170783*	additive<br>dominant	1.08 X 10^{-8}<br>1.77 X 10^{-11}		0.017
7	62,311,969	*rs134893796*	dominant	9.64 X 10^{-9}		0.016
7	62,587,443	*rs134479919*	dominant	3.26 X 10^{-8}	*ABLIM3*ǂ	0.026
7	63,352,489	*rs43518018*	additive	1.20 X 10^{-8}	*HMGXB3*ǂ	0.017
7	64,472,010	*rs43519903*	dominant	9.46 X 10^{-10}	*CCDC69*	0.017
7	66,177,154	*rs134217351*	dominant	9.02 X 10^{-9}		0.025 0.028
7	66,660,279	*rs42810268*	dominant	2.40 X 10^{-8}		0.019
7	67,341,433	*rs109136546*	dominant	5.01 X 10^{-10}	*MFAP3*	0.017
7	69,881,920	*rs135934765*	dominant	2.45 X 10^{-11}	*SGCD*ǂ	0.016
7	75,618,692	*rs43524262*	dominant	2.96 X 10^{-8}		0.02
7	75,637,375	*rs43524270*	dominant	3.34 X 10^{-9}		0.026 0.032
7	75,775,268	*rs43530606*	dominant	1.02 X 10^{-10}	*GABRA1*	0.019
7	93,415,439	*rs136096285*	dominant	9.09 X 10^{-10}	*LOC104972872*ǂ	0.023<br>0.03
7	96,163,665	*rs109171007*	dominant	2.44 X 10^{-8}	*KIAA0825*ǂ	0.019
7	98,571,597	*rs133343732*	dominant	2.67 X 10^{-9}	*CAST*<br>*ERAP1*<br>*LOC104968992*	0.017
7	98,828,347	*rs137216406*	dominant	1.44 X 10^{-9}	*LNPEP*ǂ	0.019
7	103,930,228	*rs133117241*	dominant	7.54 X 10^{-11}	*SLCO6A1*ǂ	0.019 0.015
7	106,968,546	*rs43531491*	dominant	2.50 X 10^{-8}		0.02
8	1,285,069	*rs136213429*	dominant	4.54 X 10^{-8}	*NEK1*ǂ	0.071 0.071
8	2,353,560	*rs110390603*	dominant	1.72 X 10^{-8}		0.021
8	12,163,981	*rs136342892*	additive<br>dominant	6.35 X 10^{-9}<br>4.75 X 10^{-11}		0.018
8	19,092,309	*rs136161638**	additive<br>dominant	6.23 X 10^{-9}<br>5.91 X 10^{-14}		0.018
8	22,202,557	*rs110186355*	dominant	4.21 X 10^{-8}		0.016

8	27,874,312	*rs136472729*	dominant	2.91×10^{-9}	*BNC2*ǂ	0.017
8	28,583,167	*rs133541436*	dominant	1.85×10^{-8}	*CCDC171*ǂ	0.016
8	36,504,354	*rs137050402*	dominant	4.38×10^{-11}	*PTPRD*ǂ	0.025
8	37,065,088	*rs133042175*	dominant	1.56×10^{-10}		0.016
8	38,204,495	*rs134355106*	dominant	3.05×10^{-10}	*KDM4C*ǂ	0.022
8	45,624,366	*rs109293388*	dominant	1.59×10^{-8}	*TJP2*ǂ	0.016 0.02
8	54,054,285	*rs135532670*	dominant	1.22×10^{-8}	*GNAQ*ǂ	0.017
8	60,640,805	*rs134066757*	dominant	5.02×10^{-9}	*LOC100299372* *LOC523083* *OR2S2*	0.028
8	66,863,804	*rs136859732*	additive dominant	4.56×10^{-12} 4.56×10^{-12}		0.016
8	67,682,317	*rs109114609*	additive dominant	6.85×10^{-11} 6.55×10^{-12}		0.018
8	71,877,783	*rs134492186*	dominant	1.25×10^{-9}	*STC1*	0.023 0.036
8	72,863,847	*rs133191466*	dominant	5.78×10^{-11}		0.016 0.03
8	79,684,843	*rs132830182*	additive dominant	1.05×10^{-10} 1.05×10^{-10}	*NTRK2*ǂ	0.023
8	84,307,178	*rs43137599*	dominant	2.96×10^{-8}		0.016 0.017
8	97,074,254	*rs42788579*	dominant	4.36×10^{-11}	*FKTN*ǂ	0.022 0.03
8	104,626,077	*rs133232898*	additive dominant	4.89×10^{-10} 6.72×10^{-10}	*LOC101907033*	0.018
8	108,601,170	*rs43580717*	dominant	3.57×10^{-10}		0.035 0.029
9	5,919,500	*rs133149639**	additive dominant	3.56×10^{-8} 2.20×10^{-10}		0.017
9	6,866,406	*rs136100571*	dominant	1.76×10^{-11}		0.025
9	8,680,967	*rs109198379*	dominant	4.72×10^{-8}	*LOC788115*	0.024 0.026
9	8,815,237	*rs110478223*	dominant	2.52×10^{-10}	*LMBRD1*	0.021 0.031
9	9,963,570	*rs133966433*	additive dominant	4.51×10^{-17} 4.51×10^{-17}	*SDHAF4*	0.02 0.023
9	13,446,584	*rs136206718*	additive dominant	2.55×10^{-12} 4.61×10^{-19}	*CD109*ǂ	0.017
9	28,592,450	*rs109636996*	dominant	1.48×10^{-9}		0.019

9	32,002,498	*rs110127058**	recessive	4.07 X 10-8		0.018
9	35,028,074	*rs133188284*	additive<br>dominant	6.94 X 10-14<br>6.94 X 10-14	*FRK*<br>*NT5DC1*	0.017
9	44,226,465	*rs133609612*	dominant	1.08 X 10-8	*LOC1049729*<br>*81*ǂ	0.02<br>0.031
9	44,384,391	*rs110770076*	dominant	1.07 X 10-11	*PRDM1*	0.017 0.017
9	45,148,825	*rs41609220*	additive<br>dominant	5.72 X 10-38<br>5.72 X 10-38		0.017 0.016
9	48,483,554	*rs133444736*	dominant	1.99 X 10-9	*GRIK2*ǂ	0.023
9	60,146,602	*rs43597897*	dominant	1.05 X 10-8		0.022
9	60,713,665	*rs43601819*	additive<br>dominant	4.84 X 10-8<br>8.12 X 10-12		0.021
9	65,306,072	*rs43602336*	additive<br>dominant	1.42 X 10-10<br>1.33 X 10-9	*LOC1019073*<br>*49*ǂ	0.02<br>0.026
9	72,452,687	*rs135308951*	additive<br>dominant	8.51 X 10-9<br>3.61 X 10-8	*EYA4*ǂ	0.024 0.024
9	83,077,381	*rs41664363*	additive<br>dominant	2.31 X 10-18<br>2.31 X 10-18	*UTRN*ǂ	0.016
9	89,347,416	*rs110318317*	dominant	4.05 X 10-8		0.017
9	90,375,296	*rs43608400*	additive<br>dominant	1.18 X 10-8<br>5.66 X 10-14	*SYNE1*ǂ	0.016
9	91,451,664	*rs109821677**	dominant	2.51 X 10-10		0.02
9	92,428,092	*rs133498424**	additive<br>dominant	3.97 X 10-8<br>4.61 X 10-9		0.018
9	97,903,543	*rs133825925*	dominant	5.44 X 10-9	*SLC22A3*	0.016
9	98,344,328	*rs41588208*	additive<br>dominant	1.70 X 10-8<br>4.71 X 10-12	*AGPAT4*ǂ	0.021 0.025
9	99,208,923	*rs110927414*	dominant	4.92 X 10-8	*PARK2*ǂ	0.021<br>0.02
9	100,434,182	*rs109080389*	additive<br>dominant	2.80 X 10-13<br>2.80 X 10-13	*QKI*ǂ	0.016
9	101,083,868	*rs137284328*	dominant	1.88 X 10-9		0.017
9	101,345,498	*rs136913747*	additive<br>dominant	4.06 X 10-11<br>8.26 X 10-16		0.021
9	102,010,624	*rs136546097*	dominant	4.10 X 10-12	*PDE10A*ǂ	0.019
9	103,818,595	*rs137728083*	dominant	6.27 X 10-9		0.017
10	1,832,559	*rs134719504**	dominant	9.38 X 10-10		0.017

10	3,261,109	*rs42963736*	additive<br>dominant	6.25×10^{-10}<br>2.51×10^{-13}	*KCNN2*ǂ	0.028 0.028
10	7,193,076	*rs110381341*	dominant	4.54×10^{-8}		0.016
10	7,344,600	*rs134147845*	dominant	3.78×10^{-12}	*SV2C*ǂ	0.016 0.032
10	15,063,352	*rs133889389*	additive<br>dominant	4.41×10^{-13}<br>9.06×10^{-10}	*FEM1B LOC104973054*ǂ *TRNAC-GCA*	0.017
10	20,331,045	*rs133786459*	dominant	2.78×10^{-9}	*CD276*ǂ	0.016
10	20,784,448	*rs134679432*	additive<br>dominant	1.32×10^{-9}<br>7.83×10^{-11}	*CHMP4A*ǂ<br>*GMPR2*<br>*IPO4*<br>*MDP1*<br>*NEDD8*<br>*TM9SF1*<br>*TSSK4*	0.023 0.023
10	29,865,159	*rs110325782*	dominant	1.53×10^{-10}	*FMN1*ǂ	0.018 0.018
10	34,832,325	*rs137554479*	dominant	5.31×10^{-11}		0.017 0.021
10	38,402,225	*rs134456240**	additive<br>dominant	1.25×10^{-9}<br>8.34×10^{-15}	*UBR1*ǂ	0.023
10	57,343,779	*rs137655014*	dominant	7.20×10^{-13}		0.024
10	59,882,200	*rs110617366**	additive<br>dominant	5.40×10^{-12}<br>3.65×10^{-15}	*TRPM7*ǂ	0.017
10	63,194,703	*rs135485894*	additive<br>dominant	1.69×10^{-10}<br>4.37×10^{-12}		0.017
10	63,217,046	*rs133388647*	additive<br>dominant	3.12×10^{-11}<br>1.36×10^{-19}		0.019
10	63,689,249	*rs133765760*	dominant	5.75×10^{-9}		0.019
10	75,419,514	*rs136140522*	dominant	9.71×10^{-9}	*KCNH5*ǂ	0.018 0.018
10	76,220,343	*rs41656312*	dominant	7.45×10^{-9}	*LOC101904356*	0.023 0.042
10	77,116,010	*rs133464670*	additive<br>dominant	6.76×10^{-9}<br>6.87×10^{-14}	*LOC104973208*	0.024
10	82,364,565	*rs132734439*	dominant	1.86×10^{-9}	*SYNJ2BP*ǂ	0.016
10	84,291,187	*rs135397847*	additive	2.33×10^{-8}	*RGS6*ǂ *TRNAE-UUC*	0.018

10	92,088,852	*rs137187232*	additive dominant	4.50×10^{-8} 1.61×10^{-8}		0.016
10	93,250,721	*rs109703777*	dominant	7.11×10^{-11}	*CEP128*ǂ	0.025
10	97,330,853	*rs133139647*	dominant	2.18×10^{-8}		0.022
10	104,219,388	*rs41653547*	additive dominant	2.67×10^{-9} 3.27×10^{-13}	*C10H15orf43 LOC104973278 TRIM69*	0.024 0.024
11	2,125,437	*rs133812771*	dominant	7.87×10^{-12}	*FAHD2A*ǂ	0.016
11	6,714,691	*rs43653712**	dominant	1.69×10^{-8}	*IL1R2*	0.023
11	11,691,665	*rs134158654*	dominant	2.66×10^{-8}	*EXOC6B*ǂ	0.025
11	17,366,963	*rs41836414*	additive dominant	6.51×10^{-10} 1.14×10^{-8}		0.021 0.015
11	17,721,099	*rs134981474*	dominant	9.51×10^{-9}		0.021
11	21,211,676	*rs43669974*	dominant	9.20×10^{-12}	*DHX57*ǂ	0.019
11	30,340,497	*rs133968736*	dominant	9.33×10^{-9}		0.02 0.017
11	33,792,560	*rs136691291**	recessive	1.07×10^{-8}		0.015
11	35,881,102	*rs109807092*	additive dominant	2.34×10^{-12} 1.24×10^{-11}		0.016
11	40,920,900	*rs134495076*	additive dominant	8.82×10^{-10} 9.15×10^{-13}		0.018
11	48,862,934	*rs135578400*	additive dominant	7.86×10^{-9} 1.01×10^{-8}	*ST3GAL5*ǂ *TRNAE-UUC*	0.02 0.027
11	50,428,456	*rs43681043**	additive dominant	3.91×10^{-10} 2.04×10^{-10}	*LOC101903367 SUCLG1*ǂ	0.025
11	51,483,571	*rs109310019*	additive dominant	1.30×10^{-10} 3.65×10^{-10}		0.017
11	52,289,973	*rs136569977*	dominant	3.28×10^{-9}		0.018
11	57,058,516	*rs42234541*	dominant	8.54×10^{-11}		0.016 0.019
11	58,149,253	*rs136444067*	dominant	1.29×10^{-9}		0.02 0.02
11	60,357,999	*rs42706937*	dominant	3.73×10^{-8}	*FAM161A*ǂ	0.016 0.019
11	64,957,407	*rs137130860*	additive dominant	3.66×10^{-17} 3.66×10^{-17}		0.018
11	71,299,883	*rs110053143*	dominant	1.67×10^{-9}		0.021

11	71,918,597	*rs137231497*	additive dominant	4.57 X 10^{-8} 9.82 X 10^{-9}	*MRPL33 RBKS*ǂ	0.017
11	73,375,063	*rs134964414*	additive dominant	1.05 X 10^{-9} 7.79 X 10^{-10}	*LOC104973424*ǂ *RAB10*ǂ *TRNAC-ACA*	0.018
11	81,885,400	*rs133374668*	additive dominant	6.50 X 10^{-11} 6.54 X 10^{-12}		0.017
11	83,038,073	*rs137538390*	additive dominant	1.21 X 10^{-8} 1.07 X 10^{-13}	*NBAS*ǂ	0.018
11	86,400,864	*rs110541854*	dominant	1.27 X 10^{-10}		0.022
11	89,501,669	*rs135806178*	dominant	2.05 X 10^{-8}		0.017 0.029
11	91,325,669	*rs133468684*	dominant	7.55 X 10^{-9}		0.027 0.04
11	94,120,538	*rs134709354*	dominant	1.43 X 10^{-8}	*RABGAP1*ǂ	0.024
11	96,067,855	*rs136776805*	additive dominant	2.14 X 10^{-8} 3.35 X 10^{-8}	*PPP6C*ǂ	0.016 0.016
11	96,474,351	*rs110930462*	dominant	8.45 X 10^{-9}	*MAPKAP1*ǂ	0.019
11	101,115,391	*rs136026124*	dominant	6.99 X 10^{-9}	*ABL1*ǂ	0.019 0.023
12	4,302,535	*rs135803901**	additive dominant	4.84 X 10^{-11} 3.20 X 10^{-13}		0.019
12	14,220,951	*rs42421894*	dominant	4.87 X 10^{-11}		0.019 0.03
12	16,306,649	*rs110474118*	dominant	2.00 X 10^{-9}	*LCP1*ǂ	0.016
12	17,136,529	*rs136450224*	dominant	2.08 X 10^{-8}		0.016
12	19,384,047	*rs133433961*	additive dominant	4.68 X 10^{-13} 2.28 X 10^{-20}	*KPNA3*ǂ *LOC783060*	0.02
12	21,400,380	*rs133119471*	dominant	2.23 X 10^{-8}	*ATP7B CCDC70*	0.016
12	21,798,093	*rs110859101*	dominant	1.67 X 10^{-10}	*LOC516736 SLC25A15*	0.02 0.017
12	22,621,092	*rs136870702*	dominant	7.52 X 10^{-11}		0.024
12	43,799,317	*rs29017133*	dominant	2.95 X 10^{-8}		0.017 0.017
12	43,939,457	*rs133296292*	additive dominant	1.29 X 10^{-11} 1.29 X 10^{-11}		0.023 0.023
12	44,369,482	*rs136399659*	dominant	7.41 X 10^{-11}	*KLHL1*ǂ	0.028 0.033
12	48,647,199	*rs135959058*	recessive	6.62 X 10^{-9}		0.018

12	58,518,230	*rs110718934*	additive	$4.90 X 10^{-9}$		0.023
12	61,404,223	*rs136725238*	dominant	$1.91 X 10^{-8}$		0.016
12	81,780,656	*rs135307240*	dominant	$4.82 X 10^{-8}$	*NALCN*ǂ	0.021 0.031
12	82,705,374	*rs109399990*	dominant	$8.33 X 10^{-9}$	*FGF14*ǂ	0.017
12	82,890,429	*rs110783012*	dominant	$2.51 X 10^{-8}$	*TPP2*ǂ	0.017 0.026
13	1,247,948	*rs43711088*	additive<br>dominant	$2.28 X 10^{-16}$<br>$2.28 X 10^{-16}$	*PLCB1*ǂ	0.018
13	2,264,173	*rs41676079*	dominant	$4.20 X 10^{-8}$	*PLCB4*ǂ	0.017
13	8,920,866	*rs41679483*	dominant	$1.64 X 10^{-9}$	*MACROD2*ǂ	0.032 0.036
13	19,311,965	*rs137544619*	dominant	$4.73 X 10^{-9}$	*PARD3*ǂ	0.018
13	30,062,520	*rs109947626*	dominant	$1.87 X 10^{-8}$	*FAM171A1*	0.018 0.028
13	31,920,795	*rs109574513*	dominant	$1.81 X 10^{-9}$	*TRDMT1*ǂ	0.026
13	32,879,609	*rs109617842*	additive<br>dominant	$5.54 X 10^{-12}$<br>$1.81 X 10^{-16}$	*CACNB2*ǂ	0.019
13	35,842,851	*rs135323330*	dominant	$1.74 X 10^{-8}$		0.031 0.031
13	42,137,191	*rs41687892*	dominant	$9.65 X 10^{-9}$	*TRNAS-GGA*	0.017
13	45,484,656	*rs42628484*	additive<br>dominant	$4.54 X 10^{-8}$<br>$3.89 X 10^{-8}$	*PFKP*<br>*PITRM1*ǂ	0.02<br>0.019
13	46,208,034	*rs41699216*	dominant	$1.46 X 10^{-8}$		0.018 0.032
13	57,799,152	*rs41566209*	dominant	$1.13 X 10^{-8}$		0.016
13	58,245,216	*rs137096935*	additive	$1.79 X 10^{-9}$	*STX16*ǂ	0.017 0.025
13	58,333,285	*rs132864771*	dominant	$7.57 X 10^{-12}$		0.019
14	1,514,056	*rs41630614*	dominant	$8.53 X 10^{-9}$	*COMMD5*<br>*RPL8*<br>*ZNF34*<br>*ZNF7*	0.02<br>0.028
14	2,198,215	*rs110288957*	dominant	$1.65 X 10^{-8}$	*SCRIB*ǂ	0.019
14	12,011,754	*rs109257200*	recessive	$9.80 X 10^{-12}$		0.016
14	14,183,625	*rs135029808*	dominant	$4.11 X 10^{-8}$		0.027 0.044
14	17,291,481	*rs41839430*	dominant	$3.09 X 10^{-8}$	*TMEM65*ǂ	0.02<br>0.022
14	20,508,616	*rs136484948*	dominant	$5.02 X 10^{-9}$		0.02
14	21,358,853	*rs135136778*	additive<br>dominant	$4.71 X 10^{-10}$<br>$1.11 X 10^{-10}$		0.024
14	23,508,165	*rs41730395*	additive<br>dominant	$1.71 X 10^{-11}$<br>$1.71 X 10^{-11}$	*ATP6V1H*	0.02

14	25,425,357	*rs41722033*	additive<br>dominant	6.74×10^{-10}<br>7.03×10^{-15}	*LOC101907667*	0.016 0.026
14	25,633,578	*rs134826452*	additive<br>dominant	7.83×10^{-21}<br>7.83×10^{-21}		0.017 0.017
14	28,634,983	*rs135570111*	dominant	4.89×10^{-9}	*CLVS1*ǂ	0.019
14	41,600,948	*rs137036103**	dominant	9.60×10^{-9}		0.022 0.026
14	43,099,166	*rs42856301*	additive<br>dominant	1.31×10^{-14}<br>1.31×10^{-14}		0.02
14	43,953,144	*rs134492410*	additive<br>dominant	1.72×10^{-9}<br>1.55×10^{-11}	*PKIA*ǂ	0.023 0.031
14	43,995,061	*rs134590876*	dominant	6.19×10^{-9}	*PKIA*	0.025 0.032
14	45,667,262	*rs136545426*	dominant	3.59×10^{-10}	*LOC100138499*	0.024 0.024
14	50,291,072	*rs41913814*	recessive	1.21×10^{-12}		0.016 0.017
14	60,686,566	*rs137829593*	additive<br>dominant	8.67×10^{-12}<br>8.67×10^{-12}		0.016 0.021
14	61,546,318	*rs132917548*	dominant	2.31×10^{-8}		0.02
14	70,639,444	*rs109864366*	dominant	9.25×10^{-9}		0.016 0.026
14	78,909,801	*rs134190366*	dominant	1.28×10^{-8}	*ATP6V0D2*ǂ	0.129 0.129
14	84,114,637	*rs108991276*	dominant	9.13×10^{-9}	*COL14A1*<br>*MRPL13*	0.022
14	84,342,736	*rs133125476*	dominant	4.09×10^{-9}	*SNTB1*ǂ	0.019 0.018
15	2,135,130	*rs136034166*	additive<br>dominant	1.83×10^{-9}<br>1.65×10^{-14}	*GRIA4*ǂ	0.019
15	5,208,201	*rs41662040*	additive<br>dominant	2.41×10^{-9}<br>1.07×10^{-11}		0.018
15	5,246,327	*rs133630731*	dominant	3.05×10^{-8}		0.021 0.018
15	22,458,768	*rs41596221**	dominant	6.85×10^{-9}	*ALG9*ǂ	0.021
15	25,669,397	*rs109391843*	dominant	1.56×10^{-9}		0.017
15	30,935,833	*rs41756356*	dominant	1.13×10^{-8}		0.018
15	47,135,215	*rs109396578*	additive	3.57×10^{-9}	*DNHD1*ǂ	0.032 0.032
15	49,853,609	*rs134423901**	dominant	1.99×10^{-8}	*LOC100337265*<br>*LOC100847430*<br>*LOC100847455*	0.016

15	54,722,255	*rs41770954*	dominant	2.50 X 10^{-13}	*POLD3*ǂ	0.017
15	55,552,388	*rs137402563*	additive<br>dominant	9.72 X 10^{-13}<br>9.72 X 10^{-13}	*MAP6*ǂ	0.018 0.028
15	61,669,862	*rs109416226*	additive<br>dominant	9.26 X 10^{-11}<br>6.42 X 10^{-11}		0.017
15	63,247,583	*rs135885524*	additive<br>dominant	2.61 X 10^{-10}<br>5.53 X 10^{-10}	*ELP4*ǂ	0.018 0.033
15	67,825,769	*rs133312284*	dominant	7.96 X 10^{-10}	*RAG1*ǂ	0.017 0.029
15	71,759,045	*rs134381641*	dominant	9.92 X 10^{-9}	*LRRC4C*ǂ	0.021 0.022
15	77,101,592	*rs135114146*	dominant	3.62 X 10^{-8}		0.028 0.028
15	84,195,776	*rs29021878*	additive<br>dominant	2.56 X 10^{-10}<br>1.04 X 10^{-10}	*LOC1019074<br>07 PATL1*	0.02
16	510,040	*rs41785918*	dominant	4.66 X 10^{-8}	*TMEM183A*	0.016
16	13,537,834	*rs137170596*	additive<br>dominant	1.76 X 10^{-8}<br>9.17 X 10^{-10}	*RGS21*ǂ	0.016<br>0.02
16	15,473,841	*rs135148389*	additive<br>dominant	1.90 X 10^{-8}<br>1.90 X 10^{-8}		0.034 0.034
16	15,481,171	*rs134711585*	additive<br>dominant	5.58 X 10^{-12}<br>4.36 X 10^{-13}		0.02<br>0.028
16	16,473,798	*rs133881641*	dominant	4.70 X 10^{-8}		0.016 0.021
16	20,500,260	*rs110701354*	additive<br>dominant	2.22 X 10^{-20}<br>2.22 X 10^{-20}	*USH2A*ǂ	0.021 0.021
16	22,523,720	*rs108994652*	dominant	1.74 X 10^{-8}	*TGFB2*ǂ	0.024 0.026
16	34,026,492	*rs42936399*	dominant	1.65 X 10^{-8}		0.017
16	35,683,918	*rs134810606*	dominant	4.20 X 10^{-9}	*BECN2<br>LOC1049744<br>09<br>LOC1049744<br>10*	0.018<br>0.016
16	38,054,540	*rs110343387*	dominant	8.33 X 10^{-9}	*SELP*ǂ	0.017
16	40,004,888	*rs41804723*	additive<br>dominant	1.04 X 10^{-9}<br>4.61 X 10^{-12}	*VAMP4*ǂ	0.018
16	42,464,695	*rs134524715*	recessive	1.21 X 10^{-8}	*TNFRSF8*ǂ	0.023
16	55,725,736	*rs43039941*	additive<br>dominant	5.29 X 10^{-10}<br>3.23 X 10^{-8}	*LOC1003364<br>25<br>LOC520023*	0.019

					LOC529969 *LOC789197*	
16	60,351,587	*rs109008645*	dominant	4.25×10^{-9}	*LOC104974482*ǂ	0.018
16	65,396,450	*rs133026423*	additive dominant	4.20×10^{-11} 7.89×10^{-17}	*DHX9*ǂ *LOC101905857*	0.028
16	67,766,937	*rs42465725*	additive dominant	3.70×10^{-9} 2.40×10^{-15}	*IVNS1ABP*ǂ *SWT1*	0.021 0.019
16	69,744,456	*rs42385478*	dominant	3.93×10^{-8}		0.016
16	69,808,920	*rs42378772*	additive dominant	1.97×10^{-12} 4.53×10^{-15}		0.019
16	72,801,288	*rs41823539*	dominant	2.61×10^{-8}	*ATF3* *FAM71A*	0.019
16	77,514,553	*rs136158697*	dominant	2.66×10^{-8}	*CD46*ǂ	0.026 0.028
17	4,548,256	*rs110253313*	additive dominant	6.55×10^{-9} 1.63×10^{-10}		0.022 0.027
17	4,755,665	*rs41639843*	dominant	4.53×10^{-8}	*ARFIP1*ǂ	0.017
17	7,238,905	*rs42503264*	additive dominant	3.99×10^{-11} 3.63×10^{-14}	*LRBA*ǂ *MAB21L2*	0.019
17	11,531,170	*rs110801333**	dominant	1.02×10^{-9}	*TTC29*ǂ	0.019
17	11,543,183	*rs110144635**	dominant	2.52×10^{-8}	*TTC29*ǂ	0.019
17	14,147,211	*rs135563413**	additive dominant	2.11×10^{-8} 2.73×10^{-15}	*TRNAG-UCC*	0.02
17	14,477,434	*rs110372003**	dominant	1.17×10^{-9}		0.021
17	19,532,664	*rs109506894*	dominant	3.54×10^{-8}		0.016
17	28,295,990	*rs109063837*	dominant	2.30×10^{-10}		0.016
17	31,095,739	*rs42945744*	dominant	4.63×10^{-8}		0.016 0.016
17	32,284,932	*rs110556114*	dominant	1.73×10^{-11}		0.019 0.019
17	34,336,560	*rs109571195*	dominant	2.24×10^{-8}		0.019
17	37,352,797	*rs109750304*	dominant	3.47×10^{-9}	*FSTL5*ǂ	0.019
17	40,345,468	*rs110378748*	additive dominant	2.21×10^{-14} 1.00×10^{-18}		0.021
17	41,089,654	*rs110704136*	additive dominant	2.23×10^{-11} 3.20×10^{-13}	*FNIP2*ǂ	0.017

17	44,261,883	*rs41906864**	dominant	9.65×10^{-10}		0.02 0.016
17	46,125,255	*rs110610109*	dominant	4.84×10^{-8}	*EP400*ǂ	0.02
17	47,203,601	*rs41844776*	dominant	5.65×10^{-11}	*ADGRD1 TRNAW-CCA*	0.017
17	54,321,728	*rs136973422*	dominant	1.15×10^{-8}	*DDX55*ǂ	0.018
17	55,237,847	*rs136539859*	additive dominant	3.79×10^{-11} 3.79×10^{-11}	*CLIP1*	0.018
17	57,905,713	*rs41846781*	additive dominant	2.94×10^{-11} 9.53×10^{-15}	*CIT*ǂ *LOC104974648*	0.015
17	57,934,917	*rs137751476*	dominant	2.88×10^{-9}	*CIT*ǂ	0.019
17	62,614,798	*rs110449993*	additive dominant	6.18×10^{-9} 6.18×10^{-9}		0.039 0.039
17	65,811,553	*rs41854091*	dominant	6.21×10^{-10}		0.02
17	68,230,867	*rs135956985*	recessive	3.08×10^{-8}	*SEZ6L*ǂ	0.025
18	4,244,018	*rs41861636*	dominant	1.71×10^{-8}		0.035
18	13,054,633	*rs109071329*	dominant	1.72×10^{-8}	*ZCCHC14*ǂ	0.025 0.041
18	21,080,949	*rs132676005*	dominant	3.79×10^{-9}	*TOX3*ǂ	0.016
18	21,929,721	*rs41872094*	additive dominant	3.33×10^{-10} 5.08×10^{-13}	*AKTIP*ǂ *RBL2*	0.019
18	22,133,647	*rs110817255*	dominant	8.02×10^{-10}	*FTO*ǂ	0.018 0.025
18	25,406,362	*rs135881758*	dominant	1.43×10^{-8}	*TRNAL-CAG*	0.025 0.024
18	41,341,040	*rs41874978**	dominant	2.82×10^{-11}	*ZNF536*ǂ	0.018
18	42,326,718	*rs41855620*	dominant	1.23×10^{-9}	*LOC783434*ǂ	0.017
18	48,080,342	*rs110004030*	dominant	1.89×10^{-9}	*SIPA1L3*ǂ	0.017
18	60,502,979	*rs43726266**	dominant	3.39×10^{-8}	*LOC788871*ǂ	0.017
18	62,610,581	*rs109045973*	additive dominant	4.37×10^{-10} 1.67×10^{-12}	*BRSK1 LOC104974950 TMEM150B*ǂ	0.017 0.024
18	65,112,331	*rs133139781**	additive dominant	5.09×10^{-9} 1.79×10^{-15}	*LOC104974970 LOC790271*ǂ *LOC790271*	0.018 0.022

					TRNAW-CCA	
19	7,771,626	*rs110084507*	dominant	1.73×10^{-8}		0.035 0.035
19	8,114,894	*rs109147231*	dominant	8.11×10^{-10}		0.019
19	8,755,982	*rs135740624*	additive<br>dominant	6.63×10^{-10}<br>2.76×10^{-9}		0.021
19	20,467,790	*rs135360951*	additive<br>dominant	9.28×10^{-9}<br>4.76×10^{-11}	*SLC46A1*	0.026
19	24,125,771	*rs137270020*	additive<br>dominant	3.56×10^{-16}<br>3.56×10^{-16}	*LOC100336161*<br>*PAFAH1B1*ǂ	0.021
19	36,438,190	*rs109963616**	dominant	1.62×10^{-11}	*SPAG9*	0.017 0.023
19	36,455,088	*rs41914197**	dominant	3.04×10^{-9}		0.016
19	36,603,139	*rs133431883**	dominant	7.58×10^{-11}	*LOC512899*<br>*LUC7L3*	0.022
19	36,634,826	*rs42624363**	dominant	2.43×10^{-9}	*LUC7L3*ǂ	0.025 0.039
19	36,647,418	*rs137150056**	dominant	2.75×10^{-10}	*LOC786793*ǂ	0.018
19	47,475,942	*rs41917870*	additive<br>recessive	3.30×10^{-8}<br>8.34×10^{-10}	*EFCAB3*ǂ	0.016 0.018
19	48,613,006	*rs41921835*	additive<br>dominant	3.41×10^{-12}<br>1.21×10^{-14}	*LIMD2*<br>*MAP3K3*ǂ<br>*STRADA*	0.016 0.022
19	48,761,018	*rs41923460*	additive	1.81×10^{-8}	*GH1*<br>*SMARCD2*<br>*TCAM1*	0.018
19	51,861,727	*rs41600337*	additive<br>dominant	3.40×10^{-9}<br>9.14×10^{-12}	*ACTG1*<br>*FAAP100*<br>*FSCN2*<br>*MIR3533*	0.019 0.037
19	53,443,757	*rs110291598*	dominant	6.86×10^{-9}		0.018
19	56,555,839	*rs137804276*	additive<br>dominant	4.86×10^{-8}<br>2.48×10^{-12}	*RECQL5*ǂ<br>*SAP30BP*<br>*SMIM5*<br>*SMIM6*	0.02
19	58,347,709	*rs109193093*	additive<br>dominant	1.53×10^{-8}<br>1.53×10^{-8}	*SDK2*ǂ	0.019 0.024

20	11,320,893	*rs133688167*	additive<br>dominant	4.89×10^{-10}<br>1.16×10^{-13}	*PIK3R1*	0.016
20	17,522,768	*rs135293700*	additive<br>dominant	6.32×10^{-9}<br>1.83×10^{-11}	*TRNAG-CCC*	0.031 0.031
20	20,534,357	*rs137021158*	dominant	4.03×10^{-8}	*RAB3C*ǂ	0.018
20	28,016,050	*rs137681615*	additive<br>dominant	1.81×10^{-8}<br>1.01×10^{-9}		0.02
20	28,255,221	*rs133442114*	dominant	5.87×10^{-10}		0.019
20	28,553,271	*rs136316656*	additive<br>dominant	8.92×10^{-9}<br>1.51×10^{-10}		0.044 0.044
20	32,650,164	*rs110765367*	dominant	4.02×10^{-8}		0.016
20	34,621,058	*rs41942122*	dominant	2.26×10^{-8}		0.017
20	41,389,949	*rs136889965*	dominant	2.24×10^{-8}	*MTMR12*ǂ	0.017
20	42,618,265	*rs137766956**	dominant	1.28×10^{-8}		0.024 0.026
20	51,811,579	*rs135702037**	dominant	6.38×10^{-9}		0.02 0.02
20	53,074,084	*rs135839614*	dominant	1.36×10^{-9}		0.017 0.022
20	54,923,630	*rs132897552*	dominant	2.82×10^{-8}		0.016 0.026
20	56,531,652	*rs135191929*	additive<br>dominant	1.84×10^{-8}<br>1.17×10^{-13}	*MYO10*ǂ	0.023
20	57,113,135	*rs41956232*	additive<br>dominant	6.76×10^{-10}<br>9.60×10^{-13}	*March11*ǂ	0.016 0.022
20	57,773,680	*rs135111737*	dominant	3.53×10^{-10}	*FBXL7*ǂ	0.019
20	58,444,700	*rs134221395*	additive<br>dominant	3.37×10^{-8}<br>2.79×10^{-8}	*ANKH*ǂ<br>*TRNAG-UCC*	0.016
20	60,649,127	*rs42536899*	dominant	9.40×10^{-10}		0.022
20	66,175,689	*rs110981497*	dominant	1.02×10^{-8}		0.017
20	66,434,683	*rs41960759*	dominant	4.08×10^{-9}		0.018
20	67,303,443	*rs41977686*	additive<br>dominant	1.76×10^{-9}<br>1.28×10^{-9}		0.015
21	9,179,282	*rs135625205*	additive<br>dominant	1.26×10^{-9}<br>2.21×10^{-10}		0.017
21	14,800,548	*rs41619364*	dominant	3.51×10^{-10}	*LOC785523*	0.016
21	19,191,643	*rs41968656**	dominant	3.39×10^{-10}		0.022
21	22,547,601	*rs133488994*	dominant	1.66×10^{-10}	*IQGAP1*ǂ	0.019 0.025
21	25,754,672	*rs109417706**	additive<br>dominant	3.00×10^{-9}<br>3.00×10^{-9}	*RASGRF1*ǂ	0.017

21	38,272,015	*rs133180960*	additive<br>dominant	8.67×10^{-9}<br>6.92×10^{-11}	*LOC785745*	0.019 0.024
21	40,692,579	*rs110658904*	additive<br>dominant	2.88×10^{-8}<br>2.33×10^{-12}	*PRKD1*ǂ	0.017
21	52,289,920	*rs137802601*	dominant	1.30×10^{-8}		0.028<br>0.04
21	53,293,514	*rs133762978*	dominant	3.17×10^{-10}		0.017
21	54,994,332	*rs136941405*	dominant	8.84×10^{-10}		0.017
21	65,862,391	*rs41585303*	additive	1.39×10^{-8}	*BCL11B*ǂ	0.024
21	67,397,173	*rs109800519*	additive<br>dominant	4.34×10^{-9}<br>3.48×10^{-11}	*MEG3*	0.016
21	69,347,110	*rs109181868*	dominant	6.63×10^{-9}	*CDC42BPB*	0.024
22	4,799,349	*rs110535106*	dominant	2.65×10^{-9}		0.02<br>0.018
22	6,613,617	*rs134241191*	additive<br>dominant	3.27×10^{-8}<br>6.71×10^{-10}	*TRNAG-CCC*	0.025 0.027
22	7,883,649	*rs134321851*	additive<br>dominant	2.81×10^{-10}<br>9.99×10^{-16}	*CLASP2*ǂ	0.016
22	9,972,687	*rs42578220*	additive<br>dominant	1.06×10^{-11}<br>1.13×10^{-12}		0.016
22	25,739,928	*rs42643134*	dominant	1.31×10^{-8}		0.022<br>0.04
22	27,370,812	*rs136691637*	dominant	5.84×10^{-9}	*CNTN3*ǂ	0.03<br>0.04
22	28,846,011	*rs136708885*	additive<br>dominant	1.91×10^{-13}<br>3.00×10^{-19}	*GXYLT2*ǂ	0.017
22	38,612,397	*rs109985339*	additive<br>dominant	1.73×10^{-8}<br>5.87×10^{-16}		0.025 0.031
22	38,679,433	*rs109836331*	dominant	1.76×10^{-9}	*CADPS*	0.024
22	38,788,505	*rs110891027*	additive	4.65×10^{-8}	*CADPS*ǂ	0.016
22	39,159,742	*rs135795816*	additive	8.98×10^{-9}	*PTPRG*	0.018
22	41,859,997	*rs42227841*	dominant	4.47×10^{-8}	*FHIT*ǂ	0.016
22	44,912,994	*rs42011047*	additive<br>dominant	2.08×10^{-11}<br>4.03×10^{-10}	*FAM208A*ǂ	0.016
22	46,275,925	*rs42009542*	additive<br>dominant	1.38×10^{-9}<br>1.66×10^{-9}	*LOC104975559*ǂ	0.016
22	48,223,561	*rs41624710*	dominant	2.67×10^{-11}	*DCP1A*ǂ	0.018

22	48,562,499	*rs109398421*	dominant	2.91 X 10^{-8}	*TMEM110*ǂ	0.017
22	50,701,770	*rs42019299**	additive<br>dominant	2.02 X 10^{-8}<br>1.13 X 10^{-10}	*GNAI2*<br>*SLC38A3*<br>*TRNAE-UUC*	0.024 0.033
22	60,033,741	*rs42024541**	dominant	1.09 X 10^{-8}	*DNAJB8*<br>*GATA2*<br>*LOC101906160*	0.016 0.021
22	61,292,355	*rs110574162**	dominant	6.07 X 10^{-9}	*KLF15*ǂ	0.022
23	1,171,440	*rs137313677*	dominant	4.78 X 10^{-8}		0.022
23	10,681,507	*rs110034730*	dominant	2.54 X 10^{-8}	*CPNE5*ǂ	0.02
23	11,601,873	*rs135600509*	additive<br>dominant	1.46 X 10^{-10}<br>9.30 X 10^{-15}	*MDGA1*	0.045 0.045
23	17,209,231	*rs133988650*	additive<br>dominant	3.38 X 10^{-8}<br>1.49 X 10^{-9}	*LOC101904647*	0.021
23	20,320,655	*rs109579148*	dominant	3.24 X 10^{-8}		0.018 0.031
23	23,965,902	*rs41634508*	additive<br>dominant	5.55 X 10^{-25}<br>5.55 X 10^{-25}	*PKHD1*ǂ	0.019
23	24,775,092	*rs133097021**	dominant	1.75 X 10^{-8}		0.021 0.027
23	25,442,124	*rs109992423**	dominant	4.13 X 10^{-9}	*LOC100848815*<br>*LOC100851058*	0.017 0.034
23	34,332,434	*rs133368886*	additive<br>dominant	1.21 X 10^{-8}<br>2.88 X 10^{-8}	*LOC780995*	0.017
23	34,453,204	*rs110105721**	additive<br>dominant	1.83 X 10^{-8}<br>1.41 X 10^{-10}	*PRP8*	0.019
23	34,505,837	*rs133953512**	dominant	4.02 X 10^{-10}	*PRP6*	0.016 0.028
23	40,602,185	*rs136999273*	dominant	2.82 X 10^{-9}	*ATXN1*ǂ	0.016
23	42,391,390	*rs136686460*	dominant	1.97 X 10^{-9}		0.037 0.037
23	46,827,847	*rs134836842*	dominant	3.74 X 10^{-8}		0.016
23	47,335,140	*rs109518863*	dominant	6.34 X 10^{-9}	*TXNDC5*ǂ	0.019<br>0.02

24	6,039,397	*rs43146766**	additive<br>dominant	4.23×10^{-8}<br>4.61×10^{-11}		0.016
24	6,866,479	*rs135250622**	dominant	9.56×10^{-10}	*LOC104975731*ǂ	0.02
24	15,172,605	*rs109757717*	dominant	4.24×10^{-12}		0.02
24	19,100,872	*rs110602508*	additive<br>dominant	1.13×10^{-8}<br>7.65×10^{-14}		0.017 0.022
24	23,442,843	*rs110264722*	dominant	2.23×10^{-11}	*NOL4*ǂ	0.017 0.022
24	28,498,009	*rs135696977*	dominant	4.06×10^{-8}		0.023
24	33,247,006	*rs136755241*	additive<br>dominant	8.54×10^{-9}<br>1.47×10^{-8}	*LOC104968606*ǂ	0.019 0.025
24	34,221,005	*rs135529640*	dominant	1.48×10^{-8}		0.083 0.083
24	36,648,988	*rs109498992*	additive<br>dominant	2.37 X 10-22<br>2.37 X 10-22		0.022 0.024
24	37,585,202	*rs136377593*	dominant	1.76×10^{-12}	*LPIN2*ǂ	0.016
24	42,500,735	*rs133226482*	dominant	1.62×10^{-8}		0.022 0.022
24	46,912,208	*rs109156282*	additive<br>dominant	9.37×10^{-11}<br>1.32×10^{-11}	*ST8SIA5*ǂ	0.02<br>0.026
24	49,798,018	*rs109472635*	additive<br>dominant	1.96×10^{-8}<br>1.10×10^{-14}		0.02
24	57,467,913	*rs109600414*	additive<br>dominant	2.73×10^{-10}<br>6.90×10^{-10}	*ATP8B1*ǂ	0.017
24	61,573,806	*rs133176874*	dominant	6.66×10^{-16}	*PHLPP1*ǂ	0.017
24	62,451,691	*rs133737149*	dominant	1.86×10^{-13}	*BMP6 LOC101907314 TXNDC5*	0.018 0.022
25	3,497,319	*rs133770356*	dominant	4.37×10^{-9}	*GLIS2*ǂ	0.017
25	13,523,513	*rs134250904**	additive<br>dominant	8.65×10^{-12}<br>1.14×10^{-12}	*PARN*ǂ	0.018
25	37,064,123	*rs134623086*	dominant	1.70×10^{-8}	*LOC101904303*ǂ	0.016
25	39,833,041	*rs135726605*	dominant	4.15×10^{-8}	*FOXK1 LOC101907846 MIR2890*	0.016

25	39,846,521	*rs109120989*	additive dominant	4.59 X 10^{-8} 6.09 X 10^{-11}	*FOXK1 LOC101907846*	0.016
25	40,432,330	*rs134578134*	additive dominant	1.59 X 10^{-8} 1.49 X 10^{-8}	*SDK1*ǂ	0.018
25	41,899,146	*rs135389213*	additive dominant	1.45 X 10^{-8} 2.61 X 10^{-11}	*ELFN1*	0.018 0.018
25	42,786,653	*rs110821072*	dominant	2.19 X 10^{-10}	*LOC516442*	0.017
26	7,709,388	*rs133146678*	dominant	2.29 X 10^{-12}	*PRKG1*ǂ	0.017
26	20,714,265	*rs42086190*	additive dominant	6.63 X 10^{-11} 6.34 X 10^{-12}	*DNMBP*ǂ	0.019 0.02
26	25,939,265	*rs134817582**	dominant	5.98 X 10^{-10}	*SORCS3 TUBGCP2 ZNF511*	0.017 0.019
26	26,128,319	*rs42093370**	dominant	1.61 X 10^{-10}	*SORCS3*ǂ	0.019
26	28,800,534	*rs42434955*	dominant	8.90 X 10^{-10}		0.021
26	31,743,621	*rs110951772*	additive dominant	6.26 X 10^{-13} 1.18 X 10^{-13}	*BBIP1 MIR4680 MIR6524 PDCD4*ǂ *SHOC2*	0.018
26	33,257,060	*rs42095933**	additive dominant	4.92 X 10^{-9} 1.01 X 10^{-14}	*VTI1A*ǂ *ZDHHC6*	0.017
26	34,964,906	*rs110589571*	dominant	1.56 X 10^{-9}	*TDRD1*ǂ	0.017
26	39,381,158	*rs109525251*	dominant	2.30 X 10^{-9}		0.016
26	41,354,357	*rs136057362*	dominant	2.47 X 10^{-9}	*LOC101907208*ǂ	0.018
26	44,878,817	*rs109485638*	dominant	3.40 X 10^{-11}	*ZRANB1*ǂ	0.018
26	51,272,203	*rs134028089**	dominant	8.92 X 10^{-9}		0.019
27	7,688,299	*rs42439113*	dominant	2.33 X 10^{-9}	*AGA LOC101904157*	0.042 0.042
27	18,164,172	*rs109143919*	dominant	6.67 X 10^{-10}		0.02
27	18,991,970	*rs109928360*	additive dominant	4.12 X 10^{-13} 2.02 X 10^{-13}	*LOC107131905 MTMR7*ǂ*TRNAE-UUC*	0.024

27	21,083,026	*rs110750240*	dominant	1.59×10^{-12}		0.019 0.031
27	21,375,791	*rs132728892*	additive dominant	2.90 X 10-32 2.90 X 10-32		0.021
27	31,015,838	*rs109557135*	additive recessive	5.18×10^{-9} 2.30×10^{-8}	*UNC5D*ǂ	0.019
27	32,796,736	*rs134233957*	dominant	4.47×10^{-10}	*LOC107131169*ǂ	0.021 0.032
28	6,330,810	*rs135488223*	dominant	6.80×10^{-12}		0.016
28	18,029,952	*rs42340394*	recessive	4.86×10^{-8}	*ARID5B*ǂ	0.017
28	22,005,009	*rs110596728**	dominant	2.34×10^{-12}		0.017
28	26,156,918	*rs42146109*	additive dominant	9.66×10^{-12} 1.28×10^{-12}		0.016 0.025
28	26,832,602	*rs133727040*	recessive	7.03×10^{-9}		0.022
28	27,785,987	*rs109367560*	additive dominant	5.48×10^{-9} 4.75×10^{-12}	*CDH23*ǂ	0.018
28	29,116,183	*rs136830696*	additive dominant	1.48×10^{-9} 3.57×10^{-12}	*LOC101904940 OIT3 PLA2G12B*ǂ	0.017
28	30,040,605	*rs135182095*	additive dominant	4.49×10^{-9} 2.45×10^{-12}	*VCL*	0.017
28	31,582,482	*rs109038837*	additive recessive	5.28×10^{-11} 1.45×10^{-8}		0.022 0.022
28	34,036,966	*rs110807315*	additive dominant	2.09×10^{-8} 1.10×10^{-9}		0.023 0.033
28	34,346,919	*rs110585405*	additive dominant	2.65×10^{-8} 2.14×10^{-12}		0.017 0.029
29	7,701,671	*rs135056480*	dominant	1.01×10^{-8}		0.024
29	8,903,379	*rs133663596*	additive dominant	1.83×10^{-8} 4.10×10^{-11}		0.016 0.023
29	25,819,074	*rs110160296*	additive dominant	2.70×10^{-8} 8.39×10^{-10}		0.016 0.02
29	27,820,784	*rs43170197**	dominant	8.24×10^{-9}	*LOC787694*ǂ	0.017
29	31,640,269	*rs42708672*	additive dominant	6.09×10^{-10} 1.36×10^{-8}		0.021 0.02
29	44,722,284	*rs42188587*	additive dominant	9.55×10^{-11} 1.49×10^{-12}	*LOC104976284 SART1*	0.016 0.021

					*TSGA10IP*ǂ	
X	989,669	*rs42069602*	dominant	1.07×10^{-8}	*LOC101907172*ǂ	0.019 0.028
X	4,429,526	*rs136950148**	dominant	2.19×10^{-8}		0.022 0.024
X	10,845,015	*rs110621747*	additive<br>dominant	2.65×10^{-9}<br>9.63×10^{-11}		0.022
X	18,219,246	*rs135060536*	additive	3.79×10^{-8}	*PLAC1*ǂ	0.016
X	20,919,326	*rs134105181*	dominant	9.74×10^{-10}		0.019
X	21,073,102	*rs111006549*	dominant	8.77×10^{-9}		0.016 0.021
X	21,807,318	*rs109220334*	dominant	3.69×10^{-9}		0.015
X	21,827,945	*rs110658504*	dominant	7.97×10^{-11}	*MAGEA11*	0.016
X	25,871,732	*rs136874728*	dominant	1.20×10^{-8}		0.026 0.026
X	45,175,649	*rs134819091*	additive<br>dominant	1.77×10^{-14}<br>1.77×10^{-14}		0.017
X	52,975,251	*rs134301289*	dominant	1.25×10^{-9}	*IL1RAPL2*ǂ	0.016
X	62,588,913	*rs137706243*	dominant	1.77×10^{-9}		0.019 0.019
X	62,607,238	*rs135305113*	dominant	3.86×10^{-8}		0.016
X	65,081,915	*rs133502506*	dominant	5.61×10^{-11}	*LOC100294935*	0.016
X	71,640,703	*rs137807194*	dominant	5.13×10^{-9}	*LOC100847220*ǂ	0.016
X	86,160,589	*rs133939924*	additive<br>dominant	4.24×10^{-13}<br>4.24×10^{-13}	*FAM155B*<br>*LOC104970534*	0.016
X	90,518,686	*rs134541035**	additive<br>dominant	4.26×10^{-9}<br>1.70×10^{-11}	*RP2*ǂ	0.018 0.023
X	101,652,487	*rs135383970*	dominant	5.37×10^{-10}	*ARHGEF9*ǂ	0.016
X	128,845,516	*rs110577907*	additive<br>dominant	2.51×10^{-10}<br>9.07×10^{-11}		0.018 0.028
X	140,922,740	*rs132731146*	dominant	1.69×10^{-8}		0.015
X	141,674,669	*rs137273223*	dominant	7.45×10^{-9}		0.021 0.022

[1]Chromosome location of the locus

[2]SNP location as measured by numbered nucleotides in reference to the UMD 3.1 genome assembly (http://bovinegenome.org/?q=node/61)

[3]The *rs* number is the assigned SNP identity from the National Center for Biotechnology Information SNP database (https://www.ncbi.nlm.nih.gov/projects/SNP/; accessed 2 April 2018). [4]SNP located within previously identified copy number variations (CNVs) are denoted with an *.

[5]Genome-wide association analysis model.

[6]The most significant SNP in the locus associated with heifer conception rate is listed.

[7]Positional candidate gene(s) within +/- 19 kb of the associated SNP(s).

[ǂ] Genes with SNPs identified within their coding sequence

[8]Contribution of each SNP to the total variance

Supplementary Table 3. Loci Associated with Conception Rate at First Breeding (HCR1) and The Number of Times Bred to Achieve Pregnancy (TBRD) that Contained Transcription Factor Binding Sites.

SNP ID[1]	BTA: BP position[2]	Transcription Factor(s)[3]	Transcription Binding Factor Site[4]	RE Query[5]
rs110647268	1:11,250,683	Nrf2:MafK	TTT[T/**C**]TGA	0.01724
rs109447046	1:11,812,309	GR	AGA[**A**/C]CT	0.00892
rs43224096	1:29,618,772	HOXA5	[**A**/G]TTAG	0.04584
rs136512158	1:36,710,870	RXR-alpha	GG[**T**/G]TCA	0.00629
rs110503664	1:50,071,525	CP2	[T/**C**]TGGGAAG	0.00105
rs109491771	1:52,895,536	JunD	CT[G/**T**]GAC	0.00863
		c-Jun	[G/**T**]GACTT	0.01409
		JunB	[G/**T**]GACTT	0.01006
rs42282809	1:108,986,133	C/EBPalpha	GAGCA[C/**A**]	0.00813
rs110884711	1:122,822,402	C/EBPalpha	GAGCA[**A**/G]	0.00813
rs136894301	1:129,836,154	HNF-1C	G[**C**/A]AATTT	0.01529
rs136888926	1:157,576,622	Nkx2-1	CCTC[**C**/A]AG	0.002
		Nkx2-1	CTC[C/**A**]AG	0.00688
		IRF-2	[C/**A**]AGTGA	0.00791
rs109028820	2:6,611,760	TCF-4E	CT[G/**T**]TGCT	0.00114
rs137188435	2:26,960,403	HNF-4alpha1	ACCTT[**T**/G]G	0.00566
rs42416884	2:55,050,085	IRF-2	[**T**/G]CACTT	0.01135
rs42354740	2:58,258,750	HNF-3beta	CC[**A**/C]AACA	0.00664
rs133761371	2:65,451,280	CREMtau	[A/**G**]GCTGCTG	0.01929
		CREMtaualpha	[A/**G**]GCTGCTG	0.01929
		CREMtau1	[A/**G**]GCTGCTG	0.01929
		CREMtau2	[A/**G**]GCTGCTG	0.01929
rs109322207	2:65,493,191	C/EBPalpha	[C/**A**]TTT	0.03071
		YY1	CC[C/**A**]TTT	0.00334
rs109342415	2:85,462,609	C/EBPalpha	T[**T**/G]GCTC	0.00609
		DBP	GTT[**T**/G]GCT	0.00818
rs109065091	2:94,405,242	PRA	AA[A/**C**]AGTA	0.00277
		PRB	AA[A/**C**]AGTA	0.00277
rs135949924	3:13,676,438	GR-alpha	CT[T/**G**]T	0.03563
rs43336503	3:32,269,604	c-Jun	A[C/**A**]GTCA	0.01197
		JunB	A[C/**A**]GTCA	0.00583
rs111017447	3:35,996,083	HNF-1C	[T/**G**]CAATTT	0.01469

rs137663076	3:37,781,561	HOXA5	[G/**A**]TTAG	0.03929
rs137736954	4:6,502,615	HNF-3beta	CCAA[**A**/C]CA	0.00609
rs133821212	4:78,821,054	TCF-4E	AGCAA[C/**A**]G	0.00207
rs135524891	4:98,336,209	HNF-3beta	TGTT[G/**T**]TG	0.02236
rs136974031	4:118,420,186	CACCC-binding factor	GGGTGG[**A**/G]G	0.00097
rs109300805	5:76,167,701	CP2	CT[**A**/G]CCCAG	0.00101
rs137120693	5:88,633,335	IRF-2	A[C/**A**]GTGA	0.013
rs134948194	6:48,243,519	NF-1/L	TGC[**C**/A]A	0.01754
rs42919834	6:107,302,731	Nkx2-1	CTCA[**A**/C]G	0.00724
rs109630077	6:114,510,383	NF-AT1	GG[A/**C**]AAAAT	0.00384
rs135802025	6:118,987,625	AREB6	CGTCACC[**G**/A]G	0.00027
rs109079807	7:20,209,433	Nkx2-1	CCTCC[**A**/C]G	0.00207
rs134893796	7:62,311,969	GR-alpha	A[**C**/A]AG	0.08553
rs43518018	7:63,352,489	Nkx2-1	C[C/**T**]TGAG	0.0014
rs43519903	7:64,472,010	HNF-3beta	CAAAAC[**A**/C]	0.00804
rs134217351	7:66,177,154	c-Jun	TGAC[G/**T**]T	0.01514
		JunB	TGAC[G/**T**]T	0.00757
rs43531491	7:106,968,546	C/EBPbeta	A[**C**/A]ATT	0.04932
rs132834691	7:108,044,827	C/EBPbeta	AA[**T**/C]GT	0.07704
rs110390603	8:2,353,560	RXR-alpha	TGAAC[**A**/C]	0.0053
rs133541436	8:28,583,167	POU1F1b	[**A**/C]CAATTAT	0.01316
		POU1F1c	[**A**/C]CAATTAT	0.01316
rs133191466	8:72,863,847	IRF-2	TCAC[**T**/G]T	0.01847
rs110821231	9:52,338,179	Pax-5	GG[**G**/A]CTTG	0.02727
rs110831114	9:52,354,187	POU2F1	CTCA[**T**/C]T	0.03177
rs110029213	9:52,360,536	POU2F1	A[G/**A**]TAAG	0.03004
rs110381341	10:7,193,076	c-Ets-1	CAG[**G**/T]AAG	0.00101
rs110325782	10:29,865,159	WT1I'-KTS	C[T/**C**]CCCTC	0.01539
rs109807092	11:35,881,102	RXR-alpha	TGAA[A/**C**]C	0.00662
rs110204195	11:50,339,491	WT1I	CACACA[**C**/T]	0.00308
rs43681043	11:50,428,456	POU2F1	AATA[**A**/G]G	0.05191
rs110930462	11:96,474,351	PRB	AACA[**G**/C]TG	0.00683
		PRA	AACA[**G**/C]TG	0.00683
rs133031236	12:36,866,277	RXR-alpha	TGAAC[**C**/T]	0.00334
		FOXP3	[**C**/T]TAAATTTT	0.00777
rs133296292	12:43,939,457	GATA-1	CAG[G/**A**]TA	0.00916

rs110718934	12:58,518,230	STAT4	[**G**/T]GAAAT	0.01312
rs43711088	13:1,247,948	GR	AGT[C/**T**]CT	0.00869
rs41676079	13:2,264,173	CREMtau	CTG[C/A]AGC	0.02894
		CREMtaualpha	CTG[C/A]AGC	0.02894
		CREMtau1	CTG[C/A]AGC	0.02894
		CREMtau2	CTG[C/A]AGC	0.02894
rs41566209	13:57,799,152	HNF-3beta	[**C**/A]CAAACA	0.04406
		FOXO4	AGAAA[C/**A**]CAAACAAA	0.00031
		FOXM1b	AAA[C/**A**]CAAACAAA	0.00322
		FOXM1a	AAA[C/**A**]CAAACAAA	0.00322
		FOXJ1	AAA[C/**A**]CAAACAAAA	0.00208
		HNF-3alpha	A[C/**A**]CAAACA	0.01861
		HNF-3beta	A[C/**A**]CAAACA	0.01861
rs110288957	14:2,198,215	Nkx2-1	CCTCC[G/**A**]G	0.00386
rs41839430	14:17,291,481	TBP	T[**T**/G]TATATA	0.0216
rs135136778	14:21,358,853	HNF-4alpha1	ATCTTT[T/**G**]	0.00463
		C/EBPalpha	TT[T/**G**]CTC	0.0061
		TCF-4E	CTTT[T/**G**]CT	0.00192
rs132864771	14:58,333,285	YY1	AAATG[**G**/T]	0.00796
		C/EBPbeta	AATG[**G**/T]	0.06876
rs41662040	15:5,208,201	HNF-1C	[T/**G**]TAATTT	0.00958
rs135148389	15:15,473,841	NF-1/L	TG[A/**G**]CA	0.03093
rs41596221	15:22,458,768	GATA-2	GATA[**C**/A]AAG	0.00144
rs137402563	15:55,552,388	TFIID	T[**C**/T]TAAAA	0.021
		TFIID	TTT[C/**T**]TAA	0.03051
		TFIID	TT[C/**T**]TAAA	0.03051
		TFIID	T[C/**T**]TAAAA	0.03051
rs109416226	15:61,669,862	LF-A1	T[C/**G**]CCCTG	0.00379
rs134711585	16:15,481,171	Elk-1	[**C**/A]AACTTCCT	0.00019
rs133881641	16:16,473,798	CREMtau	CATC[C/**A**]GC	0.00648
		CREMtaualpha	CATC[C/**A**]GC	0.00648
		CREMtau1	CATC[C/**A**]GC	0.00648
		CREMtau2	CATC[C/**A**]GC	0.00648
rs42936399	16:34,026,492	JunD	[T/**G**]TCAAG	0.01479
rs134810606	16:35,683,918	TCF-4E	AGCAA[C/**A**]G	0.00319
		IRF-2	A[C/**A**]GTGA	0.01789
rs41823539	16:72,801,288	TFIID	T[**T**/G]TTCTA	0.04357

rs41639843	17:4,755,665	GR-alpha	CT[**G**/T]T	0.07855
rs109063837	17:28,295,990	PRB	G[**A**/C]CTGTT	0.00635
		PRA	G[**A**/C]CTGTT	0.00635
rs110378748	17:40,345,468	Fra-1	TGACG[**C**/A]	0.01248
rs110610109	17:46,125,255	GR-alpha	AC[**A**/C]G	0.04788
rs41844776	17:47,203,601	HNF-3beta	T[**G**/T]TTTTG	0.005
rs132676005	18:21,080,949	c-Jun	TGAC[G/**T**]C	0.01441
		c-Jun	TGAC[G/**T**]CA	0.00174
		TFIID	[G/**T**]CAAAAA	0.03029
		c-Fos	TGAC[G/**T**]CA	0.00174
rs41888920	18:55,406,165	CREMtau	CAGCAG[**A**/C]	0.02894
		CREMtau	G[A/**C**]TGCAG	0.02894
		CREMtau	[A/**C**]TGCAGC	0.02894
		CREMtaualpha	CAGCAG[A/**C**]	0.02894
		CREMtaualpha	G[A/**C**]TGCAG	0.02894
		CREMtaualpha	[A/**C**]TGCAGC	0.02894
		CREMtau1	CAGCAG[A/**C**]	0.02894
		CREMtau1	G[A/**C**]TGCAG	0.02894
		CREMtau1	[A/**C**]TGCAGC	0.02894
		CREMtau2	CAGCAG[A/**C**]	0.02894
		CREMtau2	G[A/**C**]TGCAG	0.02894
		CREMtau2	[A/**C**]TGCAGC	0.02894
rs109045973	18:62,610,581	STAT4	GG[**A**/C]AAT	0.00942
		HNF-1C	G[A/**C**]AATTT	0.00428
rs135740624	19:8,755,982	Sp1	GGG[T/**C**]GG	0.02603
rs137270020	19:24,125,771	T3R-beta	[G/**A**]GTCACT	0.00102
rs133431883	19:36,603,139	HNF-3alpha	AA[A/**C**]AAACA	0.01047
		HNF-3beta	AA[A/**C**]AAACA	0.01047
rs110291598	19:53,443,757	NF-1/L	[**T**/C]GGCA	0.03118
rs133688167	20:11,320,893	TFIID	[G/**T**]TTTTTA	0.07254
rs133727040	20:26,832,602	c-Ets-2	T[**T**/C]CCTC	0.00886
rs133442114	20:28,255,221	HNF-1C	[T/**G**]TAATTT	0.01107
rs110981497	20:66,175,689	HNF-3beta	CCAAA[A/**C**]A	0.00216
rs41960759	20:66,434,683	TFIID	TTT[G/**T**]TGA	0.04048
rs133287618	21:21,479,311	GR-alpha	[T/**C**]TGT	0.0621
		PRB	CA[T/**C**]TGTT	0.00403
		PRA	CA[T/**C**]TGTT	0.00403

rs134241191	22:6,613,617	HNF-3beta	[A/**C**]CAAACA	0.01566
rs135795816	22:39,159,742	Elk-1	CTTCCG[**C**/T]	0.02511
rs109398421	22:48,562,499	IRF-1	AAAG[**G**/T]GAAA	0.00104
rs135600509	23:11,601,873	AP-2alphaA	[A/**C**]GGGG	0.04838
rs133879598	23:40,664,436	NFI/CTF	CCA[**A**/C]ACGT	0.00495
		c-Myc	[A/**C**]ACGTG	0.00793
		USF1	[A/**C**]ACGTGAC	0.00047
rs136686460	23:42,391,390	CP2	CT[**G**/T]GGAAG	0.00117
rs109518863	23:47,335,140	HNF-4alpha1	[A/**C**]AAAGAT	0.00192
rs135250622	24:6,866,479	GR	AG[**T**/G]TCT	0.00893
rs135529640	24:34,221,005	c-Ets-2	G[**A**/C]GGAA	0.01546
rs136377593	24:37,585,202	CREMtau1	[A/**C**]AGCAGC	0.02092
		CREMtau2	[A/**C**]AGCAGC	0.02092
rs134250904	25:13,523,513	WT1I	CACACA[A/**C**]	0.02768
rs135389213	25:41,899,146	HES-1	G[T/**C**]GCGTG	0.01367
rs42293525	26:13,829,714	NF-1/L	T[**G**/A]GCA	0.02128
rs109525251	26:39,381,158	AhR	G[**C**/A]GTGAG	0.00172
		BTEB4	GCC[**C**/A]GCCCC	0.03461
rs42146109	28:26,156,918	C/EBPalpha	GAG[**C**/A]AA	0.00787
rs109038837	28:31,582,482	C/EBPalpha	TT[A/**G**]	0.49948
		C/EBPbeta	TT[A/**G**]	0.49948
		Nkx2-1	CTT[A/**G**]AG	0.00671
rs42188587	29:44,722,284	POU1F1c	ATAATTT[G/**T**]	0.01828
rs42069602	X:989,669	GR	AG[**T**/G]TCT	0.00895
rs136874728	X:25,871,732	c-Jun	TGAC[**T**/G]T	0.00991
		JunB	TGAC[**T**/G]T	0.00815
rs134819091	X:45,175,649	RXR-alpha	[**C**/T]GAACC	0.00477
rs133939924	X:86,160,589	Fra-1	TG[**A**/C]CGC	0.01301

[1]The most significant SNP in the locus associated with HCR or TBRD is listed. The *rs* number is the assigned SNP identity from the National Center for Biotechnology Information SNP database (https://www.ncbi.nlm.nih.gov/projects/SNP/; accessed 2 April 2018).

[2]Single nucleotide polymorphism (SNP) location on the *Bos taurus* (BTA) chromosome is followed by the nucleotide location in base pairs (bp) as measured by numbered nucleotides in reference to the UMD 3.1 genome assembly (http://bovinegenome.org/?q=node/61).

[3]Gene symbol for the transcription factors whose binding sites spanned the locus.

[4]Binding site for the transcription factor, with the SNP alleles in brackets. Bolded alleles are associated with the TFBS.

[5]RE Query: Random Expectation query – test statistic evaluating probability of TFBS being within a specific DNA sequence

Supplementary Table 4. Upstream Regulators and their Targets Identified in the Ingenuity Pathway Analysis for Heifer Conception Rate.

Upstream Regulators[1]	Molecule Type[2]	p-value[3]	Gene Targets of the Upstream Regulator[4]
Calcium	chemical - endogenous mammalian	4.17 x 10^{-8}	*AHR, ANKH, ATF3, CALR, CD80, CEBPB, COMMD5, CREM, DBP, ELK1, FOS, FOSL1, ICAM1, IRF1, JUN, JUNB, JUND, KCND2, NFATC2, NTRK2, SELP, STC1*
MT2	other	2.08 x 10^{-7}	*ATF3, CEBPA, ETS2, FOS, JUN, LEP, MYC*
LIF	cytokine	5.12 x 10^{-7}	*ANKRD40, CEBPB, ERAP1, FOS, FOXA2, FOXP3, GATA1, HES1, IRF1, JUN, JUNB, LARGE1, LEP, MYC, PGR, RPE65, WT1, ZFP57*
MT1	other	5.55 x 10^{-7}	*ATF3, CEBPA, ETS2, FOS, JUN, LEP, MYC*
EGF	growth factor	1.13 x 10^{-6}	*ATF3, CEBPB, CREM, ELK1, ETS1, ETS2, FOS, FOSL1, FOXA2, FOXJ1, FSCN2, GATA2, HES1, HNF4A, ICAM1, IL1R2, JUN, JUNB, JUND, MYC, PDCD4, PGR, RXRA, SP1, TBP, TFAP2A, TRPM7, VCL, ZEB1*
FOS	transcription regulator	1.49 x 10^{-6}	*ACTG1, ATF3, BCAR3, CAMK2D, CAST, CLIP1, FMN1, FOS, FOSL1, FOXA1, GRIK2, ICAM1, JUN, JUNB, LARGE1, MYC, NR3C1, PGR, PRDM1, PSMA2, PTPRD, QKI, RBP1, RXRA, SDK1, SEMA3E, SMARCD2, ST8SIA5, UTRN, ZEB1*
FLT3LG	cytokine	2.88 x 10^{-6}	*CD80, CEBPA, CEBPB, FOXP3, GATA1, ICAM1, PAX5, RAG1, RAG2*
LRP5	transmembrane receptor	3.24 x 10^{-6}	*FOXA1, FOXA2, HNF1A, HNF1B, HNF4A*

benzyloxycarbonyl-Leu-Leu-Leu aldehyde	chemical - protease inhibitor	7.46 x 10^{-6}	*ABL1, AHR, ATF3, CEBPA, CEBPB, ETS1, FOS, FOXM1, HNF1A, ICAM1, JUN, MYC, NEDD8, NKX2-1, NR3C1, PDCD4, PGR, PRDM1, RXRA, SCRIB*
CEBPA	transcription regulator	9.31 x 10^{-6}	*CEBPA, CEBPB, FOS, FOXA2, FOXM1, FTO, GATA2, HNF1A, HNF1B, ICAM1, JUN, JUNB, LEP, MYC, NFATC2, PAX5, PRDM1, QKI, RAG1, SEMA3E, TFAP2A, TGFB2, THRB, TIAM1, VCL*
acetic acid	chemical - endogenous mammalian	1.20 x 10^{-5}	*FOS, JUN, JUNB, LEP*
Integrin	complex	1.37 x 10^{-5}	*AHR, FOS, JUN, MYC, TGFB2*
TAF6	transcription regulator	1.99 x 10^{-5}	*ATF3, BCL11B, HES1, IRF1, JUN, TBP*

[1]Upstream regulators are molecules that control multiple genes in the Ingenuity Pathway Analysis through direct or indirect relationships

[2]Molecule type of the regulator as defined by the Ingenuity Pathway Analysis

[3]P-value with Bonferroni correction ($p < 1.25 \times 10^{-5}$)

[4]Gene targets of the up stream regulators from the positional candidate genes and transcription binding factor sites identified in the genome-wide association and Transfac analysis

Supplementary Table 5. Master Regulators and their Targets Identified in the Ingenuity Pathway Analysis for Heifer Conception Rate.

Master Regulators[1]	Molecule Type[2]	p-value[3]	Master Regulator Gene Targets[4]
cannabinol	chemical - endogenous mammalian	2.01×10^{-9}	*ACTG1, AFAP1, ATF1, ATF3, ATXN1, CAST, CD276, CD46, CEBPA, CEBPB, CIT, CLEC7A, CREM, ETS1, ETS2, FOS, FOSL1, FOXA1, FOXA2, FOXM1, FOXP3, GADD45GIP1, GATA1, GLCCI1, GNAQ, GRIK2, HES1, HNF1A, HNF1B, ICAM1, ICAM5, IL1R2, JUN, JUNB, JUND, KCND2, KIR3DL1, LCP1, LEP, MDGA1, MIGA1, MYC, NFATC2, NKX21, NR3C1, PAX5, PDCD4, PGR, PPP6C, PRDM1, PRKG1, PSMA2, PTPRS, QKI, RAB10, RAG2, RASGRF1, RBP1, ROBO1, SELP, SEMA3E, SEMA4A, SMARCD2, SNTB1, SORCS3, ST8SIA5, STC1, SV2C, TBP, TGFB2, TIAM1, TICRR, TNFRSF8, UTRN, WT1, WWOX, ZEB1, ZNF148*
RGS1	other	1.70×10^{-8}	*ATF6, CD46, CD80, CEBPB, CLEC7A, DBP, ETS1, FOS, HES1, HNF4A, ICAM1, ICAM3, JUN, JUNB, JUND, LEP, MYC, NR3C1, POU1F1, RBP1, RXRA, SELP, TBP, TGFB2*
WWC1	transcription regulator	1.74×10^{-8}	*ABCA4, ACER1, ACTG1, ALDH4A1, APBB2, ARHGEF9, ATF1, ATF3, ATP6V0D2, ATP6V1H, ATXN1, BCAR3, BCL11B, BRF2, CAMK2D, CAST, CD46, CDC42BPB, CEBPA, CEBPB, CHI3L2, CHMP4A, CIT, CLEC7A, CNOT4, CNTNAP2, COL14A1, DAB1, DGKI, EIF2B1, EPB41L5, EPHA3, ETS2, EYA4, FAM171A1, FEM1B, FHIT, FMN1, FOXA2, FOXJ1, FOXO4, FOXP3, GABRA1, GATA1, GATA2, GBX1, GGCT, GH1, GNAI2, GNAQ, GRIK2, HNF1A, HNF4A, ICAM1, ICAM3, ICAM5, IQGAP1, IRF1, IVNS1ABP, JUN, JUNB, JUND, KCND2, KPNA3, LCP1, LEP, LRP2, MAP3K3, MDGA1, MFAP3, MIGA1,*

			MIOX, MOG, MYC, NEDD8, NEK1, NFATC2, NLGN1, NLGN4X, PAFAH1B1, PAICS, PDCD4, PFKP, PGR, PIK3R1, PKIA, PLAC1, PLCB1, PPAT, PRKD1, PRKG1, PRKN, PTGER3, PTPN11, QKI, RAB3C, RAD23A, RAG1, RASGRF1, RBL2, RGS12, ROBO1, RPE65, RPL8, RXRA, SCRIB, SEMA4A, SHROOM3, SORCS3, SP1, SPAG9, ST3GAL5, STAT4, STC1, SUCLG1, SV2C, TCF7L2, TFAP2A, TGFB2, THRB, TIAM1, TJP2, TMED2, TNFRSF8, TPP2, UTRN, VAMP4, WNT2, ZEB1
TNFAIP8L2	other	3.33×10^{-8}	*ATF3, CD46, CD80, CEBPA, CEBPB, CXCL9, ELK1, FHIT, FOSL1, FOXM1, FOXO4, GATA2, HES1, HNF4A, ICAM1, JUN, JUNB, JUND, KLF15, LEP, MYC, NFATC2, PGR, PRDM1, RAG1, RBP1, RXRA, SORCS3, TBP, UTRN, WT1, ZEB1*
MT2	other	4.17×10^{-8}	*ATF3, CEBPA, ETS2, FOS, JUN, LEP, MYC*
UBA3	enzyme	4.20×10^{-8}	*ABCA4, ACER1, AHR, ARHGEF9, ATP6V1H, ATXN1, CAST, CD80, CDC42BPB, CEBPA, CHMP4A, CIT, CLEC7A, CNOT4, CREM, CXCL9, DBP, ELK1, EP400, EPHA3, ERAP1, ETS2, EYA4, FMN1, FOS, FOXA1, FOXO4, FOXP3, GABRA1, GATA1, GGCT, GLCCI1, GNAQ, GRIA4, HDAC9, HES1, HIP1, HNF4A, IQGAP1, KPNA3, LEP, MAP3K3, MYO10, NEK1, NFATC2, NLGN1, NLGN4X, NR3C1, PAX5, PDCD4, PFKP, PLAC1, PLCB1, PPM1H, PRDM1, PTGER3, RAB3C, RAG2, RBL2, RBP1, ROBO1, RXRA, SELP, SP1, SPAG9, STAT4, STC1, TBP, TCF7L2, TGFB2, THRB, TJP2, TPP2, VCL, WT1, ZEB1*
CLNK	other	4.83×10^{-8}	*ACTG1, AFAP1, AHR, ATF3, ATXN1, CD276, CD46, CD80, CEBPA, CLEC7A, ETS1, FOS, FOSL1, FOXA1, FOXA2, FOXM1, FOXP3, GADD45GIP1, GATA1, GNAQ, HNF1A,*

			HNF1B, HNF4A, ICAM1, ICAM5, IL1R2, IRF1, JUN, JUNB, KCND2, KIR3DL1, LCP1, LEP, MYC, NFATC2, NR3C1, PAX5, PDCD4, PGR, PPP6C, PRDM1, PSMA2, PTPRS, QKI, RAB10, RAG2, RBP1, RXRA, SELP, SEMA3E, SMARCD2, SNTB1, ST8SIA5, TGFB2, TIAM1, TICRR, TNFRSF8, UTRN, WWOX, ZNF148
SLC35B2	transporter	5.48×10^{-8}	*ATF3, CD46, CD80, CEBPA, CEBPB, CXCL9, ELK1, ETS1, FHIT, FOSL1, FOXM1, GATA2, HES1, ICAM1, JUN, JUNB, KLF15, LEP, MYC, NFATC2, PGR, PRDM1, RAG1, RBP1, SORCS3, TBP, UTRN, WT1, ZEB1*
decanoic acid	chemical - endogenous mammalian	6.65×10^{-8}	*ATF3, CD46, CD80, CEBPA, CEBPB, CXCL9, ELK1, ETS1, FHIT, FOSL1, FOXM1, GATA2, HES1, ICAM1, JUN, JUNB, KLF15, LEP, MYC, NFATC2, PGR, PRDM1, RAG1, RBP1, SORCS3, TBP, UTRN, WT1, ZEB1*
STAM2	other	6.87×10^{-8}	*ATF3, CD46, CD80, CEBPA, CEBPB, CXCL9, ELK1, FHIT, FOSL1, FOXM1, GATA2, HES1, HNF4A, ICAM1, JUN, JUNB, JUND, KLF15, LEP, MYC, NFATC2, PGR, PRDM1, RAG1, RBP1, RXRA, SORCS3, TBP, UTRN, WT1, ZEB1*
FTO	enzyme	8.77×10^{-8}	*AHR, ATF6, BCL11B, CD46, CD80, CEBPA, CXCL9, DBP, ELK1, ETS1, ETS2, FEM1B, FHIT, FOSL1, FOXA1, FOXA2, FOXM1, FOXP3, GATA2, IRF1, JUNB, KCND2, KLF15, LEP, MOG, PDCD4, PFKP, PGR, PHLPP1, POU1F1, PRDM1, RAG1, RBP1, SELP, SORCS3, SP1, TBP, TCF7L2, UTRN, VCL, WT1, ZEB1*
MT1	other	9.79×10^{-8}	*ATF3, CEBPA, ETS2, FOS, JUN, LEP, MYC*
L-type Calcium Channel	complex	1.17×10^{-7}	*AHR, ANKH, ATF1, ATF6, BCAR3, BCL11B, CALR, CAMK2D, CD80, CEBPA, CREM, DBP, ETS1, FHIT, FOS, FOSL1, FOXM1, FOXP3, GATA2, GRIK2, ICAM1, ICAM3, JUN, JUNB, JUND, KCND2, LEP, MDGA1, MIGA1, MYC,*

			NFATC2, NKX2-1, PGR, POU1F1, PRDM1, RAG1, RASGRF1, ROBO1, SELP, SEMA4A, SORCS3, SP1, STC1, SV2C, TBP, UTRN, WT1, ZEB1
PIKFYVE	kinase	1.50×10^{-7}	*AHR, ATF3, CEBPB, ELK1, ETS1, FHIT, FOS, FOSL1, FOXM1, FOXP3, GATA2, HES1, JUN, LEP, MOG, MYC, PFKP, PGR, RAG1, UTRN, WT1, ZEB1*
LATS1	kinase	1.78×10^{-7}	*ABCA4, ABLIM3, ALDH4A1, APBB2, ARHGEF9, ATF3, ATF6, ATP6V0D2, ATP6V1H, ATXN1, BCL11B, BEND5, BRF2, CACNB2, CALR, CAMK2D, CAST, CD46, CDC42BPB, CHD9, CHI3L2, CHMP4A, CIT, CNOT4, CNTNAP2, COL14A1, CREM, CTBP2, CXCL9, DAB1, DBP, DHX9, EIF2B1, EP400, FAM171A1, FHIT, FMN1, FOXA1, FOXJ1, FOXK1, FOXM1, GABRA1, GADD45GIP1, GATA1, GBX1, GH1, GNAQ, HDAC9, HIP1, HNF1A, ICAM3, ICAM5, IQGAP1, KCNIP4, KLF15, KLHL1, KPNA3, LCP1, LIMD2, LRP2, MAP3K3, MFAP3, MOG, MTMR7, MYC, MYO10, NEDD8, NEK1, NFATC2, NKX2-1, NLGN1, NLGN4X, NTNG1, NTRK2, PAICS, PARD3, PGR, PIK3R1, PKIA, PLAC1, PLCB1, PLCH1, POLD3, PPAT, PPM1H, PRKD1, PRKG1, PRKN, PTGER3, PTPN11, QKI, RAB3C, RAD23A, RAG2, RBL2, RGS12, ROBO1, RPE65, RPL8, RXRA, SBF1, SCRIB, SEMA3E, SEMA4A, SHROOM3, SORCS3, SPAG9, ST3GAL5, STAT4, STC1, SUCLG1, TBP, TFAP2A, THRB, TJP2, TNFRSF8, TPP2, UPK1B, USP33, UTRN, VAMP4, VCL, WWOX, YY1, ZEB1, ZFP57, ZNF148, ZNF804A*

NPHS1	other	2.20 x 10^{-7}	*ATF3, CD46, CD80, CEBPA, CEBPB, CXCL9, ELK1, FHIT, FOSL1, FOXM1, GATA2, HNF4A, ICAM1, JUN, JUNB, JUND, KLF15, LEP, MYC, NFATC2, NR3C1, PGR, PRDM1, RAG1, RBP1, RXRA, SORCS3, TBP, TNFRSF8, UTRN, WT1, ZEB1*
BTNL2	transmembrane receptor	2.22 x 10^{-7}	*ABCA4, ABL1, ACER1, ALDH4A1, APBB2, APPL2, ARHGAP24, ARHGEF9, ATF6, ATP6V0D2, ATP6V1H, ATXN1, BCAR3, BCL11B, BMP6, BRF2, CALR, CAMK2D, CAST, CD109, CD46, CDC42BPB, CEBPB, CHI3L2, CHMP4A, CLEC7A, CNOT4, COL14A1, DGKI, DHX9, DNMBP, EIF2B1, ELK1, EP400, ERAP1, ETS1, ETS2, EYA4, FAM208A, FHIT, FMN1, FOS, FOSL1, FOXA2, FOXJ1, FOXM1, FOXP3, GABRA1, GADD45GIP1, GATA1, GBX1, GGCT, GLCCI1, GNAI2, GNAQ, HES1, HIP1, HNF1A, HOXA5, ICAM5, IL1R2, IQGAP1, IRF2, IVNS1ABP, JUNB, KCND2, KLF15, KPNA3, LCP1, LEP, LIMD2, LNPEP, LRP2, MAGEA11, MAP3K3, MFAP3, MIOX, MOG, MTMR7, MYC, MYO10, NEDD8, NEK1, NKX2-1, NLGN1, NLGN4X, NOD1, NR3C1, PAFAH1B1, PAX5, PFKP, PGR, PHLPP1, PIK3R1, PKHD1, PKIA, PLCB1, POU1F1, PRDM1, PRKD1, PRKN, PTPN11, RAB10, RAB3C, RAG1, RAG2, RASGRF1, RBL2, RBP1, REST, RGS12, RPE65, RPL8, RXRA, SBF1, SCRIB, SELP, SEMA3E, SHROOM3, SIPA1L3, SLC38A3, SPAG9, ST3GAL5, ST8SIA5, STAT4, STC1, SV2C, TCF7L2, THRB, TIAM1, TJP2, TMED2, TNFRSF8, TPP2, USP33, UTRN, VAMP4, WT1, WWOX, ZEB1*
CDON	other	2.40 x 10^{-7}	*ATF3, CD46, CD80, CEBPB, CXCL9, ELK1, ETS1, FHIT, FOSL1, FOXA1, FOXA2, FOXM1, GATA2, HES1, ICAM1, JUN, JUNB, KLF15, LCP1, LEP, MYC, NFATC2, PGR, PRDM1,*

			RAG1, RBP1, SORCS3, TBP, UTRN, WT1, ZEB1
FZD4	G-protein coupled receptor	2.48 x 10^{-7}	*AHR, ATF3, ATF6, BCL11B, CD80, CEBPA, DBP, ETS1, FOS, FOSL1, FOXA2, GATA2, HNF1A, HNF4A, ICAM1, IRF1, JUN, JUNB, JUND, LIMD2, MTMR7, MYC, NKX2-1, POU1F1, PRDM1, RAD23A, RBP1, RXRA, SP1, TBP, WT1, ZEB1*
CARM1	transcription regulator	2.51 x 10^{-7}	*ABCA4, ABLIM3, AHR, ALDH4A1, APBB2, ARHGEF9, ATF1, ATP6V1H, ATXN1, BEND5, CALR, CAMK2D, CAST, CD80, CDC42BPB, CEBPA, CHMP4A, CIT, CLEC7A, CLSTN2, CNOT4, COL14A1, CREM, CXCL9, DBP, EIF2B1, ELK1, ERAP1, ETS1, ETS2, FMN1, FOSL1, FOXA1, FOXO4, FOXP3, GATA1, GLCCI1, GNAQ, GRIK2, HDAC9, HIP1, ICAM1, IQGAP1, JUND, KCND2, KPNA3, LEP, MAP3K3, MDGA1, MIGA1, MYC, NEDD8, NEK1, NFATC2, NKX2-1, NLGN1, NLGN4X, NR3C1, NTNG1, PDCD4, PFKP, PGR, PKIA, PLCB1, PRDM1, PRKD1, PRKG1, PRKN, PTGER3, PTPN11, RAB3C, RAD23A, RASGRF1, RBL2, RBP1, RGS12, ROBO1, SCRIB, SELP, SEMA4A, SHROOM3, SORCS3, SP1, SPAG9, STAT4, STC1, SUCLG1, SV2C, TBP, TGFB2, THRB, TJP2, TPP2, VCL, WNT2, WT1, ZEB1*
NEIL1	enzyme	3.04 x 10^{-7}	*AHR, ALDH4A1, ANKH, APBB2, API5, ATF1, ATF3, ATF6, ATXN1, BCL11B, CALR, CAMK2D, CAST, CD46, CD80, CIT, COL14A1, CXCL9, DBP, EIF2B1, ELK1, ERAP1, ETS2, FOS, FOSL1, FOXA1, FOXM1, FOXO4, FOXP3, GATA1, GATA2, GH1, GLCCI1, GRIK2, HDAC9, HES1, HIP1, ICAM1, IRF1, JUN, JUNB, JUND, KCND2, KLF15, LEP, MDGA1, MIGA1, MYO10, NEDD8, NFATC2, NKX2-1, NR3C1, PAX5, PFKP, PRDM1,*

			PRKD1, PRKG1, PRKN, PTPN11, RAB3C, RAD23A, RAG1, RAG2, RASGRF1, RBL2, RBP1, RGS12, RXRA, SCRIB, SELP, SEMA4A, SHROOM3, SORCS3, SP1, STC1, SUCLG1, SV2C, TBP, TCF7L2, TGFB2, TJP2, VCL, WNT2, WT1, YY1, ZEB1
VGF	growth factor	3.10×10^{-7}	*ATF1, ATF3, CD46, CD80, CEBPA, CEBPB, CREM, CXCL9, DGKI, ELK1, ERAP1, ETS1, FHIT, FOS, FOSL1, FOXM1, FOXP3, GATA2, GRIK2, HES1, ICAM1, JUN, JUNB, JUND, KCND2, KLF15, LEP, MDGA1, MIGA1, MYC, NFATC2, NKX2-1, PGR, PRDM1, PRKG1, QKI, RAG1, RASGRF1, RBP1, ROBO1, SEMA4A, SORCS3, STC1, SV2C, TBP, TCF7L2, UTRN, WT1, ZEB1*
GNRH2	other	3.29×10^{-7}	*ABCA4, ARHGEF9, ATF3, ATP6V1H, ATXN1, BCAR3, CDC42BPB, CEBPA, CHMP4A, CIT, CLEC7A, CNOT4, ELK1, EP400, ETS1, FHIT, FMN1, FOSL1, FOXM1, FOXO4, FOXP3, GABRA1, GATA2, GNAQ, ICAM3, IQGAP1, IRF1, JUN, JUNB, JUND, KPNA3, LEP, MAP3K3, MYO10, NEK1, NLGN1, NLGN4X, PDCD4, PFKP, PGR, PLCB1, PPM1H, PRDM1, PTGER3, RAG1, RBL2, ROBO1, SP1, SPAG9, STC1, TCF7L2, TGFB2, THRB, TJP2, TPP2, UTRN, VCL, WT1*
DYRK1A	kinase	3.58×10^{-7}	*AFAP1, AHR, ALDH4A1, APBB2, ATF1, ATF3, ATXN1, BCL11B, CAMK2D, CD46, CD80, COL14A1, CREM, CXCL9, EIF2B1, ELK1, ETS1, ETS2, FHIT, FOSL1, FOXA2, GATA2, GRIK2, HDAC9, HES1, HNF1A, HNF1B, HNF4A, HOXA5, ICAM1, ICAM5, IL1R2, IRF1, JUN, KCND2, KCNIP4, KLHL1, LRP2, MDGA1, MIGA1, NEDD8, NFATC2, NKX2-1, NR3C1, NTRK2, PAX5, PFKP, PIK3R1, POU1F1, PRKD1, PRKG1, PRKN, PTPN11, QKI, RAD23A, RAG1, RASGRF1,*

			RBL2, RGS12, RPE65, RXRA, SCRIB, SELP, SEMA4A, SHROOM3, SIPA1L3, SORCS3, STC1, SUCLG1, SV2C, SYNE1, TBP, TCF7L2, TGFB2, TIAM1, UTRN, VCL, WNT2, WT1, WWOX, ZNF148
SERCA	group	4.44×10^{-7}	*ATF3, BCAR3, CALR, CD46, CD80, CEBPA, CEBPB, CXCL9, ELK1, FHIT, FOSL1, FOXM1, FOXP3, GATA2, HES1, ICAM3, JUN, KLF15, LCP1, LEP, MYC, NFATC2, PGR, RAG1, RBP1, SORCS3, TBP, UTRN, WT1, ZEB1*
Ca2+	chemical - endogenous mammalian	4.93×10^{-7}	*AHR, ANKH, ATF3, CALR, CD80, CEBPB, COMMD5, CREM, DBP, ELK1, FOS, ICAM1, JUNB, JUND, KCND2, NFATC2, NTRK2, SELP, STC1*
NOV	growth factor	5.15×10^{-7}	*ATF3, CD46, CD80, CEBPA, CEBPB, CXCL9, ELK1, FHIT, FOSL1, FOXM1, GATA2, HES1, HNF4A, ICAM1, JUN, JUNB, JUND, KLF15, LEP, MYC, NFATC2, PGR, PRDM1, RAG1, RBP1, RXRA, SORCS3, TBP, UTRN, WT1, ZEB1*
DGCR8	enzyme	5.32×10^{-7}	*ACTG1, AHR, ATF3, ATF6, CALR, CD80, ETS1, FHIT, FOS, FOSL1, FOXM1, GATA1, GATA2, GMPR, HES1, HNF1A, HNF1B, HNF4A, ICAM1, IL1R2, IVNS1ABP, JUND, KCNN2, LEP, MYC, NR3C1, PDCD4, PGR, PPM1H, PRDM1, PSMA2, RAG1, RAG2, RBP1, RXRA, SEMA3E, SMARCD2, ST8SIA5, TBP, TCF7L2, TFAP2A, UTRN, WT1, ZEB1*
PARP1	enzyme	5.83×10^{-7}	*AHR, ALDH4A1, ANKH, APBB2, API5, ATF1, ATF3, ATF6, ATXN1, BCL11B, CALR, CAMK2D, CAST, CD46, CD80, CIT, COL14A1, CXCL9, DBP, EIF2B1, ELK1, ERAP1, ETS2, FOS, FOSL1, FOXA1, FOXM1, FOXO4, FOXP3, GATA1, GATA2, GH1, GLCCI1, GRIK2, HDAC9, HES1, HIP1, ICAM1, IRF1,*

			JUN, JUNB, JUND, KCND2, KLF15, MDGA1, MIGA1, MYO10, NEDD8, NFATC2, NKX2-1, NR3C1, PAX5, PFKP, PRDM1, PRKD1, PRKG1, PRKN, PTPN11, RAB3C, RAD23A, RAG1, RAG2, RASGRF1, RBL2, RBP1, RGS12, RXRA, SCRIB, SELP, SEMA4A, SHROOM3, SORCS3, SP1, STC1, SUCLG1, SV2C, TBP, TCF7L2, TGFB2, TJP2, VCL, WNT2, WT1, YY1, ZEB1
RPS6KA1	kinase	5.95×10^{-7}	*ABCA4, AHR, ARHGEF9, ATF1, ATP6V1H, BMP6, CAMK2D, CD80, CDC42BPB, CEBPA, CHMP4A, CIT, CLEC7A, CNOT4, CSRP2, CXCL9, DBP, ELK1, EPHA3, ERAP1, ETS1, FMN1, FOXA2, FOXM1, FOXO4, FOXP3, GH1, GMPR, GNAQ, GRIA4, GRIK2, HDAC9, HIP1, HNF4A, IQGAP1, IVNS1ABP, JUND, KCND2, KCNN2, KPNA3, MAP3K3, MDGA1, MIGA1, NEK1, NFATC2, NKX2-1, NLGN1, NLGN4X, NOD1, NR3C1, PAFAH1B1, PAX5, PDCD4, PGR, PHLPP1, PKHD1, PLCB1, POU1F1, PPM1H, PRDM1, PSMA2, PTGER3, RAB10, RAB3C, RAG2, RASGRF1, RBL2, RBP1, RXRA, SEMA3E, SEMA4A, SMARCD2, SORCS3, SP1, SPAG9, ST8SIA5, STAT4, STC1, SV2C, TCF7L2, TFAP2A, TGFB2, THRB, TJP2, TPP2, UBA6, VCL, WNT2, WT1*
DDX54	transcription regulator	6.11×10^{-7}	*ABCA4, ACER1, ARHGEF9, ATF3, ATP6V1H, ATXN1, CDC42BPB, CEBPA, CHMP4A, CIT, CLEC7A, CNOT4, EP400, EPHA3, EYA4, FMN1, FOS, FOXA1, FOXO4, FOXP3, GABRA1, GGCT, GNAQ, HES1, HNF1B, IQGAP1, JUN, JUNB, JUND, KPNA3, LEP, MAP3K3, MYC, MYO10, NEK1, NLGN1, NLGN4X, PDCD4, PFKP, PLAC1, PLCB1, PPM1H, PTGER3, RBL2, RBP1, ROBO1, SP1, SPAG9, STC1, TCF7L2, TFAP2A, TGFB2, THRB, TJP2, TPP2, VCL, ZEB1*

GRM5	G-protein coupled receptor	7.28 x 10^{-7}	*AHR, ATF1, ATF6, BCAR3, CALR, CD46, CD80, CREM, CXCL9, DAB1, DBP, DGKI, ERAP1, ETS1, FHIT, FOS, FOSL1, FOXA2, FOXP3, GATA2, HES1, ICAM1, ICAM3, JUN, JUNB, JUND, KLF15, LEP, MYC, NFATC2, NR3C1, PAFAH1B1, PAX5, PGR, POU1F1, PRDM1, PRKG1, QKI, RAG1, RBP1, SORCS3, SP1, TBP, UPK1B, UTRN, WT1, ZEB1*
RIT1	enzyme	7.30 x 10^{-7}	*ATF3, BMP6, CD46, CD80, CEBPA, CEBPB, CXCL9, ELK1, EPHA3, ETS1, FHIT, FOSL1, FOXM1, GATA2, HES1, ICAM1, JUN, JUNB, KLF15, LEP, MYC, NFATC2, NR3C1, PGR, PRDM1, RAG1, RBP1, SORCS3, TBP, UTRN, WT1, ZEB1*
MCAM	other	7.47 x 10^{-7}	*ATF3, CEBPB, DAB1, ELK1, ETS1, FHIT, FOSL1, FOXM1, FOXP3, GATA2, HES1, ICAM1, ICAM5, JUN, LEP, MYC, PGR, RAG1, UTRN, VCL, WT1, ZEB1*
iodide	chemical - endogenous mammalian	8.15 x 10^{-7}	*ATF3, CD46, CD80, CEBPA, CEBPB, CXCL9, DBP, ETS1, FOS, ICAM1, JUN, JUNB, KLF15, LEP, MYC, NFATC2, NKX2-1, NR3C1, POU1F1, PRDM1, RBP1, SORCS3, TBP*
Frizzled	group	8.16 x 10^{-7}	*AHR, ATF3, ATF6, BCL11B, CEBPA, CEBPB, DBP, ELK1, ETS1, FOS, FOSL1, FOXA2, FOXO4, GATA2, HES1, HNF1A, JUN, JUNB, JUND, LEP, LIMD2, MTMR7, NKX2-1, POU1F1, PRDM1, RAD23A, RBP1, SP1, TBP, TCF7L2, WT1, ZEB1*

HOXB7	transcription regulator	8.19×10^{-7}	*ABCA4, ABL1, ACER1, ACTG1, ADGRE3, AFAP1, AGAP3, ALDH4A1, ANXA7, APBB2, APPL2, ARHGEF9, ATF1, ATF6, ATP6V1H, ATP7B, ATXN1, BCL11B, CAMK2D, CAST, CD276, CDC42BPB, CEBPA, CEBPB, CHD9, CHMP4A, CIT, CLEC7A, CLSTN2, CNOT4, CNTNAP2, CSRP2, DGKI, EIF2B1, ELK1, EPHA3, ETS1, EYA4, FAM171A1, FEM1B, FHIT, FMN1, FOSL1, FOXO4, FOXP3, FRK, GABRA1, GATA1, GATA2, GGCT, GH1, GIMAP6, GLCCI1, GMPR, GNAI2, GNAQ, GRIK2, GYPC, HES1, HNF1B, ICAM5, IL1R2, IQGAP1, IRF1, IVNS1ABP, JUNB, JUND, KCNIP4, KCNN2, KLF15, KLHL1, KPNA3, LCP1, LIMD2, LNPEP, LUC7L3, MAGEA11, MAP3K3, MDGA1, MIGA1, MOG, MTMR7, MYC, MYO10, NEDD8, NEK1, NFIA, NLGN1, NLGN4X, NR3C1, PAFAH1B1, PAICS, PFKP, PGR, PHLPP1, PKHD1, PKIA, PLAC1, PPAT, PPM1H, PRDM1, PRKD1, PRKG1, PRKN, PTGER3, PTPN11, QKI, RAB10, RAG1, RASGRF1, RGS12, ROBO1, SCRIB, SDK1, SELP, SHROOM3, SLC25A15, SLC38A3, SNTB1, SORCS3, SP1, SPAG9, ST3GAL5, STC1, SUCLG1, SV2C, SWT1, TFAP2A, TICRR, TJP2, TMED2, UTRN, VCL, WNT2, WWOX, YY1, ZEB1, ZFP57, ZNF148*
TAF4	transcription regulator	8.63×10^{-7}	*ACTG1, AHR, ATF1, ATF3, CD80, CEBPB, CNOT4, CREM, CXCL9, DBP, ELK1, ERAP1, FHIT, FOS, FOXA1, FOXA2, FOXM1, FOXP3, GATA2, GRIK2, HDAC9, HES1, HIP1, HNF1A, HNF1B, HOXA5, ICAM1, IRF1, JUN, JUNB, JUND, KCND2, LEP, MDGA1, MIGA1, MYC, NFIA, NKX2-1, PDCD4, PGR, PPAT, PRKG1, PSMA2, RAB3C, RAG1, RASGRF1, RBP1, ROBO1, RPE65, SEMA3E, SEMA4A, SMARCD2, SORCS3, ST8SIA5, STAT4, STC1, SV2C, TFAP2A, TGFB2, UTRN, WT1, ZEB1*

APBB1	transcription regulator	8.73×10^{-7}	*ABCA4, ACER1, ALDH4A1, APBB2, ARHGEF9, ATF1, ATF3, ATF6, ATP6V1H, ATXN1, BCAR3, BCL11B, CAMK2D, CD46, CDC42BPB, CEBPA, CEBPB, CHMP4A, CIT, CLEC7A, CNOT4, COL14A1, EIF2B1, EPB41L5, EPHA3, ETS2, EYA4, FEM1B, FHIT, FMN1, FOS, FOXA1, FOXJ1, FOXM1, FOXO4, FOXP3, GATA2, GGCT, GNAQ, GRIK2, HES1, ICAM1, ICAM3, ICAM5, IL1R2, IQGAP1, IRF1, IVNS1ABP, JUN, JUNB, JUND, KCND2, KLF15, KPNA3, LEP, MAP3K3, MDGA1, MIGA1, MOG, MYC, NEDD8, NEK1, NFATC2, NKX2-1, NLGN1, NLGN4X, PAX5, PDCD4, PGR, PLAC1, PLCB1, PRKD1, PRKN, PTGER3, PTPN11, RAD23A, RAG1, RASGRF1, RBL2, RBP1, RGS12, ROBO1, RPE65, RXRA, SCRIB, SEMA4A, SHROOM3, SLC38A3, SORCS3, SP1, SPAG9, ST3GAL5, STC1, SUCLG1, SV2C, TCF7L2, TFAP2A, TGFB2, THRB, TJP2, TNFRSF8, TPP2, UTRN, WNT2, YY1, ZEB1*
KLF9	transcription regulator	9.02×10^{-7}	*ABCA4, ARHGEF9, ATF3, ATP6V1H, ATXN1, CDC42BPB, CEBPA, CHMP4A, CIT, CLEC7A, CNOT4, EP400, FMN1, FOS, FOXM1, FOXO4, FOXP3, GABRA1, GNAQ, HES1, IQGAP1, IRF1, JUN, JUNB, JUND, KPNA3, LEP, MAP3K3, MYC, MYO10, NEK1, NLGN1, NLGN4X, PDCD4, PFKP, PGR, PLCB1, PPM1H, PTGER3, RBL2, ROBO1, SP1, SPAG9, STC1, TCF7L2, TGFB2, THRB, TJP2, TPP2, VCL, ZEB1*
NDNF	other	9.42×10^{-7}	*ATF3, CD80, CEBPB, ELK1, ETS1, FHIT, FOS, FOSL1, FOXM1, GATA2, HES1, ICAM1, JUN, LEP, MYC, PGR, RAG1, UTRN, WT1, ZEB1*
pCPT-cAMP	chemical - kinase inhibitor	9.99×10^{-7}	*CD80, CEBPA, CEBPB, CREM, FOS, FOSL1, FOXA2, GRIK2, HNF4A, IRF1, JUN, JUNB,*

			JUND, LEP, PGR, PIK3R1, POU1F1, PRKG1, RBL2, SLC38A3
ICAM3	transmembrane receptor	1.10 x 10^{-6}	*AHR, ATF3, ATF6, BCL11B, CEBPB, DBP, ELK1, ETS1, FHIT, FOSL1, FOXM1, GATA2, HES1, JUN, JUNB, LEP, MYC, PGR, POU1F1, RAG1, SP1, UTRN, WT1, ZEB1*
PD 180970	chemical - kinase inhibitor	1.10 x 10^{-6}	*ABCA4, ACTG1, AHR, ALDH4A1, APBB2, ARHGEF9, ATF6, ATP6V1H, ATXN1, BCAR3, BCL11B, BMP6, BRF2, CACNB2, CAMK2D, CD46, CD80, CDC42BPB, CEBPA, CEBPB, CHI3L2, CHMP4A, CIT, CNOT4, COL14A1, CREM, CSRP2, CXCL9, DAB1, DGKI, EIF2B1, ELK1, ETS2, EYA4, F2RL3, FEM1B, FHIT, FMN1, FOXJ1, FOXM1, FOXP3, GATA1, GBX1, GH1, GLCCI1, GNAQ, HIP1, ICAM1, ICAM3, ICAM5, IQGAP1, IRF2, JUNB, KCND2, KCNIP4, KLF15, KLHL1, KPNA3, LCP1, LEP, LRP2, LUC7L3, MAP3K3, MFAP3, MOG, MYC, NEDD8, NEK1, NFATC2, NLGN1, NLGN4X, NTRK2, PAX5, PDCD4, PGR, PHLPP1, PIK3R1, PLCB1, PLCH1, POU1F1, PRDM1, PRKD1, PRKN, PSMA2, PTGER3, PTPN11, PTPRS, QKI, RAB3C, RAG2, RBL2, RBP1, RGS12, ROBO1, RPE65, RPL8, SCRIB, SHROOM3, SMARCD2, SORCS3, SPAG9, ST8SIA5, STC1, SUCLG1, TFAP2A, TGFB2, THRB, TIAM1, TNFRSF8, TPP2, UPK1B, UTRN, VAMP4, VCL, WNT2, YY1, ZEB1*
USP43	peptidase	1.11 x 10^{-6}	*ABCA4, ABL1, AFAP1, AHR, ALDH4A1, APBB2, API5, ARHGEF9, ATF1, ATF3, ATP6V0D2, ATP6V1H, ATP7B, BRF2, CAMK2D, CAST, CD276, CD46, CD80, CDC42BPB, CEBPB, CHI3L2, CHMP4A, CIT, CLEC7A, CNOT4, CREM, CXCL9, DBP, DGKI, EIF2B1, ELK1, EPB41L5, EPHA3, ERAP1, ETS1, ETS2, FEM1B, FHIT, FMN1, FOS,*

			FOXA1, FOXO4, GADD45GIP1, GATA1, GATA2, GH1, GLCCI1, GNAI2, GNAQ, GRIA4, GRIK2, HDAC9, HIP1, HNF1A, HNF1B, ICAM1, ICAM3, ICAM5, IL1R2, IQGAP1, IRF1, IRF2, IVNS1ABP, JUN, JUNB, KCND2, KLF15, KPNA3, LARGE1, LCP1, LNPEP, MAGEA11, MAP3K3, MDGA1, MIGA1, MSS51, MYO10, NEDD8, NEK1, NKX2-1, NLGN1, NLGN4X, NR3C1, PAICS, PAX5, PHLPP1, PIK3R1, PKHD1, PLCB1, POLD3, POU1F1, PPAT, PRDM1, PRKD1, PRKG1, PRKN, PSMA2, PTGER3, PTPN11, RAB3C, RAD23A, RASGRF1, RGS12, RPE65, RPL8, SCRIB, SEMA3E, SEMA4A, SHROOM3, SNTB1, SORCS3, SPAG9, STAT4, SUCLG1, SV2C, TFAP2A, TGFB2, THRB, TIAM1, TICRR, TJP2, TNFRSF8, UTRN, VAMP4, VCL, WNT2, WT1, WWOX, YY1, ZNF148
LIF	cytokine	1.11×10^{-6}	*ANKRD40, CEBPB, ERAP1, FOS, FOXA2, FOXP3, IRF1, JUN, JUNB, LARGE1, LEP, MYC, PGR, RPE65, WT1, ZFP57*
TBX21	transcription regulator	1.15×10^{-6}	*ACTG1, ATXN1, BCAR3, BCL11B, CD276, CD80, CEBPA, CEBPB, CLEC7A, CLIP1, CXCL9, ELK1, FHIT, FNIP2, FOSL1, FOXA1, FOXA2, FOXM1, GATA2, GNAQ, HES1, ICAM3, IL1R2, IPO4, IRF1, JUND, LEP, LPIN2, MYO10, NFATC2, NR3C1, PAX5, PFKP, PGR, PPP6C, PRDM1, PSMA2, RAG1, RAG2, SCRIB, SELP, SEMA4A, SGCD, SNTB1, ST3GAL5, STAT4, TGFB2, TJP2, UTRN, WT1, ZEB1*
Gαq	group	1.16×10^{-6}	*AHR, ATF3, ATF6, BCL11B, CEBPA, CEBPB, CREM, CXCL9, DBP, ETS1, FOSL1, GNAQ, ICAM1, IRF1, JUN, JUNB, MYC, PGR, POU1F1, PRKG1, SELP, SP1, VCL*

IQUB	other	1.16 x 10^{-6}	*ACTG1, AHR, CD46, CD80, CEBPA, CEBPB, CSRP2, CXCL9, ELK1, ERAP1, ETS1, FHIT, FNBP1L, FOS, FOXM1, FSCN2, GATA2, HES1, HNF4A, ICAM1, IRF1, JUNB, KLF15, LEP, MYC, NFATC2, PGR, PRDM1, RAG1, RBP1, SELP, SORCS3, TBP, TJP2, UTRN, VCL, ZEB1*
DYNLL1	other	1.18 x 10^{-6}	*ABCA4, AHR, API5, ARHGEF9, ATF3, ATP6V1H, BCAR3, BRF2, CD80, CDC42BPB, CEBPA, CEBPB, CHI3L2, CHMP4A, CIT, CLEC7A, CNOT4, CREM, CXCL9, DBP, ELK1, ERAP1, FMN1, FOS, FOSL1, FOXO4, FOXP3, GNAQ, HDAC9, HES1, HIP1, HNF1A, HNF1B, ICAM3, IQGAP1, IRF1, JUN, JUNB, JUND, KPNA3, LEP, MAP3K3, NEK1, NLGN1, NLGN4X, PDCD4, PGR, PIK3R1, PLCB1, PRDM1, PTGER3, RAB3C, RBL2, ROBO1, RPL8, RXRA, SP1, SPAG9, STAT4, TBP, TCF7L2, TGFB2, THRB, TJP2, TPP2, UTRN*
2,5-dihydroxymethylcinnamate	chemical - kinase inhibitor	1.19 x 10^{-6}	*ATF3, CD80, CEBPB, ELK1, ETS1, FHIT, FOS, FOSL1, FOXM1, GATA2, HES1, ICAM1, JUN, LEP, MYC, PGR, RAG1, UTRN, WT1, ZEB1*
FSTL1	other	1.36 x 10^{-6}	*ANKH, ATF3, CD46, CD80, CEBPA, CEBPB, CXCL9, ELK1, FHIT, FOSL1, FOXM1, FOXP3, GATA2, HES1, HNF4A, JUN, JUNB, JUND, KLF15, LEP, NFATC2, PGR, PRDM1, RAG1, RBP1, RXRA, SORCS3, SP1, TBP, TGFB2, UTRN, WT1, ZEB1*
Plk	group	1.51 x 10^{-6}	*ABCA4, ACTG1, ANKH, ARHGEF9, ATF1, ATF3, ATF6, ATP6V0D2, ATP6V1H, BCAR3, BCL11B, BMP6, BRF2, CALR, CD46, CDC42BPB, CHI3L2, CHMP4A, CIT, CLEC7A, CNOT4, CNTNAP2, CSRP2, EP400, EPHA3, ERAP1, ETS2, F5, FHIT, FMN1, FOS, FOXA1, FOXJ1, FOXK1, FOXO4, FOXP3, GABRA1, GLCCI1, GNAI2, GNAQ, GRIK2, HNF1B,*

			HNF4A, ICAM1, ICAM3, IL1R2, IQGAP1, IRF2, IVNS1ABP, JUNB, KCND2, KCNIP4, KLF15, KLHL1, KPNA3, LEP, LIMD2, LRP2, MAP3K3, MDGA1, MIGA1, MTMR7, NEK1, NFATC2, NLGN1, NLGN4X, NOD1, NR3C1, PAFAH1B1, PAICS, PAX5, PHLPP1, PIK3R1, PKHD1, PKIA, PLCB1, PPAT, PRDM1, PSMA2, PTGER3, PTPRS, QKI, RAB10, RAG1, RAG2, RASGRF1, RBP1, RPE65, RPL8, RXRA, SBF1, SEMA3E, SLC38A3, SMARCD2, SPAG9, ST8SIA5, STC1, SV2C, THRB, TIAM1, TMED2, TPP2, UBE2E3, UPK1B, UTRN, VAMP4, WWOX, ZFP57
KRT5	other	1.52×10^{-6}	*ABCA4, ALDH4A1, ANKH, APBB2, ARHGEF9, ATF1, ATF3, ATP6V0D2, ATP6V1H, ATXN1, BCAR3, BMP6, BRF2, CACNB2, CAST, CDC42BPB, CHD9, CHI3L2, CHMP4A, CIT, CLEC7A, CLSTN2, CNOT4, COL14A1, DBP, DGKI, DHX9, EIF2B1, EPHA3, ERAP1, ETS1, ETS2, EYA4, F2RL3, FAM171A1, FAM208A, FHIT, FMN1, FOS, FOSL1, FOXJ1, FOXO4, FOXP3, GABRA1, GATA1, GH1, GLCCI1, GNAI2, GNAQ, GRAP2, GRIK2, HDAC9, HIP1, HNF1A, HNF1B, HNF4A, ICAM1, ICAM3, IL1R2, IQGAP1, IVNS1ABP, JUN, JUNB, JUND, KPNA3, LNPEP, MAGEA11, MDGA1, MIGA1, MIOX, NEDD8, NEK1, NKX2-1, NLGN1, NLGN4X, NTRK2, PAFAH1B1, PDCD4, PHLPP1, PKHD1, PKIA, PLAC1, PLCB1, PLCH1, POU1F1, PRKD1, PRKG1, PRKN, PTGER3, PTPN11, QKI, RAB3C, RAD23A, RAG2, RASGRF1, REST, RGS12, RPL8, RXRA, SCRIB, SEMA4A, SHROOM3, SMARCD2, SORCS3, SPAG9, ST3GAL5, ST8SIA5, STAT4, STC1, SUCLG1, SV2C, TBP, TFAP2A, THRB, TJP2, TMED2, TNFRSF8, TPP2, TXNDC5,*

			UBA6, USP33, UTRN, VAMP4, WNT2, YY1, ZEB1
quinolinic acid	chemical - endogenous mammalian	1.54 x 10^{-6}	*AHR, ATF3, CAMK2D, CD80, CEBPA, CREM, CXCL9, DBP, ELK1, ERAP1, FHIT, FOS, FOSL1, FOXM1, FOXP3, GATA2, HDAC9, HES1, HIP1, ICAM1, IRF1, JUN, JUNB, JUND, LEP, MYC, NFATC2, NTRK2, PGR, PRDM1, RAB3C, RAG1, SELP, STAT4, UTRN, ZEB1*
SGCG	other	1.56 x 10^{-6}	*ABCA4, ALDH4A1, ANKH, APBB2, ARHGEF9, ATF1, ATF3, ATP6V0D2, ATP6V1H, ATXN1, BCAR3, BMP6, BRF2, CACNB2, CAST, CDC42BPB, CHD9, CHI3L2, CHMP4A, CIT, CLEC7A, CLSTN2, CNOT4, COL14A1, DBP, DGKI, DHX9, EIF2B1, EPHA3, ERAP1, ETS1, ETS2, EYA4, F2RL3, FAM171A1, FAM208A, FHIT, FMN1, FOS, FOSL1, FOXJ1, FOXO4, FOXP3, GABRA1, GATA1, GH1, GLCCI1, GNAI2, GNAQ, GRAP2, GRIK2, HDAC9, HIP1, HNF1A, HNF1B, HNF4A, ICAM3, IL1R2, IQGAP1, IVNS1ABP, JUN, JUNB, JUND, KPNA3, LNPEP, MAGEA11, MDGA1, MIGA1, MIOX, NEDD8, NEK1, NKX2-1, NLGN1, NLGN4X, NTRK2, PAFAH1B1, PDCD4, PHLPP1, PKHD1, PKIA, PLAC1, PLCB1, PLCH1, POU1F1, PRKD1, PRKG1, PRKN, PTGER3, PTPN11, QKI, RAB3C, RAD23A, RAG2, RASGRF1, REST, RGS12, RPL8, RXRA, SCRIB, SEMA4A, SGCD, SHROOM3, SMARCD2, SORCS3, SPAG9, ST3GAL5, ST8SIA5, STAT4, STC1, SUCLG1, SV2C, TBP, TFAP2A, THRB, TJP2, TMED2, TNFRSF8, TPP2, TXNDC5, UBA6, USP33, UTRN, VAMP4, WNT2, YY1, ZEB1*

GRIN1	ion channel	1.56 x 10^{-6}	*AHR, BCAR3, BMP6, CD46, CD80, CEBPA, CEBPB, CREM, CXCL9, DBP, EPHA3, ERAP1, ETS1, FHIT, FOSL1, FOXM1, FOXP3, GATA2, HDAC9, HES1, HIP1, ICAM1, ICAM3, IRF1, JUN, JUNB, KLF15, LEP, MYC, NFATC2, NLGN1, NR3C1, NTRK2, PFKP, PGR, PRDM1, RAB3C, RAG1, RBP1, SORCS3, STAT4, TBP, UTRN, VCL, WT1, ZEB1*
Integrin	complex	1.58 x 10^{-6}	*AHR, FOS, JUN, MYC, TGFB2*
IRF6	transcription regulator	1.59 x 10^{-6}	*AGA, AHR, ATF3, ATXN1, CALR, CAMK2D, CAST, CD46, CD80, CREM, CXCL9, DBP, ELK1, ETS1, FOS, FOSL1, FOXP3, ICAM1, IRF1, JUN, JUNB, JUND, KLF15, KPNA3, LEP, MARCH3, MED12L, MYC, NFATC2, NR3C1, PGR, POU1F1, PRDM1, RBP1, SELP, SORCS3, TBP, VTI1A, ZEB1*
PRMT2	enzyme	1.61 x 10^{-6}	*ABCA4, ACER1, AFAP1, AHR, API5, ARHGEF9, ATF1, ATF3, ATF6, ATP6V1H, ATP7B, BCAR3, BCL11B, BRF2, CD276, CD46, CD80, CDC42BPB, CEBPA, CEBPB, CHI3L2, CHMP4A, CIT, CLEC7A, CNOT4, CREM, CXCL9, DBP, DGKI, ELK1, EPB41L5, EPHA3, ERAP1, ETS1, EYA4, FHIT, FMN1, FOXA1, FOXA2, FOXJ1, FOXM1, FOXO4, FOXP3, GADD45GIP1, GATA1, GATA2, GGCT, GNAQ, GRIK2, HDAC9, HIP1, HNF1A, HNF1B, ICAM1, ICAM3, IL1R2, IQGAP1, IRF1, IRF2, IVNS1ABP, JUN, JUNB, JUND, KCND2, KLF15, KPNA3, LARGE1, LCP1, LEP, LNPEP, MAP3K3, MDGA1, MIGA1, MSS51, NEK1, NKX2-1, NLGN1, NLGN4X, NTRK2, PAICS, PDCD4, PGR, PHLPP1, PIK3R1, PLAC1, PLCB1, PRDM1, PSMA2, PTGER3, QKI, RAB3C, RASGRF1, RBL2, ROBO1, RPE65, RPL8, SEMA3E, SEMA4A, SNTB1, SORCS3, SP1, SPAG9, ST3GAL5, STAT4, STC1, SV2C, TCF7L2, TGFB2, THRB, TIAM1,*

			TICRR, TJP2, TNFRSF8, TPP2, UTRN, VAMP4, WWOX, YY1, ZNF148
Nfatc	group	1.68 x 10^{-6}	*AHR, ATF3, FOS, FOXA1, FOXA2, FOXP3, HNF1A, HNF1B, HNF4A, IRF1, JUN, KCND2, MYC, PRDM1, PTPRS, RAB10, UTRN*
ganglioside GD3	chemical - endogenous mammalian	1.71 x 10^{-6}	*CD80, CEBPB, DBP, ELK1, ETS1, FHIT, FOSL1, FOXM1, GATA2, HES1, ICAM1, JUN, JUNB, LEP, MYC, NR3C1, PGR, POU1F1, RAG1, UTRN, VCL, WT1, ZEB1*
S100A12	other	1.74 x 10^{-6}	*AHR, ATF3, CD46, CD80, CEBPA, CEBPB, CREM, CXCL9, DBP, ELK1, ERAP1, FOS, FOSL1, FOXP3, HDAC9, HES1, HIP1, HNF4A, ICAM1, IRF1, JUN, JUNB, JUND, KLF15, LEP, MYC, NFATC2, NR3C1, PGR, POU1F1, PRDM1, RAB3C, RBP1, RXRA, SORCS3, STAT4, TBP*
PLAT	peptidase	1.75 x 10^{-6}	*ACTG1, AHR, ATF6, CALR, CD46, CD80, CLEC7A, CREM, CXCL9, DBP, ELK1, ERAP1, ETS1, FOS, FOSL1, FOXA2, FOXP3, GATA2, HDAC9, HES1, HIP1, HNF1A, ICAM1, ICAM3, ICAM5, JUN, JUND, KCND2, KLF15, LEP, LIMD2, MOG, MTMR7, MYC, NFATC2, NKX2-1, NOD1, NR3C1, NTRK2, PDCD4, PFKP, PHLPP1, PRDM1, PSMA2, RAB3C, RAD23A, RBP1, RXRA, SEMA3E, SMARCD2, SORCS3, ST8SIA5, STAT4, TBP, WT1, ZEB1*

TRIM45	other	1.79×10^{-6}	*AFAP1, AHR, ANKH, ATXN1, CD276, CD46, CD80, CEBPA, CLEC7A, CSRP2, ETS1, FOS, FOSL1, FOXA2, FOXM1, FOXP3, GADD45GIP1, GATA1, GH1, GNAQ, ICAM1, ICAM5, IL1R2, IRF1, JUN, JUNB, JUND, KIR3DL1, LCP1, LEP, MYC, NFATC2, NR3C1, PAX5, PDCD4, PGR, PPP6C, PRDM1, PSMA2, QKI, RAG2, RBP1, RXRA, SELP, SEMA3E, SMARCD2, SNTB1, ST8SIA5, TGFB2, TIAM1, TICRR, TJP2, TNFRSF8, VCL, WT1, WWOX, ZNF148*
PTPN5	phosphatase	1.79×10^{-6}	*ATF3, CD46, CD80, CEBPA, CEBPB, CXCL9, DAB1, DBP, ETS1, FOXP3, ICAM1, JUN, JUNB, KLF15, LEP, MYC, NFATC2, NR3C1, POU1F1, PRDM1, RBP1, SORCS3, TBP*
CD22	transmembrane receptor	1.80×10^{-6}	*ABCA4, ABL1, AFAP1, ALDH4A1, APBB2, ARHGEF9, ATP6V0D2, ATP6V1H, ATXN1, BMP6, CAMK2D, CAST, CD276, CD80, CDC42BPB, CHMP4A, CIT, CLEC7A, CLSTN2, CNOT4, COL14A1, DAB1, DHX9, EIF2B1, EPHA3, ETS1, EYA4, F2RL3, FMN1, FOSL1, GABRA1, GADD45GIP1, GATA1, GNAI2, GNAQ, GRAP2, GRIA4, HNF1A, HNF1B, HOXA5, ICAM1, ICAM5, IL1R2, IQGAP1, IRF1, IRF2, IVNS1ABP, JUN, JUNB, JUND, KCND2, KIR3DL1, KPNA3, LCP1, LIMD2, LNPEP, MIOX, MTMR7, MYC, MYO10, NEDD8, NEK1, NKX2-1, NLGN1, NLGN4X, NR3C1, NTRK2, PAFAH1B1, PAICS, PHLPP1, PKIA, PLCB1, POLD3, PPAT, PPP6C, PRDM1, PRKD1, PRKN, PSMA2, PTGER3, PTPN11, PTPRS, RAB10, RAD23A, RAG2, RGS12, ROBO1, RP2, SCRIB, SDK1, SEMA3E, SHROOM3, SMARCD2, SNTB1, SPAG9, ST8SIA5, SUCLG1, THRB, TIAM1, TICRR, TJP2, TMED2, TNFRSF8, TPP2, TXNDC5, USP33, VAMP4, WNT2, WWOX, YY1, ZNF148*

WAS	other	1.80 x 10^{-6}	*ACTG1, AFAP1, ATF3, ATXN1, CD276, CEBPB, CLEC7A, CXCL9, ERAP1, ETS1, FOS, FOSL1, FOXA1, FOXA2, FOXM1, FOXP3, GADD45GIP1, GATA1, GNAQ, HNF1A, HNF1B, ICAM1, ICAM5, IL1R2, JUN, JUNB, KCND2, KIR3DL1, KLF15, LCP1, LEP, MYC, NR3C1, PAX5, PDCD4, PGR, PPP6C, PRDM1, PRKG1, PSMA2, PTPRS, QKI, RAB10, RAG2, RXRA, SEMA3E, SMARCD2, SNTB1, SORCS3, ST8SIA5, TBP, TGFB2, TIAM1, TICRR, TNFRSF8, UTRN, WT1, WWOX, ZNF148*
GP9	other	1.82 x 10^{-6}	*ABCA4, AGA, ALDH4A1, ANKH, APBB2, ARHGEF9, ATF1, ATF3, ATF6, ATP6V0D2, ATP6V1H, BCAR3, BCL11B, BRF2, CACNB2, CD46, CD80, CDC42BPB, CHD9, CHI3L2, CHMP4A, CIT, CLEC7A, CLSTN2, CNOT4, COL14A1, DBP, DGKI, DHX9, EIF2B1, EPB41L5, ERAP1, ETS2, FAM171A1, FAM208A, FHIT, FMN1, FOS, FOXJ1, FOXO4, FOXP3, GABRA1, GATA1, GH1, GLCCI1, GNAI2, GNAQ, GRAP2, GRIK2, HDAC9, HIP1, HNF1A, HNF1B, ICAM1, IL1R2, IQGAP1, IRF2, IVNS1ABP, JUN, JUNB, JUND, KLF15, LIMD2, LNPEP, LRP2, LUC7L3, MAGEA11, MDGA1, MED12L, MIGA1, MIOX, MOG, MTMR7, MYC, NEDD8, NEK1, NLGN1, NLGN4X, NOD1, NR3C1, NTRK2, PAFAH1B1, PDCD4, PHLPP1, PKHD1, PKIA, PLAC1, PLCB1, PLCH1, POU1F1, PRKD1, PRKG1, PRKN, PTGER3, PTPN11, QKI, RAB3C, RAD23A, RAG2, RASGRF1, REST, RGS12, RPE65, RPL8, SCRIB, SELP, SEMA4A, SHROOM3, SMARCD2, SORCS3, SPAG9, ST3GAL5, ST8SIA5, STAT4, STC1, SUCLG1, SV2C, TFAP2A, THRB, TJP2, TMED2, TNFRSF8, TPP2, UBA6, USP33, UTRN, VTI1A, WNT2, YY1, ZEB1*

VEGFD	growth factor	1.84×10^{-6}	*ATF3, ATF6, CALR, CD46, CD80, CXCL9, ETS1, FHIT, FOSL1, FOXM1, GATA2, HES1, HNF4A, ICAM1, JUN, JUNB, JUND, KLF15, LEP, MYC, NFATC2, PGR, PRDM1, RAG1, RBP1, SORCS3, TBP, UTRN, VCL, WT1, ZEB1*
PDE3B	enzyme	1.84×10^{-6}	*ABCA4, ABL1, ACER1, API5, ARHGEF9, ATF6, ATP6V0D2, ATP6V1H, BCAR3, BMP6, CALR, CAMK2D, CD80, CDC42BPB, CEBPA, CEBPB, CHMP4A, CIT, CLASP2, CNOT4, CREM, CSRP2, DAB1, DHX9, EPHA3, ETS2, EYA4, FAM171A1, FIGN, FMN1, FOS, FOXA2, GADD45GIP1, GATA1, GATA2, GGCT, GH1, GLCCI1, GNAQ, GRIA4, GRIK2, HNF1A, HNF1B, ICAM1, ICAM3, ICAM5, IL1R2, IQGAP1, IRF2, JUN, JUNB, KCND2, KPNA3, LCP1, LEP, LRP2, MAP3K3, MDGA1, MIGA1, MOG, MYC, NEK1, NKX2-1, NLGN1, NLGN4X, NOD1, NTRK2, PAX5, PDCD4, PFKP, PGR, PHLPP1, PIK3R1, PKHD1, PLAC1, PLCB1, PTPRS, RAG1, RAG2, RASGRF1, RPE65, RXRA, SEMA3E, SEMA4A, SLC38A3, SP1, SPAG9, SV2C, TFAP2A, THRB, TIAM1, TNFRSF8, TPP2, TRABD2B, USP33, UTRN, YY1*
Phe-Pro-Arg-chloromethyl ketone	chemical - protease inhibitor	1.85×10^{-6}	*CEBPB, DBP, ELK1, ETS1, FHIT, FOSL1, FOXM1, GATA2, HES1, ICAM1, JUN, JUNB, LEP, MYC, NR3C1, PGR, POU1F1, RAG1, UTRN, WT1, ZEB1*
KCNA3	ion channel	1.93×10^{-6}	*ACTG1, AHR, ALDH4A1, APBB2, ARHGAP24, ATF1, ATF6, ATP6V0D2, ATXN1, BMP6, CAMK2D, CAST, CD46, CEBPA, CIT, COL14A1, CXCL9, EIF2B1, EPHA3, ERAP1, ETS2, FHIT, FOSL1, FOXA1, FOXA2, FOXP3, GABRA1, GATA1, GATA2, GH1, GLCCI1, GNAI2, GRIK2, HIP1, HNF1B, HNF4A, ICAM1, ICAM5, IL1R2, JUNB, JUND, KCND2, LEP, LIMD2, LNPEP, LRP2,*

			MAGEA11, MDGA1, MIGA1, MTMR7, MYC, NEDD8, NFATC2, NKX2-1, NR3C1, PAFAH1B1, PAX5, PDCD4, PFKP, PKHD1, PKIA, PRDM1, PRKD1, PRKG1, PSMA2, PTPN11, RAB3C, RAG1, RAG2, RASGRF1, RBL2, RBP1, RGS12, ROBO1, RPE65, RXRA, SCRIB, SELP, SEMA3E, SEMA4A, SHROOM3, SLC38A3, SMARCD2, SORCS3, SP1, ST8SIA5, STAT4, STC1, SUCLG1, SV2C, TBP, TCF7L2, THRB, TMED2, TNFRSF8, UTRN, VCL, WNT2
HDAC7	transcription regulator	1.93×10^{-6}	*ABCA4, ARHGEF9, ATF3, ATP6V1H, CDC42BPB, CEBPB, CHMP4A, CIT, CLEC7A, CNOT4, FMN1, FOSL1, FOXA2, FOXM1, FOXO4, FOXP3, GATA2, GMPR, GNAQ, HES1, HNF1A, ICAM1, IQGAP1, IRF1, IVNS1ABP, JUNB, JUND, KCNN2, KPNA3, LEP, LIMD2, MAP3K3, MTMR7, MYC, NEK1, NKX2-1, NLGN1, NLGN4X, NR3C1, PDCD4, PGR, PLCB1, PPM1H, PRDM1, PTGER3, RAD23A, RBL2, ROBO1, SP1, SPAG9, TCF7L2, TFAP2A, TGFB2, THRB, TJP2, TPP2, WT1, ZEB1*
REL/RELA/RELB	group	1.95×10^{-6}	*AGA, AHR, ATF1, ATF3, ATXN1, BMP6, BRF2, CAMK2D, CD46, CD80, CEBPB, CHI3L2, COL14A1, CREM, CXCL9, DBP, EPB41L5, ERAP1, FHIT, FOS, FOXA1, FOXO4, GATA2, GLCCI1, GNAQ, GRIA4, GRIK2, HIP1, HNF1B, HNF4A, ICAM1, IRF1, JUN, JUNB, JUND, KCND2, KIR3DL1, KPNA3, MDGA1, MED12L, MIGA1, MYC, NKX2-1, NR3C1, PAX5, PDCD4, PGR, PHLPP1, PIK3R1, PPP6C, PRDM1, RAB3C, RAG1, RAG2, RASGRF1, ROBO1, RPE65, RPL8, RXRA, SELP, SEMA4A, SORCS3, STAT4, STC1, SV2C, TBP, TFAP2A, TNFRSF8, UTRN, VCL, VTI1A, WT1, ZEB1*

CLEC7A	transmembrane receptor	1.96×10^{-6}	*AGA, AHR, ATF3, ATXN1, BCAR3, CAMK2D, CD46, CD80, CEBPA, CEBPB, CREM, CXCL9, DBP, ELK1, ERAP1, FOS, FOXA1, FOXA2, HDAC9, HES1, HIP1, ICAM1, ICAM3, IRF1, IRF2, JUN, JUNB, JUND, KLF15, KPNA3, LEP, MED12L, MYC, NFATC2, NKX2-1, NR3C1, PRDM1, PTPRS, RAB10, RAB3C, RBP1, RXRA, SELP, SORCS3, SP1, STAT4, TBP, TNFRSF8, VTI1A, WT1*
Glycoprotein 1B	complex	1.96×10^{-6}	*ABCA4, AGA, ALDH4A1, ANKH, APBB2, ARHGEF9, ATF1, ATF3, ATF6, ATP6V0D2, ATP6V1H, BCAR3, BCL11B, BRF2, CACNB2, CD46, CD80, CDC42BPB, CHD9, CHI3L2, CHMP4A, CIT, CLEC7A, CLSTN2, CNOT4, COL14A1, DBP, DGKI, DHX9, EIF2B1, EPB41L5, ERAP1, ETS2, FAM171A1, FAM208A, FHIT, FMN1, FOS, FOXJ1, FOXO4, FOXP3, GABRA1, GATA1, GH1, GLCCI1, GNAI2, GNAQ, GRAP2, GRIK2, HDAC9, HIP1, HNF1A, HNF1B, ICAM1, IL1R2, IQGAP1, IRF2, IVNS1ABP, JUN, JUNB, JUND, KLF15, LIMD2, LNPEP, LRP2, LUC7L3, MAGEA11, MDGA1, MED12L, MIGA1, MIOX, MOG, MTMR7, MYC, NEDD8, NEK1, NLGN1, NLGN4X, NOD1, NR3C1, NTRK2, PAFAH1B1, PDCD4, PHLPP1, PKHD1, PKIA, PLAC1, PLCB1, PLCH1, POU1F1, PRKD1, PRKG1, PRKN, PTGER3, PTPN11, QKI, RAB3C, RAD23A, RAG2, RASGRF1, REST, RGS12, RPE65, RPL8, SCRIB, SELP, SEMA4A, SHROOM3, SMARCD2, SORCS3, SPAG9, ST3GAL5, ST8SIA5, STAT4, STC1, SUCLG1, SV2C, TFAP2A, THRB, TJP2, TMED2, TNFRSF8, TPP2, UBA6, USP33, UTRN, VTI1A, WNT2, YY1, ZEB1*
RGS4	other	1.97×10^{-6}	*ATF3, ATF6, BCAR3, CALR, CAMK2D, CD46, CD80, CREM, CXCL9, DBP, FHIT, FOSL1, FOXM1, FOXP3, GATA2, GNAQ, HES1,*

			HNF4A, ICAM1, ICAM3, JUN, JUNB, JUND, KLF15, NFATC2, NR3C1, PGR, POU1F1, PRDM1, RAG1, RBP1, RXRA, SORCS3, TBP, UTRN, WT1
WNT4	cytokine	1.99×10^{-6}	*ATF3, CD46, CD80, CEBPA, CEBPB, CXCL9, FOS, GRIK2, HES1, HNF4A, ICAM1, JUN, JUNB, JUND, KLF15, LEP, MYC, NFATC2, PAX5, PRDM1, RBP1, RXRA, SORCS3, TBP, WT1*
AHRR	other	2.01×10^{-6}	*ABCA4, AHR, ARHGEF9, ATF3, ATF6, ATP6V1H, CALR, CD46, CD80, CDC42BPB, CEBPA, CHMP4A, CIT, CLEC7A, CNOT4, COL14A1, CREM, CTBP2, CXCL9, EPB41L5, FAM171A1, FHIT, FMN1, FOSL1, FOXA2, FOXM1, FOXO4, FOXP3, GADD45GIP1, GATA2, GMPR, GNAQ, HDAC9, HES1, HNF1A, ICAM3, IQGAP1, IRF1, IVNS1ABP, JUN, JUNB, JUND, KCNN2, KLF15, KPNA3, LEP, MAP3K3, MYC, NEK1, NFATC2, NLGN1, NLGN4X, NR3C1, PDCD4, PGR, PLCB1, POU1F1, PPM1H, PRDM1, PTGER3, QKI, RAG1, RBP1, ROBO1, SORCS3, SP1, SPAG9, TBP, TCF7L2, TFAP2A, TGFB2, THRB, TIAM1, TJP2, TPP2, UTRN, VCL, WT1*
ZNF366	transcription regulator	2.02×10^{-6}	*AHR, ATF1, ATF6, ATXN1, BMP6, BRF2, CALR, CD46, CEBPA, CEBPB, CHD9, CHI3L2, COL14A1, CXCL9, EP400, EPB41L5, ERAP1, FHIT, FOS, FOXA1, FOXA2, FOXO4, GABRA1, GATA1, GATA2, GLCCI1, GRIA4, GRIK2, HES1, HNF1A, HNF1B, HNF4A, ICAM1, ICAM3, ICAM5, IL1R2, IRF1, JUN, JUNB, KCND2, LIMD2, LRP2, MDGA1, MIGA1, MTMR7, MYC, MYO10, NFATC2, NKX2-1, NR3C1, PFKP, PGR, PHLPP1, PIK3R1, PPM1H, PRDM1, RAD23A, RAG1, RASGRF1, ROBO1, RPE65, RPL8, SELP,*

			SEMA4A, SORCS3, STC1, SV2C, TFAP2A, TNFRSF8, UTRN, VCL, WT1, ZEB1
NECTIN2	transmembrane receptor	2.02 x 10^{-6}	*ATF3, CD46, CD80, CEBPA, CXCL9, ELK1, FHIT, FOS, FOSL1, FOXM1, GATA2, HNF4A, ICAM1, JUNB, JUND, KLF15, NFATC2, PGR, PRDM1, RAG1, RBP1, RXRA, SORCS3, TBP, UTRN, WT1, ZEB1*
IC87114	chemical - kinase inhibitor	2.06 x 10^{-6}	*ATP6V0D2, CEBPB, ELK1, ETS1, FHIT, FOS, FOSL1, FOXM1, GATA2, HES1, ICAM1, JUN, LEP, MYC, PDCD4, PGR, PRDM1, RAG1, UTRN, WT1, ZEB1*
NEK10	kinase	2.13 x 10^{-6}	*AHR, ATF3, CEBPA, CEBPB, DBP, ELK1, FOS, FOSL1, JUN, JUNB, JUND, LEP, MYC, NR3C1, POU1F1, SP1*
TMEM184A	other	2.16 x 10^{-6}	*ANKH, ATF3, BCAR3, CD46, CD80, CEBPA, CEBPB, CXCL9, FOS, FOSL1, FOXP3, GH1, HES1, HNF4A, ICAM1, ICAM3, JUN, JUNB, JUND, KLF15, LEP, MYC, NFATC2, PRDM1, RBP1, RXRA, SORCS3, TBP*
ANO6	ion channel	2.17 x 10^{-6}	*ABCA4, ALDH4A1, ANKH, APBB2, ARHGEF9, ATF1, ATF3, ATF6, ATP6V0D2, ATP6V1H, ATXN1, BCAR3, CACNB2, CALR, CD46, CDC42BPB, CHD9, CHMP4A, CIT, CLEC7A, CLSTN2, CNOT4, COL14A1, CSRP2, DBP, DGKI, DHX9, EIF2B1, ERAP1, ETS2, FAM171A1, FAM208A, FHIT, FMN1, FOS, FOXJ1, FOXO4, GABRA1, GATA1, GH1, GLCCI1, GNAI2, GNAQ, GRAP2, GRIK2, HDAC9, HIP1, HNF1A, HNF1B, HNF4A, ICAM3, IL1R2, IQGAP1, IRF2, IVNS1ABP, JUN, JUNB, JUND, KLF15, KPNA3, LNPEP, LRP2, MAGEA11, MDGA1, MIGA1, MIOX, NEDD8, NEK1, NKX2-1, NLGN1, NLGN4X, NR3C1, NTRK2, PAFAH1B1, PDCD4,*

			PHLPP1, PIK3R1, PKHD1, PKIA, PLAC1, PLCB1, PLCH1, POU1F1, PRKD1, PRKG1, PRKN, PTGER3, PTPN11, PTPRS, QKI, RAB3C, RAD23A, RAG2, RASGRF1, REST, RGS12, RPE65, RXRA, SCRIB, SELP, SEMA4A, SHROOM3, SMARCD2, SORCS3, SPAG9, ST3GAL5, ST8SIA5, STAT4, STC1, SUCLG1, SV2C, TBP, TFAP2A, THRB, TJP2, TMED2, TNFRSF8, TPP2, UBA6, USP33, UTRN, WNT2, WT1, YY1, ZEB1
VTCN1	other	2.19×10^{-6}	*BCAR3, CD80, CEBPB, ELK1, FHIT, FOSL1, FOXM1, FOXP3, GATA2, HES1, HNF4A, ICAM1, ICAM3, JUN, JUNB, JUND, LEP, MYC, PGR, PRDM1, RAG1, RBP1, RXRA, TBP, UTRN, WT1, ZEB1*
SLC39A4	transporter	2.24×10^{-6}	*ACTG1, ALDH4A1, ANKH, APBB2, ARHGAP24, ATF1, ATF3, ATF6, ATXN1, BCAR3, BMP6, CALR, CAMK2D, CD46, CD80, CEBPA, CEBPB, CIT, COL14A1, CREM, CXCL9, EIF2B1, EPHA3, ERAP1, ETS2, FHIT, FOS, FOXA1, FOXM1, FOXO4, FOXP3, GATA1, GH1, GLCCI1, GRIK2, HDAC9, HIP1, HNF4A, ICAM1, ICAM3, ICAM5, IL1R2, IRF2, JUNB, JUND, KCND2, LEP, LNPEP, LRP2, MAGEA11, MDGA1, MIGA1, MYC, NEDD8, NFATC2, NR3C1, PAX5, PDCD4, PFKP, PGR, PKHD1, POU1F1, PRDM1, PRKD1, PRKG1, PSMA2, PTPN11, QKI, RAB3C, RAG1, RASGRF1, RBL2, RGS12, ROBO1, RPE65, RXRA, SCRIB, SELP, SEMA4A, SHROOM3, SLC38A3, SMARCD2, SORCS3, SP1, ST8SIA5, STAT4, STC1, SUCLG1, SV2C, TBP, TFAP2A, THRB, TIAM1, UPK1B, UTRN, VAMP4, WNT2, YY1*
TNFRSF10A	transmembrane receptor	2.25×10^{-6}	*AHR, ATF3, CD46, CD80, CEBPA, CEBPB, CREM, CXCL9, DBP, ERAP1, FHIT, FOSL1, FOXM1, FOXO4, GATA2, HDAC9, HES1,*

			HIP1, HNF4A, ICAM1, IRF1, JUN, JUNB, JUND, KLF15, LEP, NFATC2, NR3C1, PGR, POU1F1, PRDM1, RAB3C, RAG1, RBP1, RXRA, SELP, SORCS3, STAT4, TBP, UTRN, WT1, ZEB1
SLC27A1	transporter	2.33 x 10^{-6}	*AHR, ATF3, ATF6, BCL11B, CD80, CEBPA, CEBPB, DBP, ELK1, ETS1, FOS, FOSL1, FOXO4, FOXP3, ICAM1, IRF1, JUN, JUNB, JUND, KCND2, LEP, MOG, PFKP, PHLPP1, POU1F1, RBP1, SP1, TBP*
EDN2	growth factor	2.49 x 10^{-6}	*ABCA4, ALDH4A1, ANKH, APBB2, ARHGEF9, ATF1, ATF3, ATP6V0D2, ATP6V1H, ATXN1, BCAR3, BMP6, BRF2, CACNB2, CAST, CDC42BPB, CHD9, CHI3L2, CHMP4A, CIT, CLEC7A, CLSTN2, CNOT4, COL14A1, DBP, DGKI, DHX9, EIF2B1, EPHA3, ERAP1, ETS1, ETS2, EYA4, F2RL3, FAM171A1, FAM208A, FHIT, FMN1, FOS, FOSL1, FOXJ1, FOXO4, FOXP3, GABRA1, GATA1, GH1, GLCCI1, GNAI2, GNAQ, GRAP2, GRIK2, HDAC9, HIP1, HNF1A, HNF1B, HNF4A, ICAM3, IL1R2, IQGAP1, IVNS1ABP, JUN, JUNB, JUND, KPNA3, LNPEP, MAGEA11, MDGA1, MIGA1, MIOX, NEDD8, NEK1, NKX2-1, NLGN1, NLGN4X, NTRK2, PAFAH1B1, PDCD4, PHLPP1, PKHD1, PKIA, PLAC1, PLCB1, PLCH1, POU1F1, PRKD1, PRKG1, PRKN, PTGER3, PTPN11, QKI, RAB3C, RAD23A, RAG2, RASGRF1, REST, RGS12, RPL8, RXRA, SCRIB, SEMA4A, SHROOM3, SMARCD2, SORCS3, SPAG9, ST3GAL5, ST8SIA5, STAT4, STC1, SUCLG1, SV2C, TBP, TFAP2A, THRB, TJP2, TMED2, TNFRSF8, TPP2, TXNDC5, UBA6, USP33, UTRN, VAMP4, WNT2, YY1, ZEB1*

CNN3	other	2.49×10^{-6}	*ABCA4, ALDH4A1, ANKH, APBB2, ARHGEF9, ATF1, ATF3, ATP6V0D2, ATP6V1H, ATXN1, BCAR3, BMP6, BRF2, CACNB2, CAST, CDC42BPB, CHD9, CHI3L2, CHMP4A, CIT, CLEC7A, CLSTN2, CNOT4, COL14A1, DBP, DGKI, DHX9, EIF2B1, EPHA3, ERAP1, ETS1, ETS2, EYA4, F2RL3, FAM171A1, FAM208A, FHIT, FMN1, FOS, FOSL1, FOXJ1, FOXO4, FOXP3, GABRA1, GATA1, GH1, GLCCI1, GNAI2, GNAQ, GRAP2, GRIK2, HDAC9, HIP1, HNF1A, HNF1B, HNF4A, ICAM3, IL1R2, IQGAP1, IVNS1ABP, JUN, JUNB, JUND, KPNA3, LNPEP, MAGEA11, MDGA1, MIGA1, MIOX, NEDD8, NEK1, NKX2-1, NLGN1, NLGN4X, NTRK2, PAFAH1B1, PDCD4, PHLPP1, PKHD1, PKIA, PLAC1, PLCB1, PLCH1, POU1F1, PRKD1, PRKG1, PRKN, PTGER3, PTPN11, QKI, RAB3C, RAD23A, RAG2, RASGRF1, REST, RGS12, RPL8, RXRA, SCRIB, SEMA4A, SHROOM3, SMARCD2, SORCS3, SPAG9, ST3GAL5, ST8SIA5, STAT4, STC1, SUCLG1, SV2C, TBP, TFAP2A, THRB, TJP2, TMED2, TNFRSF8, TPP2, TXNDC5, UBA6, USP33, UTRN, VAMP4, WNT2, YY1, ZEB1*
APOH	transporter	2.51×10^{-6}	*ABCA4, AFAP1, AHR, ALDH4A1, ANXA7, APBB2, API5, ARHGEF9, ARID5B, ATF3, ATP6V1H, ATP7B, ATXN1, BCAR3, BRF2, CACNB2, CAMK2D, CAST, CD276, CD46, CD80, CDC42BPB, CEBPA, CEBPB, CHI3L2, CHMP4A, CIT, CLSTN2, CNOT4, CNTNAP2, CREM, CSRP2, CXCL9, DAB1, DBP, DHX9, EIF2B1, ELK1, EP400, EPB41L5, EPHA3, ERAP1, ETS1, F2RL3, FEM1B, FMN1, FOXA1, FOXJ1, FOXP3, GABRA1, GATA1, GBX1, GLCCI1, GMPR, GNAI2, GNAQ, HDAC9, HIP1, HNF1B, ICAM3, ICAM5, IL1R2,*

			IQGAP1, IRF1, IRF2, IVNS1ABP, JUN, JUNB, KCNN2, KLF15, KPNA3, LARGE1, LCP1, LEP, LUC7L3, MAGEA11, MAP3K3, MAPKAP1, MARCH3, MDGA1, MFAP3, MIGA1, MOG, MSS51, MYC, MYO10, NEDD8, NEK1, NFATC2, NLGN1, NLGN4X, NOD1, NSG1, PAFAH1B1, PAICS, PIK3R1, PLCB1, PLCH1, PPAT, PPM1H, PRDM1, PRKD1, PRKN, PSMA2, PTGER3, PTPN11, PTPRS, RAB10, RAB3C, RAD23A, RAG2, RASGRF1, RBP1, RGS12, RPL8, SCRIB, SELP, SHROOM3, SLC38A3, SMARCD2, SNTB1, SORCS3, SPAG9, ST3GAL5, ST8SIA5, STAT4, SUCLG1, SV2C, TBP, TFAP2A, THRB, TIAM1, TICRR, TJP2, TNFRSF8, UPK1B, USP33, WNT2, WWOX, YY1, ZNF148
PTGFRN	other	2.53×10^{-6}	*ABCA4, ALDH4A1, ANKH, APBB2, ARHGEF9, ATF1, ATF3, ATP6V0D2, ATP6V1H, ATXN1, BCAR3, BMP6, BRF2, CACNB2, CAST, CDC42BPB, CHD9, CHI3L2, CHMP4A, CIT, CLEC7A, CLSTN2, CNOT4, COL14A1, DBP, DGKI, DHX9, EIF2B1, EPHA3, ERAP1, ETS1, ETS2, EYA4, F2RL3, FAM171A1, FAM208A, FHIT, FMN1, FOS, FOSL1, FOXJ1, FOXO4, FOXP3, GABRA1, GATA1, GH1, GLCCI1, GNAI2, GNAQ, GRAP2, GRIK2, HDAC9, HIP1, HNF1A, HNF1B, HNF4A, ICAM3, IL1R2, IQGAP1, IVNS1ABP, JUN, JUNB, JUND, KPNA3, LNPEP, MAGEA11, MDGA1, MIGA1, MIOX, NEDD8, NEK1, NKX2-1, NLGN1, NLGN4X, NTRK2, PAFAH1B1, PDCD4, PHLPP1, PKHD1, PKIA, PLAC1, PLCB1, PLCH1, POU1F1, PRKD1, PRKG1, PRKN, PTGER3, PTPN11, QKI, RAB3C, RAD23A, RAG2, RASGRF1, REST, RGS12, RPL8, RXRA, SCRIB, SEMA4A, SHROOM3, SMARCD2, SORCS3, SPAG9, ST3GAL5, ST8SIA5, STAT4,*

			STC1, SUCLG1, SV2C, TBP, TFAP2A, THRB, TJP2, TMED2, TNFRSF8, TPP2, TXNDC5, UBA6, USP33, UTRN, VAMP4, WNT2, YY1, ZEB1
COL5A3	other	2.53 x 10^{-6}	*ABCA4, ALDH4A1, ANKH, APBB2, ARHGEF9, ATF1, ATF3, ATP6V0D2, ATP6V1H, ATXN1, BCAR3, BMP6, BRF2, CACNB2, CAST, CDC42BPB, CHD9, CHI3L2, CHMP4A, CIT, CLEC7A, CLSTN2, CNOT4, COL14A1, DBP, DGKI, DHX9, EIF2B1, EPHA3, ERAP1, ETS1, ETS2, EYA4, F2RL3, FAM171A1, FAM208A, FHIT, FMN1, FOS, FOSL1, FOXJ1, FOXO4, FOXP3, GABRA1, GATA1, GH1, GLCCI1, GNAI2, GNAQ, GRAP2, GRIK2, HDAC9, HIP1, HNF1A, HNF1B, HNF4A, ICAM3, IL1R2, IQGAP1, IVNS1ABP, JUN, JUNB, JUND, KPNA3, LNPEP, MAGEA11, MDGA1, MIGA1, MIOX, NEDD8, NEK1, NKX2-1, NLGN1, NLGN4X, NTRK2, PAFAH1B1, PDCD4, PHLPP1, PKHD1, PKIA, PLAC1, PLCB1, PLCH1, POU1F1, PRKD1, PRKG1, PRKN, PTGER3, PTPN11, QKI, RAB3C, RAD23A, RAG2, RASGRF1, REST, RGS12, RPL8, RXRA, SCRIB, SEMA4A, SHROOM3, SMARCD2, SORCS3, SPAG9, ST3GAL5, ST8SIA5, STAT4, STC1, SUCLG1, SV2C, TBP, TFAP2A, THRB, TJP2, TMED2, TNFRSF8, TPP2, TXNDC5, UBA6, USP33, UTRN, VAMP4, WNT2, YY1, ZEB1*

lewis Y	chemical - endogenous mammalian	2.53 x 10^{-6}	*ABCA4, ALDH4A1, ANKH, APBB2, ARHGEF9, ATF1, ATF3, ATP6V0D2, ATP6V1H, ATXN1, BCAR3, BMP6, BRF2, CACNB2, CAST, CDC42BPB, CHD9, CHI3L2, CHMP4A, CIT, CLEC7A, CLSTN2, CNOT4, COL14A1, DBP, DGKI, DHX9, EIF2B1, EPHA3, ERAP1, ETS1, ETS2, EYA4, F2RL3, FAM171A1, FAM208A, FHIT, FMN1, FOS, FOSL1, FOXJ1, FOXO4, FOXP3, GABRA1, GATA1, GH1, GLCCI1, GNAI2, GNAQ, GRAP2, GRIK2, HDAC9, HIP1, HNF1A, HNF1B, HNF4A, ICAM3, IL1R2, IQGAP1, IVNS1ABP, JUN, JUNB, JUND, KPNA3, LNPEP, MAGEA11, MDGA1, MIGA1, MIOX, NEDD8, NEK1, NKX2-1, NLGN1, NLGN4X, NTRK2, PAFAH1B1, PDCD4, PHLPP1, PKHD1, PKIA, PLAC1, PLCB1, PLCH1, POU1F1, PRKD1, PRKG1, PRKN, PTGER3, PTPN11, QKI, RAB3C, RAD23A, RAG2, RASGRF1, REST, RGS12, RPL8, RXRA, SCRIB, SEMA4A, SHROOM3, SMARCD2, SORCS3, SPAG9, ST3GAL5, ST8SIA5, STAT4, STC1, SUCLG1, SV2C, TBP, TFAP2A, THRB, TJP2, TMED2, TNFRSF8, TPP2, TXNDC5, UBA6, USP33, UTRN, VAMP4, WNT2, YY1, ZEB1*
GBA	enzyme	2.54 x 10^{-6}	*ATF3, CD46, CD80, CEBPA, CEBPB, CXCL9, DBP, ETS1, FOS, ICAM1, JUN, JUNB, KLF15, LEP, MYC, NFATC2, NR3C1, POU1F1, PRDM1, RBP1, SORCS3, TBP*

PDE6G	enzyme	2.56 x 10^{-6}	*ABCA4, ALDH4A1, ANKH, APBB2, ARHGEF9, ATF1, ATF3, ATP6V0D2, ATP6V1H, ATXN1, BCAR3, BMP6, BRF2, CACNB2, CAST, CDC42BPB, CHD9, CHI3L2, CHMP4A, CIT, CLEC7A, CLSTN2, CNOT4, COL14A1, DBP, DGKI, DHX9, EIF2B1, EPHA3, ERAP1, ETS1, ETS2, EYA4, F2RL3, FAM171A1, FAM208A, FHIT, FMN1, FOS, FOSL1, FOXJ1, FOXO4, FOXP3, GABRA1, GATA1, GH1, GLCCI1, GNAI2, GNAQ, GRAP2, GRIK2, HDAC9, HIP1, HNF1A, HNF1B, HNF4A, ICAM3, IL1R2, IQGAP1, IVNS1ABP, JUN, JUNB, JUND, KPNA3, LNPEP, MAGEA11, MDGA1, MIGA1, MIOX, NEDD8, NEK1, NKX2-1, NLGN1, NLGN4X, NTRK2, PAFAH1B1, PDCD4, PHLPP1, PKHD1, PKIA, PLAC1, PLCB1, PLCH1, POU1F1, PRKD1, PRKG1, PRKN, PTGER3, PTPN11, QKI, RAB3C, RAD23A, RAG2, RASGRF1, REST, RGS12, RPL8, RXRA, SCRIB, SEMA4A, SHROOM3, SMARCD2, SORCS3, SPAG9, ST3GAL5, ST8SIA5, STAT4, STC1, SUCLG1, SV2C, TBP, TFAP2A, THRB, TJP2, TMED2, TNFRSF8, TPP2, TXNDC5, UBA6, USP33, UTRN, VAMP4, WNT2, YY1, ZEB1*
IGF2BP2	translation regulator	2.56 x 10^{-6}	*ABCA4, ALDH4A1, ANKH, APBB2, ARHGEF9, ATF1, ATF3, ATP6V0D2, ATP6V1H, ATXN1, BCAR3, BMP6, BRF2, CACNB2, CAST, CDC42BPB, CHD9, CHI3L2, CHMP4A, CIT, CLEC7A, CLSTN2, CNOT4, COL14A1, DBP, DGKI, DHX9, EIF2B1, EPHA3, ERAP1, ETS1, ETS2, EYA4, F2RL3, FAM171A1, FAM208A, FHIT, FMN1, FOS, FOSL1, FOXJ1, FOXO4, FOXP3, GABRA1, GATA1, GH1, GLCCI1, GNAI2, GNAQ, GRAP2, GRIK2, HDAC9, HIP1, HNF1A, HNF1B, HNF4A, ICAM3, IL1R2, IQGAP1,*

			IVNS1ABP, JUN, JUNB, JUND, KPNA3, LNPEP, MAGEA11, MDGA1, MIGA1, MIOX, NEDD8, NEK1, NKX2-1, NLGN1, NLGN4X, NTRK2, PAFAH1B1, PDCD4, PHLPP1, PKHD1, PKIA, PLAC1, PLCB1, PLCH1, POU1F1, PRKD1, PRKG1, PRKN, PTGER3, PTPN11, QKI, RAB3C, RAD23A, RAG2, RASGRF1, REST, RGS12, RPL8, RXRA, SCRIB, SEMA4A, SHROOM3, SMARCD2, SORCS3, SPAG9, ST3GAL5, ST8SIA5, STAT4, STC1, SUCLG1, SV2C, TBP, TFAP2A, THRB, TJP2, TMED2, TNFRSF8, TPP2, TXNDC5, UBA6, USP33, UTRN, VAMP4, WNT2, YY1, ZEB1
PI3K p85	group	2.59×10^{-6}	*ANKH, CEBPB, CXCL9, ELK1, ETS1, FHIT, FOSL1, FOXM1, FOXO4, FOXP3, GATA2, HES1, ICAM1, JUN, JUND, KLF15, LEP, MYC, PGR, RAG1, SELP, SP1, TGFB2, UTRN, WT1, ZEB1*
SK & F 86002	chemical - kinase inhibitor	2.61×10^{-6}	*CD46, CD80, CEBPA, CEBPB, CLEC7A, CXCL9, DAB1, DBP, ETS1, FOS, FOSL1, FOXJ1, GATA1, GBX1, HES1, ICAM1, JUN, JUNB, KLF15, LCP1, LEP, MFAP3, MYC, NFATC2, NR3C1, PRDM1, RBP1, SORCS3, TBP, TGFB2, TNFRSF8, VAMP4*
PRNP	other	2.63×10^{-6}	*AHR, ANKH, ATF6, ATP6V0D2, ATP6V1H, BCL11B, CALR, CD46, CD80, CLEC7A, CREM, CXCL9, DBP, ELK1, ERAP1, ETS1, ETS2, FOS, FOSL1, FOXA2, FOXP3, GATA2, GRIA4, HDAC9, HES1, HIP1, HNF1A, ICAM1, IRF1, JUN, JUNB, JUND, KLF15, LIMD2, MTMR7, MYC, NFATC2, NKX2-1, NOD1, NTRK2, POU1F1, PRDM1, RAB3C, RAD23A, RBP1, SORCS3, STAT4, TBP, TGFB2, WT1, ZEB1*

PRMT2	enzyme	2.66 x 10^{-6}	*ABCA4, AHR, ARHGEF9, ATF3, ATP6V1H, CD80, CDC42BPB, CEBPA, CEBPB, CHMP4A, CIT, CLEC7A, CNOT4, CREM, CXCL9, DBP, ELK1, ERAP1, ETS1, FMN1, FOXM1, FOXO4, FOXP3, GATA1, GNAQ, HDAC9, HIP1, ICAM1, IQGAP1, IRF1, JUN, JUNB, JUND, KPNA3, LEP, MAP3K3, NEK1, NLGN1, NLGN4X, PDCD4, PGR, PLCB1, PRDM1, PTGER3, RAB3C, RBL2, ROBO1, SP1, SPAG9, STAT4, TCF7L2, TGFB2, THRB, TJP2, TPP2*
TPCA-1	chemical - kinase inhibitor	2.67 x 10^{-6}	*ACTG1, AGA, ALDH4A1, APBB2, APPL2, ATF3, ATP6V0D2, BCAR3, BRF2, CAMK2D, CD46, CD80, CEBPA, CEBPB, CHI3L2, CIT, CLEC7A, COL14A1, CXCL9, DAB1, EIF2B1, ERAP1, ETS1, ETS2, FHIT, FOS, FOXA1, FOXJ1, FOXM1, FOXO4, FOXP3, GADD45GIP1, GATA1, GBX1, GLCCI1, GNAI2, GRIA4, HNF1A, HNF4A, HOXA5, ICAM1, ICAM3, ICAM5, IL1R2, IRF1, JUN, JUNB, KCND2, KLF15, KPNA3, LCP1, LEP, LIMD2, LRP2, LUC7L3, MED12L, MFAP3, MTMR7, NEDD8, NFATC2, NKX2-1, NR3C1, NTRK2, PAX5, PDCD4, PGR, PHLPP1, PIK3R1, POLD3, POU1F1, PPAT, PRDM1, PRKD1, PRKN, PTGER3, PTPN11, QKI, RAG1, RBP1, RGS12, ROBO1, RPE65, RPL8, RXRA, SCRIB, SHROOM3, SMARCD2, SORCS3, SP1, ST8SIA5, STC1, SUCLG1, SYNE1, TCF7L2, TFAP2A, THRB, TIAM1, TNFRSF8, UPK1B, UTRN, VAMP4, VCL, VTI1A, WNT2, WT1, YY1*

NFkB1-CRel	complex	2.68 x 10^{-6}	*ACTG1, AGA, AHR, ALDH4A1, APBB2, ATF1, ATP6V0D2, ATXN1, BCL11B, BMP6, BRF2, CAMK2D, CD46, CD80, CEBPA, CHI3L2, COL14A1, CREM, CXCL9, DBP, DGKI, EIF2B1, EPHA3, ERAP1, FOS, FOSL1, GATA1, GATA2, GNAQ, GRIK2, HDAC9, HIP1, ICAM1, ICAM5, IL1R2, JUN, JUND, KCND2, KIR3DL1, KPNA3, MDGA1, MED12L, MIGA1, MYC, MYO10, NEDD8, NKX2-1, NR3C1, PAX5, PDCD4, PFKP, PGR, PHLPP1, PIK3R1, PPAT, PPP6C, PRDM1, PRKD1, PRKN, PSMA2, PTPN11, QKI, RAD23A, RAG2, RGS12, RPL8, RXRA, SCRIB, SELP, SEMA3E, SEMA4A, SHROOM3, SMARCD2, SORCS3, SP1, ST8SIA5, STC1, SUCLG1, SV2C, TBP, TCF7L2, TGFB2, TJP2, TNFRSF8, VCL, VTI1A, WNT2, WT1*
mir-221	microRNA	2.71 x 10^{-6}	*CEBPB, ELK1, ETS1, FHIT, FOSL1, FOXM1, GATA2, HES1, IRF1, JUN, JUNB, LEP, MYC, PGR, PIK3R1, RAG1, TFAP2A, UTRN, WT1, ZEB1*
hemin	chemical - endogenous mammalian	2.72 x 10^{-6}	*AHR, API5, ATF6, BCL11B, BMP6, CD46, CEBPA, DBP, ELK1, EPHA3, ETS2, FHIT, FOXM1, FOXO4, FOXP3, GATA2, HDAC9, HES1, HNF4A, JUN, JUNB, JUND, KLF15, MAPKAP1, MIOX, NEK1, NFATC2, NR3C1, PDCD4, PGR, PKIA, POU1F1, PRDM1, PRKG1, RAG1, RBP1, RXRA, SORCS3, SP1, STC1, TBP, TFAP2A, TGFB2, UTRN, WT1, ZEB1*
NCOR2	transcription regulator	2.74 x 10^{-6}	*ABCA4, ACER1, AHR, ARHGEF9, ATF3, ATP6V1H, ATP7B, ATXN1, CAST, CD80, CDC42BPB, CEBPA, CHD9, CHMP4A, CLEC7A, CNOT4, ELK1, EP400, EPHA3, ETS2, EYA4, FHIT, FMN1, FOXA1, FOXA2, FOXM1, FOXO4, GABRA1, GATA1, GATA2, GGCT, GH1, GLCCI1, GNAQ, HNF1B, HNF4A,*

			IQGAP1, JUN, JUNB, JUND, KPNA3, LEP, LNPEP, MAP3K3, MYC, MYO10, NEK1, NLGN1, NLGN4X, NR3C1, PDCD4, PFKP, PGR, PHLPP1, PLAC1, PLCB1, PPM1H, PSMA2, PTGER3, RAG1, RBL2, ROBO1, RPE65, SELP, SPAG9, STAT4, STC1, TCF7L2, TFAP2A, THRB, TJP2, TNFRSF8, TPP2, UTRN, VCL, WNT2, WT1, ZEB1
MIR124	group	2.78×10^{-6}	*AHR, CD46, CD80, CEBPA, CEBPB, CXCL9, FOS, FOXA2, FOXM1, FOXP3, GRIA4, HNF1A, HNF1B, HNF4A, ICAM1, IQGAP1, JUNB, LEP, LRP2, MYC, NFATC2, PAX5, PGR, PRDM1, REST, RPE65, UTRN*
SKAP1	kinase	2.87×10^{-6}	*ABCA4, ALDH4A1, ANKH, APBB2, ARHGEF9, ATF1, ATF3, ATP6V0D2, ATP6V1H, ATXN1, BCAR3, BMP6, BRF2, CACNB2, CAST, CDC42BPB, CHD9, CHI3L2, CHMP4A, CIT, CLEC7A, CLSTN2, CNOT4, COL14A1, DBP, DGKI, DHX9, EIF2B1, EPHA3, ERAP1, ETS1, ETS2, EYA4, F2RL3, FAM171A1, FAM208A, FHIT, FMN1, FOS, FOSL1, FOXJ1, FOXO4, FOXP3, GABRA1, GATA1, GH1, GLCCI1, GNAI2, GNAQ, GRAP2, GRIK2, HDAC9, HIP1, HNF1A, HNF1B, HNF4A, ICAM3, IL1R2, IQGAP1, IVNS1ABP, JUN, JUNB, JUND, KPNA3, LNPEP, MAGEA11, MDGA1, MIGA1, MIOX, NEDD8, NEK1, NKX2-1, NLGN1, NLGN4X, NTRK2, PAFAH1B1, PDCD4, PHLPP1, PKHD1, PKIA, PLAC1, PLCB1, PLCH1, POU1F1, PRKD1, PRKG1, PRKN, PTGER3, PTPN11, QKI, RAB3C, RAD23A, RAG2, RASGRF1, REST, RGS12, RPL8, RXRA, SCRIB, SEMA4A, SHROOM3, SMARCD2, SORCS3, SPAG9, ST3GAL5, ST8SIA5, STAT4, STC1, SUCLG1, SV2C, TBP, TFAP2A, THRB, TJP2, TMED2, TNFRSF8, TPP2, TXNDC5,*

			UBA6, USP33, UTRN, VAMP4, WNT2, YY1, ZEB1
ADRA1	group	2.94×10^{-6}	*ATF3, ATF6, BCAR3, BCL11B, CALR, CD80, CEBPA, DBP, ETS1, FHIT, FOS, FOSL1, FOXA2, FOXO4, FOXP3, GATA2, HES1, ICAM1, ICAM3, JUN, JUNB, JUND, NR3C1, PAFAH1B1, PAX5, PDCD4, PGR, POU1F1, PRDM1, RAG1, RBP1, TBP, UPK1B, UTRN, WT1, ZEB1*
benzyloxycarbonyl-Leu-Leu-Leu aldehyde	chemical - protease inhibitor	3.01×10^{-6}	*ABL1, AHR, ATF3, CEBPA, CEBPB, ETS1, FOS, FOXM1, HNF1A, ICAM1, JUN, MYC, NEDD8, NKX2-1, NR3C1, PDCD4, PGR, PRDM1, RXRA, SCRIB*
ITGA2	transmembrane receptor	3.15×10^{-6}	*ATF3, BCAR3, CALR, CD46, CD80, CEBPA, CEBPB, CREM, CXCL9, ETS1, FOS, FOXA2, FOXP3, HNF4A, ICAM1, ICAM3, IRF1, JUNB, JUND, KLF15, LEP, MYC, NFATC2, PGR, POU1F1, PRDM1, PRKG1, RBL2, RBP1, SELP, SORCS3, TBP, TIAM1, VCL, ZEB1*
NFAT (complex)	complex	3.16×10^{-6}	*ABCA4, ACER1, AHR, ALDH4A1, APBB2, ARHGEF9, ATF3, ATP6V0D2, ATP6V1H, ATXN1, CALR, CDC42BPB, CEBPB, CHMP4A, CIT, CLEC7A, CLSTN2, CNOT4, COL14A1, DBP, EIF2B1, ELK1, EPHA3, ETS1, EYA4, FHIT, FMN1, FOS, FOXA1, FOXA2, FOXO4, FOXP3, GGCT, GNAQ, HDAC9, HES1, HNF1A, HNF1B, HNF4A, ICAM1, IQGAP1, IRF1, JUN, JUND, KCND2, KPNA3, LEP, MAP3K3, MYC, NEDD8, NEK1, NLGN1, NLGN4X, PAX5, PFKP, PLAC1, PLCB1, PRDM1, PRKD1, PRKG1, PRKN, PTGER3, PTPN11, PTPRS, RAB10, RAD23A, RAG2, RGS12, RXRA, SCRIB, SELP, SHROOM3, SP1, SPAG9, SUCLG1, TBP, TCF7L2, THRB, TJP2, TPP2, UTRN, VCL, WNT2, WT1*

CD3D	transmembrane receptor	3.19 x 10^{-6}	*ABCA4, ALDH4A1, APBB2, ARHGEF9, ATF1, ATF3, ATF6, ATP6V0D2, ATP6V1H, ATXN1, BCAR3, BCL11B, BRF2, CACNB2, CALR, CD46, CDC42BPB, CHD9, CHI3L2, CHMP4A, CIT, CLEC7A, CLSTN2, CNOT4, COL14A1, CSRP2, DBP, DGKI, DHX9, EIF2B1, ERAP1, ETS2, FAM171A1, FAM208A, FHIT, FMN1, FOS, FOXJ1, FOXO4, GABRA1, GATA1, GLCCI1, GNAI2, GNAQ, GRAP2, GRIK2, HDAC9, HIP1, HNF1A, HNF1B, HNF4A, ICAM3, IL1R2, IQGAP1, IRF2, IVNS1ABP, JUN, JUNB, JUND, KLF15, KPNA3, LNPEP, LRP2, MAGEA11, MDGA1, MIGA1, MIOX, NEDD8, NEK1, NKX2-1, NLGN1, NLGN4X, NR3C1, NTRK2, PAFAH1B1, PHLPP1, PIK3R1, PKHD1, PKIA, PLAC1, PLCB1, PLCH1, POU1F1, PRKD1, PRKG1, PRKN, PTGER3, PTPN11, PTPRS, QKI, RAB3C, RAD23A, RAG2, RASGRF1, REST, RGS12, RPE65, RPL8, SCRIB, SELP, SEMA4A, SHROOM3, SMARCD2, SORCS3, SPAG9, ST3GAL5, ST8SIA5, STAT4, STC1, SUCLG1, TBP, TFAP2A, THRB, TJP2, TMED2, TNFRSF8, TPP2, UBA6, USP33, UTRN, WNT2, YY1, ZEB1*
TCF7L1	transcription regulator	3.22 x 10^{-6}	*ATF3, CEBPA, CEBPB, CXCL9, ELK1, ETS1, FHIT, FOSL1, FOXA2, FOXM1, FOXO4, GATA2, HES1, JUN, LEP, MYC, PGR, RAG1, TFAP2A, UTRN, WT1, ZEB1*
MSTN	growth factor	3.24 x 10^{-6}	*ATF3, BMP6, CD46, CD80, CEBPA, CEBPB, CXCL9, ELK1, FHIT, FOSL1, FOXM1, FOXP3, GATA2, HES1, HNF4A, ICAM1, JUN, JUNB, JUND, KLF15, LEP, NFATC2, PGR, PRDM1, RAG1, RBP1, RXRA, SORCS3, TBP, TGFB2, UTRN, WT1, ZEB1*
NFAT (complex)	complex	3.25 x 10^{-6}	*AHR, ATF3, FOS, FOXA1, FOXA2, FOXP3, HNF1A, HNF1B, HNF4A, ICAM1, IRF1, JUN,*

			KCND2, MYC, PRDM1, PTPRS, RAB10, UTRN
N-(2-guanidinoethyl)-5-isoquinolinesulfonamide	chemical - kinase inhibitor	3.36 x 10^{-6}	*ADGRL3, AHR, ATF3, CEBPB, CREM, FOSL1, FOXA1, FOXA2, HNF1B, HNF4A, IRF1, JUNB, LEP, LRP2, MYC, PKHD1, POU1F1, VCL, WT1*
SPAG9	other	3.38 x 10^{-6}	*ATF3, BCAR3, CD46, CD80, CEBPA, CEBPB, CXCL9, FOS, FOSL1, FOXK1, FOXP3, HES1, HNF4A, ICAM1, ICAM3, JUN, JUNB, JUND, KLF15, LEP, MYC, NFATC2, PRDM1, RBP1, RXRA, SORCS3, TBP*
RelA-CRel	complex	3.41 x 10^{-6}	*AGA, AHR, ATF1, ATF3, ATXN1, BMP6, BRF2, CAMK2D, CD46, CD80, CEBPB, CHI3L2, COL14A1, CREM, CXCL9, DBP, EPB41L5, ERAP1, FHIT, FOS, FOXA1, FOXO4, GATA2, GLCCI1, GNAQ, GRIA4, GRIK2, HIP1, HNF1B, HNF4A, ICAM1, IRF1, JUN, JUNB, JUND, KCND2, KIR3DL1, KPNA3, MDGA1, MED12L, MIGA1, MYC, NKX2-1, NR3C1, PAX5, PDCD4, PGR, PHLPP1, PIK3R1, PPP6C, PRDM1, RAB3C, RAG1, RAG2, RASGRF1, ROBO1, RPE65, RPL8, RXRA, SELP, SEMA4A, SORCS3, STC1, SV2C, TBP, TFAP2A, TNFRSF8, UTRN, VCL, VTI1A, WT1, ZEB1*
PITX1	transcription regulator	3.48 x 10^{-6}	*ABCA4, ACER1, ARHGEF9, ATF3, ATP6V1H, ATXN1, BCAR3, CDC42BPB, CEBPB, CHMP4A, CIT, CLEC7A, CNOT4, EP400, EPHA3, ETS1, EYA4, FMN1, FOS, FOSL1, FOXA1, FOXO4, GABRA1, GGCT, GLCCI1, GNAQ, GRIA4, HES1, ICAM3, IQGAP1, IRF1, JUND, KPNA3, LEP, MAP3K3, MYC, MYO10, NEK1, NLGN1, NLGN4X, PDCD4, PFKP, PLAC1, PLCB1, PPM1H, PRDM1, PTGER3, RBL2, ROBO1, SP1, SPAG9, STC1, TBP, TCF7L2, TGFB2, THRB, TJP2, TPP2, VCL*

PDE4B	enzyme	3.55 x 10^{-6}	*CEBPB, ELK1, ETS1, FHIT, FOS, FOSL1, FOXM1, GATA2, HES1, ICAM1, JUN, LEP, MYC, PGR, RAG1, SELP, UTRN, WT1, ZEB1*
TNFRSF11A	transmembrane receptor	3.56 x 10^{-6}	*AHR, ATF3, BCAR3, BRF2, CD46, CD80, CEBPA, CEBPB, CHI3L2, CREM, CXCL9, DBP, ERAP1, FHIT, FOSL1, FOXO4, FOXP3, GATA2, HDAC9, HES1, HIP1, HNF4A, ICAM1, ICAM3, IRF1, JUN, JUNB, JUND, KLF15, LEP, NFATC2, NR3C1, PGR, PIK3R1, PRDM1, RAB3C, RAG1, RPL8, RXRA, SELP, SORCS3, STAT4, TBP, TFAP2A, TNFRSF8, UTRN, WT1, ZEB1*
RALGDS	other	3.87 x 10^{-6}	*ATF3, CD80, CEBPB, ELK1, FHIT, FOSL1, FOXM1, FOXO4, GATA2, HES1, HNF4A, ICAM1, JUN, JUND, LEP, MYC, PGR, RAG1, RBP1, RXRA, TBP, UTRN, WT1, ZEB1*
5-oxo-$6^{-8\ -11-14}$-(e,z,z,z)-eicosatetraenoic acid	chemical - endogenous mammalian	3.89 x 10^{-6}	*ABCA4, ABLIM3, AFAP1, AHR, ALDH4A1, APBB2, ARHGEF9, ARID5B, ATF1, ATF3, ATF6, ATP6V0D2, ATP6V1H, ATXN1, BRF2, CACNB2, CALR, CD46, CD80, CDC42BPB, CHI3L2, CHMP4A, CIT, CLSTN2, CNOT4, CNTNAP2, COL14A1, CSRP2, CXCL9, DAB1, DBP, DGKI, DHX9, EIF2B1, ELK1, EP400, ETS1, F2RL3, FAM171A1, FHIT, FMN1, FOS, FOSL1, FOXA1, FOXJ1, FOXO4, GADD45GIP1, GATA1, GBX1, GMPR, GNAQ, HDAC9, HES1, HIP1, HNF1A, HNF1B, ICAM1, ICAM5, IL1R2, IQGAP1, IVNS1ABP, JUN, JUNB, JUND, KCNN2, KLF15, KPNA3, LARGE1, LCP1, LEP, LIMD2, LUC7L3, MAGEA11, MAP3K3, MAPKAP1, MDGA1, MFAP3, MIGA1, MOG, MSS51, MTMR7, MYC, MYO10, NEDD8, NEK1, NFATC2, NFIA, NLGN1, NLGN4X, NOD1, NSG1, PAFAH1B1, PAICS, PKHD1, PKIA, PLAC1, PLCB1, PLCH1, PPM1H, PRDM1, PRKD1, PRKN, PSMA2, PTGER3, PTPN11, PTPRS,*

			RAB10, RAB3C, RAD23A, RAG2, RASGRF1, RBP1, RGS12, RPL8, SCRIB, SHROOM3, SLC38A3, SMARCD2, SORCS3, SPAG9, ST3GAL5, ST8SIA5, STC1, SUCLG1, SV2C, TBP, TFAP2A, THRB, TJP2, TMED2, TNFRSF8, UBA6, UPK1B, USP33, UTRN, VAMP4, WNT2, WWOX, YY1, ZEB1, ZNF148
LDL-cholesterol	complex	3.89×10^{-6}	*ATF3, CD46, CD80, CEBPB, CXCL9, ETS1, FOS, FOSL1, FOXA2, GATA2, HES1, HNF1A, ICAM1, JUN, JUNB, KLF15, LEP, LIMD2, MTMR7, MYC, NFATC2, NKX2-1, PRDM1, RAD23A, RBP1, SORCS3, TBP, WT1, ZEB1*
TRAF1-TRAF2-TRAF3	complex	3.91×10^{-6}	*AHR, ANKH, ATF3, CD46, CD80, CEBPA, CEBPB, CREM, CXCL9, DBP, ELK1, ERAP1, FOS, FOSL1, GH1, HDAC9, HES1, HIP1, HNF4A, ICAM1, IRF1, JUN, JUNB, JUND, KLF15, LEP, MYC, NFATC2, PRDM1, RAB3C, RBP1, RXRA, SORCS3, STAT4, TBP*
ZNF622	other	3.91×10^{-6}	*ATF3, CD46, CD80, CEBPB, CXCL9, FOS, FOSL1, GATA2, HES1, HNF4A, ICAM1, JUN, JUNB, JUND, KLF15, LEP, MYC, NFATC2, NR3C1, PRDM1, RBP1, RXRA, SORCS3, TBP, TNFRSF8*
MZB1	other	3.95×10^{-6}	*AHR, ATF3, CD80, CEBPA, ELK1, ETS1, FOS, FOSL1, HES1, HNF4A, ICAM1, JUN, JUND, LEP, MYC, RBP1, RXRA, SP1, TBP*
BTNL2	transmembrane receptor	4.24×10^{-6}	*APPL2, ATP6V0D2, CD109, CEBPB, ELK1, ETS1, FHIT, FOS, FOSL1, FOXA2, FOXM1, FOXP3, GATA1, HES1, JUNB, LEP, MYC, MYO10, PAX5, PGR, PHLPP1, PRDM1, RAG1, RAG2, RBL2, UTRN, WT1, ZEB1*

SIGIRR	transmembrane receptor	4.28×10^{-6}	*ABCA4, ACER1, ACTG1, AFAP1, AGA, AHR, ALDH4A1, APBB2, API5, ARHGEF9, ARID5B, ATF1, ATF3, ATF6, ATP7B, ATXN1, BCAR3, BCL11B, BRF2, CAST, CD276, CD80, CDC42BPB, CEBPA, CEBPB, CHI3L2, CHMP4A, CIT, CLEC7A, CLIP1, CLSTN2, CNOT4, CNTNAP2, CREM, CSRP2, CXCL9, DBP, DGKI, DHX9, EIF2B1, ELK1, EP400, EPB41L5, EPHA3, ERAP1, EYA4, F2RL3, FAM171A1, FEM1B, FMN1, FOS, FOXA1, FOXP3, GABRA1, GATA1, GH1, GMPR, GNAQ, GRIA4, GRIK2, HDAC9, HES1, HIP1, HNF1B, HNF4A, ICAM1, ICAM5, IQGAP1, IRF2, JUN, JUNB, JUND, KCND2, KPNA3, LARGE1, LCP1, LEP, LIMD2, LNPEP, LRP2, LUC7L3, MAP3K3, MAPKAP1, MARCH3, MED12L, MOG, MRPL13, MSS51, MTMR7, MYC, NEDD8, NEK1, NLGN1, NLGN4X, NOD1, NR3C1, NSG1, NTRK2, PAFAH1B1, PAICS, PFKP, PGR, PHLPP1, PIK3R1, PKIA, PLCB1, PLCB4, PPAT, PRDM1, PRKD1, PRKN, PSMA2, PTGER3, PTPN11, RAB3C, RBP1, RGS12, ROBO1, RPL8, RXRA, SBF1, SCRIB, SELP, SHROOM3, SIPA1L3, SLC38A3, SMARCD2, SNTB1, SPAG9, ST3GAL5, ST8SIA5, STAT4, STC1, SV2C, SYNE1, TBP, TFAP2A, THRB, TIAM1, TICRR, TJP2, TMED2, TNFRSF8, TUBGCP2, TXNDC5, USP33, VCL, VTI1A, WNT2, WWOX, YY1, ZNF148*
Nfat (family)	group	4.28×10^{-6}	*AHR, ATF3, ATXN1, CD276, CD80, CLEC7A, FOSL1, FOXA1, FOXA2, FOXM1, FOXP3, GADD45GIP1, GATA1, HNF1A, HNF1B, HNF4A, ICAM1, IRF1, JUN, KCND2, LCP1, LEP, MYC, NFATC2, PRDM1, PTPRS, RAB10, SNTB1, TGFB2, TICRR, UTRN, ZEB1*

WNT5A	cytokine	4.32×10^{-6}	*AHR, ATF1, ATF6, BCAR3, BCL11B, BMP6, CALR, CD46, CD80, CEBPB, CREM, CXCL9, DBP, EPHA3, ERAP1, FHIT, FOSL1, FOXA2, FOXM1, FOXO4, GABRA1, GATA2, HDAC9, HIP1, HNF1A, HNF1B, ICAM3, IRF1, IRF2, JUND, KLF15, LCP1, LEP, LIMD2, LUC7L3, MAGEA11, MTMR7, MYC, NFATC2, NR3C1, POU1F1, PRDM1, PRKG1, PTPRS, RAB10, RAB3C, RAD23A, RAG1, RBP1, RXRA, SORCS3, SP1, STAT4, STC1, TGFB2, UTRN, ZEB1*
1,2-dipalmitoyl-sn-glycero-3-phosphate	chemical - endogenous mammalian	4.33×10^{-6}	*AHR, ANKH, ATF6, ATP6V0D2, ATP6V1H, BCL11B, CD80, DBP, ELK1, ETS1, FHIT, FOSL1, FOXM1, FOXP3, GATA2, GRIK2, HES1, HNF4A, ICAM1, IRF1, JUN, JUNB, JUND, KCND2, KLF15, LEP, MOG, MRPL13, PDCD4, PFKP, PGR, POU1F1, PRKG1, PSMA2, RAG1, RBL2, RPL8, SLC38A3, TCF7L2, TGFB2, UTRN, WT1, ZEB1*
GADD45G	other	4.45×10^{-6}	*AFAP1, ATF3, CD46, CD80, CEBPA, CEBPB, CXCL9, DBP, ELK1, FOSL1, FOXM1, FOXO4, HES1, HNF4A, ICAM1, IRF1, JUN, JUNB, JUND, KLF15, LEP, NFATC2, NR3C1, PGR, POU1F1, PRDM1, QKI, RAG1, RBL2, RBP1, RXRA, SORCS3, TBP, TIAM1, TPP2, VAMP4, WWOX, ZNF148*
mir^{-21} 8	microRNA	4.49×10^{-6}	*CEBPB, DBP, FOS, JUN, JUNB, LEP, MDGA1, MYC, NR3C1, POU1F1, ZEB1*
TNFRSF10B	transmembrane receptor	4.58×10^{-6}	*AHR, ATF3, CD46, CD80, CEBPA, CEBPB, CREM, CXCL9, DBP, ERAP1, FHIT, FOSL1, FOXM1, FOXO4, GATA2, HDAC9, HES1, HIP1, HNF4A, ICAM1, IRF1, JUN, JUNB, JUND, KLF15, LEP, NFATC2, NR3C1, PGR, POU1F1, PRDM1, RAB3C, RAG1, RBP1, RXRA, SELP, SORCS3, STAT4, TBP, UTRN, WT1, ZEB1*

HACD3	enzyme	4.64×10^{-6}	*ABCA4, ACTG1, AFAP1, AHR, ALDH4A1, APBB2, API5, ARHGEF9, ATF1, ATF3, ATF6, ATP6V0D2, ATP6V1H, ATP7B, ATXN1, BRF2, CAMK2D, CAST, CD276, CD46, CD80, CDC42BPB, CEBPB, CHI3L2, CHMP4A, CLEC7A, CNTNAP2, CREM, CXCL9, DBP, EIF2B1, ELK1, EPB41L5, EPHA3, ERAP1, ETS1, EYA4, F2RL3, FEM1B, FMN1, FOS, FOSL1, FOXA1, FOXO4, GABRA1, GADD45GIP1, GATA1, GH1, GLCCI1, GNAI2, GNAQ, GRIA4, GRIK2, HDAC9, HIP1, ICAM1, ICAM3, ICAM5, IL1R2, IQGAP1, IRF1, IRF2, IVNS1ABP, JUN, JUNB, JUND, KCND2, KLF15, KPNA3, LARGE1, LCP1, LEP, LIMD2, LNPEP, LRP2, LUC7L3, MAP3K3, MRPL13, MSS51, MTMR7, NEDD8, NEK1, NKX2-1, NLGN1, NLGN4X, NR3C1, NTRK2, P2RY12, PAFAH1B1, PAICS, PGR, PHLPP1, PIK3R1, PKIA, PLCB1, POU1F1, PPAT, PRDM1, PRKD1, PRKG1, PRKN, PSMA2, PTGER3, PTPN11, PTPRS, RAB10, RAB3C, RAD23A, RBP1, RGS12, ROBO1, RPL8, SCRIB, SEMA3E, SHROOM3, SIPA1L3, SNTB1, SPAG9, STAT4, SUCLG1, TBP, THRB, TIAM1, TICRR, TJP2, TMED2, TNFRSF8, VAMP4, WNT2, YY1, ZNF148*
CGP53716	chemical - kinase inhibitor	4.69×10^{-6}	*ABCA4, ACTG1, AFAP1, AHR, ALDH4A1, APBB2, API5, ARHGEF9, ATF1, ATF3, ATF6, ATP6V0D2, ATP6V1H, ATP7B, ATXN1, BRF2, CAMK2D, CAST, CD276, CD46, CD80, CDC42BPB, CEBPB, CHI3L2, CHMP4A, CLEC7A, CNTNAP2, CREM, CXCL9, DBP, EIF2B1, ELK1, EPB41L5, EPHA3, ERAP1, ETS1, EYA4, F2RL3, FEM1B, FMN1, FOS, FOSL1, FOXA1, FOXO4, GABRA1, GADD45GIP1, GATA1, GH1, GLCCI1, GNAI2, GNAQ, GRIA4, GRIK2, HDAC9, HIP1, ICAM1, ICAM3, ICAM5, IL1R2, IQGAP1,*

			IRF1, IRF2, IVNS1ABP, JUN, JUNB, JUND, KCND2, KLF15, KPNA3, LARGE1, LCP1, LEP, LIMD2, LNPEP, LRP2, LUC7L3, MAP3K3, MRPL13, MSS51, MTMR7, NEDD8, NEK1, NKX2-1, NLGN1, NLGN4X, NR3C1, NTRK2, P2RY12, PAFAH1B1, PAICS, PGR, PHLPP1, PIK3R1, PKIA, PLCB1, POU1F1, PPAT, PRDM1, PRKD1, PRKG1, PRKN, PSMA2, PTGER3, PTPN11, PTPRS, RAB10, RAB3C, RAD23A, RBP1, RGS12, ROBO1, RPL8, SCRIB, SEMA3E, SHROOM3, SIPA1L3, SNTB1, SPAG9, STAT4, SUCLG1, TBP, THRB, TIAM1, TICRR, TJP2, TMED2, TNFRSF8, VAMP4, WNT2, YY1, ZNF148
JDP2	transcription regulator	4.8×10^{-6}	*ATF3, ATXN1, CALR, CD46, CD80, CEBPA, CEBPB, CXCL9, EP400, ETS1, FOS, GABRA1, HES1, ICAM1, JUN, JUNB, JUND, KLF15, LEP, MYC, MYO10, NFATC2, PFKP, PPM1H, PRDM1, RBP1, SORCS3, STC1, TBP, VCL, ZEB1*
DDX17	enzyme	4.84×10^{-6}	*ABCA4, ARHGEF9, ATF3, ATP6V1H, CDC42BPB, CEBPA, CEBPB, CHMP4A, CIT, CLEC7A, CNOT4, FMN1, FOS, FOSL1, FOXM1, FOXO4, FOXP3, GNAQ, ICAM1, IQGAP1, IRF1, JUN, JUNB, JUND, KPNA3, LEP, MAP3K3, MYC, NEK1, NLGN1, NLGN4X, PDCD4, PGR, PLCB1, PTGER3, RBL2, ROBO1, SP1, SPAG9, TCF7L2, TGFB2, THRB, TJP2, TPP2*
serotonin receptor	group	4.87×10^{-6}	*ABCA4, ALDH4A1, ANKH, APBB2, API5, ARHGEF9, ATF3, ATF6, ATP6V0D2, ATP6V1H, ATXN1, BCAR3, BMP6, CACNB2, CALR, CD46, CDC42BPB, CHD9, CHMP4A, CIT, CLEC7A, CLSTN2, CNOT4, COL14A1, DBP, DGKI, DHX9, EIF2B1, EPHA3, ERAP1, ETS1, ETS2, FAM171A1, FAM208A, FHIT, FMN1, FOS, FOXJ1, FOXO4, FOXP3,*

			GABRA1, GATA1, GH1, GLCCI1, GNAI2, GNAQ, GRAP2, GRIK2, HDAC9, HIP1, HNF1A, HNF1B, HNF4A, ICAM3, IL1R2, IQGAP1, IRF2, IVNS1ABP, JUN, JUNB, JUND, KPNA3, LNPEP, LRP2, MAGEA11, MDGA1, MIGA1, MIOX, NEDD8, NEK1, NKX2-1, NLGN1, NLGN4X, NR3C1, PAFAH1B1, PDCD4, PHLPP1, PIK3R1, PKHD1, PKIA, PLAC1, PLCB1, PLCH1, POU1F1, PRKD1, PRKG1, PRKN, PTGER3, PTPN11, QKI, RAB3C, RAD23A, RAG2, RASGRF1, REST, RGS12, RPE65, SCRIB, SEMA4A, SHROOM3, SMARCD2, SORCS3, SPAG9, ST3GAL5, ST8SIA5, STAT4, STC1, SUCLG1, SV2C, TBP, TFAP2A, THRB, TJP2, TMED2, TNFRSF8, TPP2, UBA6, USP33, UTRN, WNT2, WT1, YY1, ZEB1
RIPK4	kinase	4.92×10^{-6}	*ABCA4, ACTG1, AFAP1, AHR, ALDH4A1, APBB2, API5, ARHGEF9, ATF1, ATF3, ATF6, ATP6V0D2, ATP6V1H, ATP7B, ATXN1, BRF2, CAMK2D, CAST, CD276, CD46, CD80, CDC42BPB, CEBPB, CHI3L2, CHMP4A, CLEC7A, CNTNAP2, CREM, CXCL9, DBP, EIF2B1, ELK1, EPB41L5, EPHA3, ERAP1, ETS1, EYA4, F2RL3, FEM1B, FMN1, FOS, FOSL1, FOXA1, FOXO4, GABRA1, GADD45GIP1, GATA1, GH1, GLCCI1, GNAI2, GNAQ, GRIA4, GRIK2, HDAC9, HIP1, ICAM1, ICAM3, ICAM5, IL1R2, IQGAP1, IRF1, IRF2, IVNS1ABP, JUN, JUNB, JUND, KCND2, KLF15, KPNA3, LARGE1, LCP1, LEP, LIMD2, LNPEP, LRP2, LUC7L3, MAP3K3, MRPL13, MSS51, MTMR7, NEDD8, NEK1, NKX2-1, NLGN1, NLGN4X, NR3C1, NTRK2, P2RY12, PAFAH1B1, PAICS, PGR, PHLPP1, PIK3R1, PKIA, PLCB1, POU1F1, PPAT, PRDM1, PRKD1, PRKG1, PRKN, PSMA2, PTGER3, PTPN11, PTPRS, RAB10, RAB3C,*

			RAD23A, RBP1, RGS12, ROBO1, RPL8, SCRIB, SEMA3E, SHROOM3, SIPA1L3, SNTB1, SPAG9, STAT4, SUCLG1, TBP, THRB, TIAM1, TICRR, TJP2, TMED2, TNFRSF8, VAMP4, WNT2, YY1, ZNF148
ELP1	other	4.92×10^{-6}	*ABCA4, ACTG1, AFAP1, AHR, ALDH4A1, APBB2, API5, ARHGEF9, ATF1, ATF3, ATF6, ATP6V0D2, ATP6V1H, ATP7B, ATXN1, BRF2, CAMK2D, CAST, CD276, CD46, CD80, CDC42BPB, CEBPB, CHI3L2, CHMP4A, CLEC7A, CNTNAP2, CREM, CXCL9, DBP, EIF2B1, ELK1, EPB41L5, EPHA3, ERAP1, ETS1, EYA4, F2RL3, FEM1B, FMN1, FOS, FOSL1, FOXA1, FOXO4, GABRA1, GADD45GIP1, GATA1, GH1, GLCCI1, GNAI2, GNAQ, GRIA4, GRIK2, HDAC9, HIP1, ICAM1, ICAM3, ICAM5, IL1R2, IQGAP1, IRF1, IRF2, IVNS1ABP, JUN, JUNB, JUND, KCND2, KLF15, KPNA3, LARGE1, LCP1, LEP, LIMD2, LNPEP, LRP2, LUC7L3, MAP3K3, MRPL13, MSS51, MTMR7, NEDD8, NEK1, NKX2-1, NLGN1, NLGN4X, NR3C1, NTRK2, P2RY12, PAFAH1B1, PAICS, PGR, PHLPP1, PIK3R1, PKIA, PLCB1, POU1F1, PPAT, PRDM1, PRKD1, PRKG1, PRKN, PSMA2, PTGER3, PTPN11, PTPRS, RAB10, RAB3C, RAD23A, RBP1, RGS12, ROBO1, RPL8, SCRIB, SEMA3E, SHROOM3, SIPA1L3, SNTB1, SPAG9, STAT4, SUCLG1, TBP, THRB, TIAM1, TICRR, TJP2, TMED2, TNFRSF8, VAMP4, WNT2, YY1, ZNF148*

CDKN1C	other	4.94 x 10^{-6}	*ABCA4, ABLIM3, ACTG1, AFAP1, ALDH4A1, APBB2, ARHGEF9, ATF6, ATP6V0D2, ATP6V1H, ATP7B, ATXN1, BEND5, CAMK2D, CDC42BPB, CEBPA, CEBPB, CHD9, CHMP4A, CLEC7A, CLSTN2, CNTNAP2, COL14A1, CREM, CTBP2, EIF2B1, ELK1, EPHA3, ETS1, EYA4, F2RL3, FAM171A1, FMN1, FOS, FOSL1, FOXJ1, FOXM1, FOXO4, GADD45GIP1, GH1, GNAI2, GNAQ, GRIK2, HDAC9, HOXA5, ICAM5, IQGAP1, IVNS1ABP, JUN, JUND, KCND2, KPNA3, LEP, LIMD2, LUC7L3, MAGEA11, MAP3K3, MRPL13, MTMR7, MYO10, NEDD8, NEK1, NKX2-1, NLGN1, NLGN4X, NTNG1, P2RY12, PAFAH1B1, PAICS, PARD3, PGR, PIK3R1, PKIA, PLAC1, PLCB1, POU1F1, PPAT, PPM1H, PRKD1, PRKN, PSMA2, PTPN11, PTPRS, QKI, RAB10, RAD23A, RAG1, RBL2, RBP1, REST, RGS12, ROBO1, RPL8, SBF1, SCRIB, SEMA4A, SGCD, SHROOM3, SIPA1L3, SORCS3, SPAG9, ST3GAL5, STC1, SV2C, SYNE1, TBP, TGFB2, TIAM1, TJP2, TMED2, TNFRSF8, TPP2, WWOX, YY1, ZEB1, ZFP57, ZNF148, ZNF804A*
LAMC1	other	4.98 x 10^{-6}	*AHR, ATF3, ATF6, BCL11B, CALR, CD80, CEBPA, CEBPB, DBP, ELK1, ETS1, ETS2, FOS, FOSL1, ICAM1, JUN, JUNB, JUND, MYC, POU1F1, SP1, TBP, TGFB2, ZEB1*
UBE2M	enzyme	4.99 x 10^{-6}	*ABCA4, ARHGEF9, ATF3, ATP6V1H, CD80, CDC42BPB, CEBPA, CEBPB, CHMP4A, CIT, CLEC7A, CNOT4, CXCL9, FMN1, FOS, FOXM1, FOXO4, FOXP3, GNAQ, GRIA4, IQGAP1, IRF1, JUN, JUNB, JUND, KPNA3, LEP, MAP3K3, NEK1, NFATC2, NLGN1, NLGN4X, PAX5, PDCD4, PGR, PLCB1, PTGER3, RAG2, RBL2, ROBO1, SELP, SP1,*

			SPAG9, TCF7L2, TGFB2, THRB, TJP2, TPP2, WT1
ammonia	chemical - endogenous mammalian	5.28 x 10^{-6}	*ABCA4, ABL1, ACER1, AFAP1, AGA, ANKH, ANXA7, API5, ARHGEF9, ATF1, ATF3, ATF6, ATP6V0D2, ATP6V1H, ATXN1, BCAR3, BCL11B, BMP6, BRF2, CAMK2D, CAST, CD80, CDC42BPB, CHI3L2, CHMP4A, CIT, CLEC7A, CLIP1, CNOT4, COL14A1, CSRP2, DAB1, DBP, DGKI, DHX9, EPHA3, ETS2, EYA4, F2RL3, FAM171A1, FEM1B, FHIT, FMN1, FOS, FOXA2, GABRA1, GATA1, GLCCI1, GMPR, GNAI2, GNAQ, GRIA4, GRIK2, HDAC9, HIP1, HNF1A, HNF1B, ICAM1, ICAM3, ICAM5, IQGAP1, IRF1, IRF2, IVNS1ABP, KCNIP4, KLF15, KLHL1, KPNA3, LARGE1, LCP1, LIMD2, LRP2, LUC7L3, MAP3K3, MARCH3, MDGA1, MED12L, MIGA1, MOG, MTMR7, MYC, MYO10, NEK1, NKX2-1, NLGN1, NLGN4X, NOD1, NR3C1, NTRK2, PAICS, PFKP, PGR, PLCB1, PLCB4, POLD3, POU1F1, PPAT, PPM1H, PRKG1, PTGER3, PTPN11, PTPRS, QKI, RAB3C, RAG1, RASGRF1, RPE65, RPL8, SBF1, SELP, SEMA3E, SEMA4A, SORCS3, SPAG9, STAT4, STC1, SUCLG1, SV2C, SYNE1, TCF7L2, THRB, TNFRSF8, TPP2, TUBGCP2, TXNDC5, USP33, UTRN, VAMP4, VTI1A, WWOX, YY1, ZEB1, ZFP57, ZNF148*
AHR	ligand-dependent nuclear receptor	5.34 x 10^{-6}	*ABCA4, ARHGEF9, ATF3, ATF6, ATP6V1H, CALR, CD46, CD80, CDC42BPB, CEBPA, CHMP4A, CIT, CLEC7A, CNOT4, COL14A1, CREM, CTBP2, CXCL9, EPB41L5, FAM171A1, FHIT, FMN1, FOSL1, FOXA2, FOXM1, FOXO4, FOXP3, GADD45GIP1, GATA2, GMPR, GNAQ, HDAC9, HES1, HNF1A, ICAM3, IQGAP1, IRF1, IVNS1ABP, JUN, JUNB, JUND, KCNN2, KLF15, KPNA3, LEP, MAP3K3, MYC, NEK1, NFATC2, NLGN1,*

			NLGN4X, NR3C1, PDCD4, PGR, PLCB1, POU1F1, PPM1H, PRDM1, PTGER3, QKI, RAG1, RBP1, ROBO1, SORCS3, SP1, SPAG9, TBP, TCF7L2, TFAP2A, TGFB2, THRB, TIAM1, TJP2, TPP2, UTRN, VCL, WT1
PRLHR	G-protein coupled receptor	5.41×10^{-6}	*CEBPA, CEBPB, ELK1, ETS1, FHIT, FOS, FOSL1, FOXM1, FOXP3, GATA2, HES1, IRF1, JUN, KCND2, LEP, MOG, MYC, PFKP, PGR, PHLPP1, RAG1, UTRN, WT1, ZEB1*
NCOR2	transcription regulator	5.82×10^{-6}	*CD80, CEBPA, JUN, LEP, MYC, PGR*
Atrial Natriuretic Peptide	group	5.95×10^{-6}	*AHR, ATF3, ATP6V0D2, CEBPA, CEBPB, DBP, FOSL1, FOXA2, FOXP3, GATA2, HNF1A, HNF1B, HNF4A, JUNB, LEP, NR3C1, PAX5, POU1F1, PRDM1, PRKG1, RAG1, RAG2, RBL2, TGFB2, TIAM1, UTRN, VCL*
CALCB	other	6.04×10^{-6}	*ATF6, CALR, CD80, CEBPA, CEBPB, CREM, ELK1, ETS1, FOS, FOSL1, ICAM1, JUN, JUNB, JUND, LEP, MYC, NR3C1, TBP, ZEB1*
PROTOR	group	6.06×10^{-6}	*CEBPB, ELK1, ETS1, FHIT, FOS, FOSL1, FOXM1, FOXP3, GATA2, HES1, JUN, LEP, MYC, PGR, RAG1, UTRN, WT1, ZEB1*
NFATC4	transcription regulator	6.07×10^{-6}	*ABCA4, ACER1, ARHGEF9, ATF3, ATP6V1H, CDC42BPB, CEBPA, CEBPB, CHMP4A, CIT, CLEC7A, CNOT4, EPHA3, EYA4, FMN1, FOS, FOXA1, FOXO4, FOXP3, GGCT, GNAQ, ICAM1, IQGAP1, IRF1, JUN, JUNB, JUND, KCND2, KPNA3, LEP, MAP3K3, MYC, NEK1, NLGN1, NLGN4X, PDCD4, PLAC1, PLCB1, PTGER3, RBL2, ROBO1, SP1, SPAG9, TCF7L2, TGFB2, THRB, TJP2, TPP2*
DDX5	enzyme	6.18×10^{-6}	*ABCA4, AHR, ARHGEF9, ATF3, ATP6V1H, CAST, CDC42BPB, CEBPA, CEBPB, CHD9, CHMP4A, CIT, CLEC7A, CNOT4, ETS2, FMN1, FOS, FOSL1, FOXA1, FOXM1, FOXO4, FOXP3, GATA1, GLCCI1, GNAQ,*

			HNF1A, ICAM1, IQGAP1, IRF1, JUN, JUNB, JUND, KPNA3, LEP, MAP3K3, MYC, NEK1, NLGN1, NLGN4X, NR3C1, PAICS, PDCD4, PGR, PLCB1, PTGER3, RBL2, ROBO1, SP1, SPAG9, TCF7L2, TGFB2, THRB, TJP2, TPP2, WT1
GNG3	enzyme	6.22×10^{-6}	*AHR, CEBPA, CEBPB, CXCL9, DBP, ETS1, FOS, FOXP3, IRF1, JUN, JUNB, LEP, MYC, NR3C1, POU1F1, SELP, STAT4*
WNT11	other	6.41×10^{-6}	*AHR, ATF3, ATF6, BCL11B, DBP, ETS1, FOS, FOSL1, GRIK2, ICAM1, IRF1, JUNB, MYC, PAX5, POU1F1, SP1, TGFB2, WT1*
DGAT1	enzyme	6.64×10^{-6}	*AHR, ATF3, ATF6, BCL11B, CD80, CEBPB, DBP, ELK1, ETS1, FOS, FOSL1, FOXO4, FOXP3, ICAM1, IRF1, JUN, JUNB, JUND, KCND2, LEP, MOG, PFKP, PHLPP1, POU1F1, RBP1, SP1, TBP*
APBB1	transcription regulator	6.65×10^{-6}	*ABCA4, ARHGEF9, ATF3, ATP6V1H, CDC42BPB, CEBPA, CEBPB, CHMP4A, CIT, CLEC7A, CNOT4, FMN1, FOS, FOXM1, FOXO4, FOXP3, GNAQ, HES1, ICAM1, IQGAP1, IRF1, JUN, JUNB, JUND, KPNA3, LEP, MAP3K3, MYC, NEK1, NLGN1, NLGN4X, PDCD4, PGR, PLCB1, PTGER3, RBL2, ROBO1, SP1, SPAG9, TCF7L2, TGFB2, THRB, TJP2, TPP2*
GHSR	G-protein coupled receptor	6.66×10^{-6}	*ATF3, CD80, CEBPB, CREM, ELK1, ETS1, FHIT, FOSL1, FOXM1, GATA2, HES1, ICAM1, ICAM5, JUN, LEP, MYC, PGR, RAG1, UTRN, WT1, ZEB1*
PSMC5	transcription regulator	6.95×10^{-6}	*ABCA4, ACER1, ARHGEF9, ATF3, ATP6V1H, CDC42BPB, CEBPA, CEBPB, CHMP4A, CIT, CLEC7A, CNOT4, EPHA3, EYA4, FMN1, FOS, FOXA1, FOXO4, FOXP3, GGCT, GNAQ, HNF1B, ICAM1, IQGAP1, IRF1, JUN, JUNB, JUND, KPNA3, LEP, MAP3K3, MYC, NEK1, NLGN1, NLGN4X, PDCD4, PLAC1, PLCB1,*

			PTGER3, RBL2, ROBO1, SP1, SPAG9, TCF7L2, TGFB2, THRB, TJP2, TPP2
PDIA3	peptidase	6.98 x 10^{-6}	*AHR, ATF3, ATF6, BCL11B, CEBPA, DBP, FOS, FOSL1, FOXP3, ICAM1, JUN, JUNB, LCP1, LEP, MOG, PDCD4, PFKP, PHLPP1, POU1F1, SELP, SP1*
Selectin	group	6.99 x 10^{-6}	*ABCA4, AFAP1, ALDH4A1, ANKH, ANKS1B, APBB2, ARHGEF9, ATF1, ATF6, ATP11B, ATP6V0D2, ATP6V1H, ATXN1, BCAR3, BRF2, CAMK2D, CDC42BPB, CEBPB, CHI3L2, CHMP4A, CIT, CLEC7A, CLIP1, CLSTN2, CNOT4, CNTNAP2, COL14A1, CSRP2, DAB1, DBP, DHX9, EIF2B1, ELK1, EP400, EPHA3, ETS2, FEM1B, FHIT, FMN1, FOS, FOXA1, FOXJ1, FOXO4, GADD45GIP1, GH1, GLCCI1, GMPR, GNAI2, GNAQ, GRIA4, GRIK2, HDAC9, HIP1, HNF1A, HNF1B, HNF4A, HOXA5, IL1R2, IQGAP1, IRF1, IRF2, IVNS1ABP, JUN, JUNB, KCND2, KCNN2, KIR3DL1, KPNA3, LCP1, LEP, LRP2, LUC7L3, MAP3K3, MDGA1, MIGA1, MIOX, MYC, MYO10, NEDD8, NEK1, NLGN1, NLGN4X, NR3C1, PAFAH1B1, PAICS, PHLPP1, PKIA, PLA2R1, PLCB1, POU1F1, PPAT, PPM1H, PRKD1, PRKN, PTGER3, PTPN11, PTPRS, RAB3C, RAD23A, RAG1, RASGRF1, RGS12, RPL8, SCRIB, SEMA4A, SHROOM3, SMARCD2, SPAG9, ST3GAL5, ST8SIA5, STAT4, STC1, SUCLG1, SV2C, TFAP2A, TFCP2, THRB, TIAM1, TJP2, TMED2, TNFRSF8, TPP2, USP33, UTRN, WNT2, WT1, WWOX, ZFP57, ZNF148*
APLNR	G-protein coupled receptor	7.11 x 10^{-6}	*ATF3, CD80, CEBPB, ELK1, ETS1, FHIT, FOS, FOSL1, FOXM1, GATA2, HES1, ICAM1, JUN, LEP, MYC, PGR, RAG1, UTRN, WT1, ZEB1*

PIK3R5	kinase	7.11 x 10^{-6}	*CEBPA, CEBPB, CXCL9, ELK1, FHIT, FOS, FOSL1, FOXM1, GATA2, HES1, IRF1, JUN, LEP, MYC, PGR, RAG1, SELP, UTRN, WT1, ZEB1*
IL3RA	transmembrane receptor	7.18 x 10^{-6}	*ABCA4, ABL1, AFAP1, AHR, ALDH4A1, APBB2, API5, ARHGEF9, ATF1, ATP6V0D2, ATP6V1H, ATXN1, BCAR3, BRF2, CAMK2D, CD46, CD80, CDC42BPB, CEBPA, CEBPB, CHI3L2, CHMP4A, CIT, CLEC7A, CLSTN2, CNOT4, CNTNAP2, COL14A1, CPNE5, CXCL9, DAB1, DGKI, DHX9, EIF2B1, ELK1, EPHA3, ETS1, ETS2, EXOC4, FAM171A1, FMN1, FOSL1, FOXA1, FOXM1, GABRA1, GATA1, GH1, GLCCI1, GNAI2, GNAQ, GRIA4, HNF1B, HOXA5, ICAM1, ICAM3, ICAM5, IQGAP1, IRF1, IVNS1ABP, JUN, JUNB, KLF15, KPNA3, LEP, LIMD2, LNPEP, LRP2, MAP3K3, MDGA1, MIGA1, MOG, MTMR7, MYC, MYO10, NEDD8, NEK1, NFATC2, NLGN1, NLGN4X, NOD1, PAFAH1B1, PAICS, PAX5, PGR, PKIA, PLCB1, PLCB4, POLD3, POU1F1, PPAT, PRDM1, PRKD1, PRKN, PSMA2, PTGER3, PTPN11, PTPRS, QKI, RAB10, RAG1, RAG2, RASGRF1, RBP1, RGS12, RP2, RPE65, SCRIB, SEMA4A, SHROOM3, SIPA1L3, SMARCD2, SORCS3, SPAG9, ST3GAL5, ST8SIA5, STC1, SUCLG1, SV2C, TFAP2A, THRB, TJP2, TMED2, TPP2, UBA6, UPK1B, USP33, UTRN, VAMP4, WNT2, WT1, WWOX, YY1, ZEB1, ZFP57, ZNF148*
1-phosphatidyl-D-myo-inositol 4,5-bisphosphate	chemical - endogenous mammalian	7.54 x 10^{-6}	*CEBPB, ELK1, ETS1, FHIT, FOS, FOSL1, FOXM1, GATA2, HES1, ICAM1, JUN, LEP, MYC, PGR, RAG1, UTRN, WT1, ZEB1*

MTORC2	complex	7.68 x 10^{-6}	*AHR, ANKH, ATF6, ATP6V0D2, ATP6V1H, BCL11B, DBP, ELK1, ETS1, FHIT, FOSL1, FOXM1, FOXP3, GATA2, GRIK2, HES1, HNF4A, IRF1, JUN, JUNB, JUND, KCND2, KLF15, LEP, MOG, MRPL13, PDCD4, PFKP, PGR, POU1F1, PRKG1, PSMA2, RAG1, RBL2, RPL8, SLC38A3, TCF7L2, TGFB2, UTRN, WT1, ZEB1*
PIP5K1B	kinase	7.69 x 10^{-6}	*ATF3, BCAR3, CD80, CEBPB, ELK1, ETS1, FHIT, FOSL1, FOXM1, FOXP3, GATA2, HNF4A, ICAM3, JUNB, JUND, LEP, PGR, PRDM1, RAG1, RBP1, RXRA, TBP, UTRN, WT1, ZEB1*
E64d	chemical - protease inhibitor	7.78 x 10^{-6}	*AHR, ATF3, ATF6, BCL11B, BMP6, CD46, CD80, CEBPA, CEBPB, CXCL9, DBP, FOSL1, FOXP3, HES1, HNF4A, ICAM1, JUN, JUND, KLF15, LEP, NFATC2, POU1F1, PRDM1, RBP1, RXRA, SORCS3, SP1, TBP*
SLC9A1	ion channel	7.83 x 10^{-6}	*ATF3, CD46, CD80, CEBPA, CEBPB, CXCL9, ETS1, FOS, ICAM1, IRF1, JUN, JUNB, KLF15, LEP, MYC, NFATC2, PRDM1, RBP1, SORCS3, TBP*
CSNK1G2	kinase	7.86 x 10^{-6}	*ABCA4, ARHGEF9, ATF3, ATP6V1H, CDC42BPB, CEBPA, CEBPB, CHMP4A, CIT, CLEC7A, CNOT4, FMN1, FOS, FOXM1, FOXO4, FOXP3, GNAQ, ICAM1, IQGAP1, IRF1, JUN, JUNB, JUND, KPNA3, LEP, MAP3K3, NEK1, NLGN1, NLGN4X, PAX5, PDCD4, PGR, PIK3R1, PLCB1, PTGER3, RBL2, ROBO1, SP1, SPAG9, TCF7L2, TGFB2, THRB, TJP2, TPP2*
CD84	other	7.87 x 10^{-6}	*ABCA4, ABL1, ACTG1, AFAP1, AGA, AHR, ALDH4A1, APBB2, API5, ARHGEF9, ATF3, ATP6V0D2, ATP6V1H, ATP7B, BCAR3, BCL11B, BRF2, CAMK2D, CD276, CD80, CDC42BPB, CEBPB, CHI3L2, CHMP4A, CIT, CNOT4, CREM, CSRP2, CXCL9, DAB1, DBP,*

			DHX9, EIF2B1, ELK1, EPB41L5, EPHA3, ERAP1, ETS1, FMN1, FOSL1, FOXA1, FOXP3, GADD45GIP1, GH1, GNAQ, HDAC9, HIP1, HNF1A, HNF1B, HOXA5, ICAM1, ICAM3, ICAM5, IL1R2, IQGAP1, IVNS1ABP, JUN, JUNB, KLF15, KPNA3, LARGE1, LCP1, LIMD2, LNPEP, LUC7L3, MAGEA11, MAP3K3, MED12L, MSS51, MTMR7, MYO10, NEDD8, NEK1, NKX2-1, NLGN1, NLGN4X, NTRK2, PAICS, PHLPP1, PIK3R1, PLAC1, PLCB1, POLD3, POU1F1, PRDM1, PRKD1, PRKN, PSMA2, PTGER3, PTPN11, PTPRS, RAB3C, RGS12, RP2, RPL8, SDK1, SELP, SEMA3E, SEMA4A, SHROOM3, SNTB1, SORCS3, SPAG9, STAT4, SUCLG1, TIAM1, TICRR, TJP2, TNFRSF8, TPP2, USP33, UTRN, VAMP4, VCL, VTI1A, WNT2, WWOX, YY1, ZNF148
chondroitin sulfate	chemical - endogenous mammalian	8.12×10^{-6}	*ABCA4, AHR, ALDH4A1, APBB2, ARHGEF9, ARID5B, ATF1, ATF3, ATP6V1H, ATXN1, BCAR3, BRF2, CACNB2, CALR, CAMK2D, CAST, CD46, CD80, CDC42BPB, CEBPA, CEBPB, CHI3L2, CHMP4A, CIT, CLSTN2, CNOT4, CNTNAP2, COL14A1, CSRP2, CXCL9, DAB1, DHX9, EIF2B1, EP400, EPHA3, ETS1, ETS2, F2RL3, F5, FMN1, FOXA1, FOXJ1, FOXO4, GABRA1, GADD45GIP1, GATA1, GBX1, GLCCI1, GMPR, GNAI2, GNAQ, HDAC9, HIP1, HNF1A, HNF1B, ICAM1, ICAM3, IQGAP1, IRF2, JUNB, KCNN2, KLF15, KPNA3, LARGE1, LCP1, LEP, LIMD2, LNPEP, LRP2, MAGEA11, MAP3K3, MAPKAP1, MDGA1, MFAP3, MIGA1, MOG, MSS51, MTMR7, MYC, MYO10, NEDD8, NEK1, NFATC2, NLGN1, NLGN4X, NOD1, NSG1, PAFAH1B1, PAICS, PIK3R1, PLCB1, PLCH1, PPAT, PPM1H, PRDM1, PRKD1, PRKN, PSMA2, PTGER3, PTPN11, PTPRS,*

			RAB10, RAB3C, RAD23A, RAG2, RASGRF1, RBP1, RGS12, RPE65, RPL8, SCRIB, SHROOM3, SLC38A3, SMARCD2, SORCS3, SPAG9, ST3GAL5, ST8SIA5, STC1, SUCLG1, SV2C, TBP, TFAP2A, THRB, TIAM1, TJP2, TNFRSF8, UPK1B, USP33, VAMP4, WNT2, YY1
FAIM	other	8.14×10^{-6}	*CAST, CD80, CEBPB, CXCL9, ELK1, ETS1, FHIT, FOS, FOSL1, FOXM1, FOXP3, GATA2, HES1, ICAM1, IRF1, JUN, LEP, MYC, PGR, RAG1, UTRN, WT1, ZEB1*
Selectin	group	8.17×10^{-6}	*CEBPB, DBP, ELK1, FOS, IRF1, JUN, JUNB, LEP, MYC, NR3C1, POU1F1*
C4A/C4B	peptidase	8.21×10^{-6}	*ATF6, CALR, CD46, CD80, CEBPA, CEBPB, ELK1, ETS1, FOS, FOSL1, ICAM1, JUN, JUNB, JUND, LEP, MYC, SELP, TBP, ZEB1*
CCL18	cytokine	8.33×10^{-6}	*ABCA4, ABL1, AFAP1, ALDH4A1, ANKH, ANXA7, APBB2, API5, ARHGEF9, ATP6V0D2, ATP6V1H, BCAR3, BCL11B, BMP6, BRF2, CAMK2D, CDC42BPB, CHI3L2, CHMP4A, CLEC7A, CNOT4, COL14A1, CREM, CSRP2, DAB1, DGKI, EIF2B1, EP400, EYA4, F2RL3, FAM171A1, FHIT, FMN1, FOS, FOXA2, FOXO4, GABRA1, GNAQ, GRIA4, HIP1, HNF1A, HNF4A, ICAM1, ICAM3, ICAM5, IQGAP1, IRF2, IVNS1ABP, JUN, KCND2, KCNIP4, KLF15, KLHL1, KPNA3, LIMD2, LRP2, LUC7L3, MAGEA11, MAP3K3, MARCH3, MOG, MTMR7, MYO10, NEDD8, NEK1, NFIA, NKX2-1, NLGN1, NLGN4X, NTRK2, PAICS, PDCD4, PFKP, PHLPP1, PIK3R1, PLCB1, POLD3, POU1F1, PPAT, PPM1H, PRKD1, PRKG1, PRKN, PTGER3, PTPN11, PTPRS, QKI, RAB3C, RAG1, RAG2,*

			RGS12, RPE65, RPL8, RXRA, SCRIB, SHROOM3, SORCS3, SP1, SPAG9, STAT4, STC1, SUCLG1, TCF7L2, THRB, TNFRSF8, TPP2, UTRN, VCL, WNT2, WWOX, ZFP57, ZNF148
COPS6	other	8.85×10^{-6}	*CEBPB, ELK1, ETS1, FHIT, FOS, FOSL1, FOXM1, GATA2, HES1, ICAM1, JUN, LEP, MYC, PGR, RAG1, UTRN, WT1, ZEB1*
TGX-221	chemical - kinase inhibitor	8.85×10^{-6}	*CEBPB, ELK1, ETS1, FHIT, FOS, FOSL1, FOXM1, GATA2, HES1, JUN, LEP, MYC, PGR, RAG1, SELP, UTRN, WT1, ZEB1*
PPP3CB	phosphatase	8.86×10^{-6}	*AHR, ATF3, ATP6V0D2, BMP6, CAMK2D, CEBPA, CEBPB, CREM, EPHA3, FOS, FOXA2, FOXM1, GATA2, HNF1A, HNF1B, ICAM1, IRF1, JUN, JUNB, LEP, NFATC2, NR3C1, POU1F1, PRDM1, RBL2, TGFB2, UTRN*
WY X 10-[12]5132	chemical - kinase inhibitor	9.16×10^{-6}	*AHR, CEBPA, CEBPB, ELK1, ETS1, FHIT, FOS, FOSL1, FOXM1, GATA2, HES1, IRF1, JUN, KCND2, LEP, MOG, MYC, PFKP, PGR, PHLPP1, RAG1, UTRN, WT1, ZEB1*
VLDL	complex	9.17×10^{-6}	*CD80, CEBPB, ELK1, ETS1, FHIT, FOS, FOSL1, FOXM1, GATA2, HNF4A, ICAM1, JUND, LEP, PGR, RAG1, RBP1, RXRA, TBP, UTRN, WT1, ZEB1*
ZFHX3	transcription regulator	9.21×10^{-6}	*ABCA4, ACER1, ACTG1, AHR, ALDH4A1, APBB2, ARHGEF9, ATF6, ATP6V1H, ATXN1, BCAR3, CAMK2D, CD46, CDC42BPB, CHMP4A, CIT, CLEC7A, CNOT4, COL14A1, CREM, CXCL9, DGKI, EIF2B1, ELK1, EPHA3, ERAP1, ETS1, EYA4, FMN1, FOS, FOXA2, FOXO4, FOXP3, GATA2, GGCT, GNAQ, HDAC9, HNF1B, HNF4A, ICAM1, ICAM3, ICAM5, IL1R2, IQGAP1, KPNA3, LRP2, MAP3K3, NEDD8, NEK1, NFATC2, NLGN1,*

			NLGN4X, NR3C1, PAX5, PDCD4, PFKP, PGR, PLAC1, PLCB1, POU1F1, PRKD1, PRKG1, PRKN, PSMA2, PTGER3, PTPN11, QKI, RAD23A, RAG1, RBP1, RGS12, RPE65, SCRIB, SELP, SEMA3E, SHROOM3, SMARCD2, SP1, SPAG9, ST8SIA5, SUCLG1, TCF7L2, TFAP2A, TGFB2, THRB, TJP2, TNFRSF8, TPP2, UTRN, VCL, WNT2, WT1, ZEB1
Ube3	group	9.32×10^{-6}	*CEBPB, ELK1, ETS1, FHIT, FOS, FOSL1, FOXM1, GATA2, HES1, ICAM1, JUN, LEP, MYC, PGR, RAG1, UTRN, WT1, ZEB1*
beta-estradiol	chemical - endogenous mammalian	9.34×10^{-6}	*ABCA4, ABL1, ACER1, ADM2, AFAP1, AHR, ALDH4A1, ANKS1B, APBB2, API5, ARHGEF9, ARID5B, ATF1, ATF6, ATP6V1H, ATXN1, BCAR3, BCL11B, BRF2, CD276, CDC42BPB, CEBPA, CEBPB, CHI3L2, CHMP4A, CIT, CLEC7A, CNOT4, CNTNAP2, CORO7/CORO7-PAM16, CREM, CSRP2, DGKI, DHX9, EIF2B1, EPHA3, ETS1, EYA4, F5, FAM171A1, FEM1B, FHIT, FMN1, FOS, FOXA1, FOXA2, FOXM1, FOXO4, FOXP3, FRK, GADD45GIP1, GGCT, GH1, GIPC2, GNAQ, GRIK2, HDAC9, HES1, HIP1, HNF1A, HNF1B, ICAM1, ICAM5, IL1R2, IPO4, IQGAP1, IRF1, IVNS1ABP, JUN, JUNB, JUND, KIR3DL1, KLF15, KPNA3, LARGE1, LIMD2, LRP2, LUC7L3, MAP3K3, MDGA1, MIGA1, MOG, MSS51, MTMR7, MYC, MYO10, NEDD8, NEK1, NKX2-1, NLGN1, NLGN4X, NR3C1, NXPH1, PAFAH1B1, PAICS, PDCD4, PFKP, PGR, PIK3R1, PLA2R1, PLAC1, PLCB1, POLD3, POU1F1, PPAT, PRKD1, PRKG1, PRKN, PSMA2, PTGER3, PTPN11, PTPRS, RAB3C, RAD23A, RAG1, RASGRF1, RBL2, RGS12, RP2, RPE65, RPL8, RXRA, SCRIB, SELP, SEMA3E, SEMA4A, SHOC2, SHROOM3, SLC25A15, SORCS3, SPAG9, STAT4, STC1, SUCLG1, SV2C, SYNE1,*

			TCF7L2, TGFB2, TJP2, TNFRSF8, TPP2, USP33, UTRN, VAMP4, WNT2, WWOX, ZEB1, ZNF148, ZNF536
IGFBP5	other	9.4 x 10^{-6}	*ATF3, ATF6, CALR, CD46, CD80, CLEC7A, CXCL9, DBP, ELK1, FHIT, FOS, FOXM1, GATA2, GNAI2, HES1, ICAM1, JUNB, JUND, KLF15, NFATC2, NOD1, NR3C1, PGR, PIK3R1, POU1F1, PRDM1, RAG1, RBP1, SORCS3, TBP, UTRN, WT1*
RAPSN	other	9.49 x 10^{-6}	*CEBPB, DBP, FOS, GH1, JUN, JUNB, LEP, MYC, NR3C1, POU1F1*
ESR1	ligand-dependent nuclear receptor	9.74 x 10^{-6}	*ABCA4, ARHGEF9, ATF3, ATP6V1H, CDC42BPB, CEBPA, CEBPB, CHMP4A, CIT, CLEC7A, CNOT4, FMN1, FOS, FOXM1, FOXO4, FOXP3, GNAQ, ICAM1, IQGAP1, IRF1, JUN, JUNB, JUND, KPNA3, LEP, MAP3K3, MYC, NEK1, NLGN1, NLGN4X, PDCD4, PGR, PLCB1, PTGER3, RBL2, ROBO1, SP1, SPAG9, TCF7L2, TGFB2, THRB, TJP2, TPP2*
KRT18	other	9.91 x 10^{-6}	*ALDH4A1, CD80, CEBPB, ELK1, ETS1, FHIT, FOS, FOSL1, FOXM1, GATA2, HNF4A, ICAM1, JUND, LEP, PGR, RAG1, RBP1, RXRA, TBP, UTRN, WT1, ZEB1*
miR-20 5-5p (and other miRNAs w/seed CCUUCAU)	mature microRNA	9.91 x 10^{-6}	*ATP6V0D2, CEBPA, CEBPB, ELK1, ETS1, FHIT, FOSL1, FOXM1, FOXP3, GATA2, HES1, JUN, JUNB, LEP, PGR, PHLPP1, RAG1, RBL2, TGFB2, UTRN, WT1, ZEB1*
ARNTL	transcription regulator	1.00 x 10^{-5}	*CEBPA, CEBPB, DBP, ELK1, ETS1, FHIT, FOSL1, FOXM1, GATA2, HES1, JUN, LEP, MYC, NKX2-1, PGR, RAG1, UTRN, WT1, ZEB1*

UBE2G2	enzyme	1.03 x 10^{-5}	*AHR, ANKS1B, ATF3, ATF6, ATP6V0D2, ATXN1, BCAR3, CALR, CAST, CD46, CD80, CIT, CREM, CXCL9, DBP, ELK1, EP400, ERAP1, ETS2, FHIT, FOXA1, FOXA2, FOXM1, GABRA1, GATA1, GLCCI1, GNAI2, HDAC9, HIP1, ICAM3, IL1R2, IRF1, JUNB, JUND, KLF15, MYO10, NFATC2, NKX2-1, NR3C1, PAX5, PFKP, PGR, PPM1H, PRDM1, RAB3C, RAG1, RAG2, RBL2, SELP, SORCS3, STAT4, STC1, TBP, TGFB2, UTRN, VCL, YY1*
Neurotrophin	group	1.09 x 10^{-5}	*AHR, ATF6, BCAR3, BCL11B, BMP6, CALR, CREM, CXCL9, DBP, DHX9, EPHA3, ERAP1, FHIT, FOS, FOSL1, FOXM1, FOXP3, GATA2, GNAQ, GRIA4, HDAC9, HES1, HIP1, HNF4A, ICAM3, IRF1, JUNB, JUND, LEP, MYC, NR3C1, NTRK2, PAFAH1B1, PDCD4, PGR, POU1F1, PRDM1, PRKG1, RAB3C, RAG1, RBL2, RBP1, RXRA, SCRIB, SELP, SEMA3E, SP1, STAT4, TBP, TFAP2A, TMED2, UTRN, WT1, ZEB1*
WWP2	enzyme	1.09 x 10^{-5}	*CAST, CD80, CEBPA, CLEC7A, CREM, CXCL9, DAB1, DBP, ELK1, EP400, ERAP1, ETS1, FAM208A, FOXA1, FOXA2, FOXJ1, FOXM1, FOXO4, GATA1, GATA2, GBX1, HDAC9, HES1, HIP1, ICAM1, IL1R2, IRF1, JUNB, JUND, LCP1, MFAP3, PAX5, PDCD4, PIK3R1, PLAC1, PRDM1, RAB3C, RAG1, RAG2, RBL2, REST, ROBO1, SART1, SBF1, STAT4, SUCLG1, TNFRSF8, VAMP4, ZEB1, ZFP57, ZNF148*
FLT3LG	cytokine	1.10 x 10^{-5}	*CD80, CEBPB, FOXP3, GATA1, ICAM1, PAX5, RAG1, RAG2*
PPM1E	phosphatase	1.10 x 10^{-5}	*ATF3, CREM, FOS, FOSL1, FOXP3, HES1, JUN, JUNB*
IL12A	cytokine	1.14 x 10-5	*ATF3, CD46, CD80, CEBPA, CEBPB, CXCL9, ELK1, ETS1, FHIT, FOSL1, FOXM1, GATA2, HES1, ICAM1, ICAM5, IRF1, JUN, JUNB,*

			KLF15, LEP, NFATC2, PGR, PRDM1, RAG1, RBP1, SORCS3, STAT4, TBP, TPP2, UTRN, VAMP4, WT1, ZEB1
SIGMAR1	transmembrane receptor	1.15×10^{-5}	*ATF3, CEBPB, ELK1, ETS1, FHIT, FOSL1, FOXM1, GATA2, HES1, JUN, LEP, MYC, NTRK2, PGR, RAG1, UTRN, WT1, ZEB1*
AR-12	chemical - kinase inhibitor	1.16×10^{-5}	*ANKH, CEBPB, ELK1, ETS1, FHIT, FOSL1, FOXM1, FOXP3, GATA2, GRIA4, HES1, JUN, JUND, KLF15, LEP, NFATC2, PAX5, PDCD4, PGR, RAG1, RAG2, SP1, TGFB2, UTRN, WT1, ZEB1*
acetic acid	chemical - endogenous mammalian	1.20×10^{-5}	*FOS, JUN, JUNB, LEP*
SPI1	transcription regulator	1.25×10^{-5}	*BCL11B, BMP6, CALR, CAMK2D, CEBPA, CEBPB, ELK1, FOS, FOSL1, FOXO4, FOXP3, GATA1, GATA2, GH1, HES1, IL1R2, IRF1, JUN, JUNB, JUND, LEP, MYC, MYO10, PAX5, PKHD1, PRDM1, RAG1, RAG2, RBP1, SELP, STAT4, TBP, TJP2*
RPS6KA4	kinase	1.25×10^{-5}	*ACTG1, AFAP1, AHR, API5, ARHGAP24, ATF1, ATF6, ATP6V0D2, ATP7B, BCAR3, BMP6, BRF2, CALR, CD276, CD46, CD80, CEBPA, CEBPB, CHI3L2, CIT, CLEC7A, CXCL9, DBP, ELK1, EPB41L5, EPHA3, ERAP1, ETS1, FEM1B, FOS, FOSL1, FOXA1, FOXO4, GABRA1, GADD45GIP1, GH1, GLCCI1, GNAI2, GRIK2, HDAC9, HIP1, HNF1B, ICAM1, ICAM5, IL1R2, IRF1, IRF2, JUN, JUNB, KCND2, LARGE1, LCP1, LEP, LIMD2, MAGEA11, MARCH3, MDGA1, MIGA1, MSS51, MTMR7, MYC, NKX2-1, NR3C1, NTRK2, PAFAH1B1, PAX5, PGR, PHLPP1, PIK3R1, PKHD1, PKIA, PRDM1, PSMA2, RAB3C, RAG2, RASGRF1, RBP1, ROBO1, RPL8, RXRA, SELP, SEMA3E, SEMA4A, SLC38A3, SMARCD2, SNTB1,*

			SORCS3, ST3GAL5, ST8SIA5, STAT4, STC1, SV2C, TFAP2A, TIAM1, TICRR, TMED2, TNFRSF8, TPP2, UTRN, VAMP4, WT1, WWOX, YY1, ZNF148
NR5A2	ligand-dependent nuclear receptor	1.25×10^{-5}	*CEBPB, FOS, FOXA2, HNF1A, HNF4A, ICAM1, JUN, JUNB, MYC*

[1]Master regulators are molecules that control multiple genes in heifer conception rate as identified by the Ingenuity Pathway Analysis through direct or indirect relationships with other molecules.
[2]Molecule type of the master regulator as defined by the Ingenuity Pathway Analysis
[3]Significance value with Bonferroni correction ($p < 1.25 \times 10^{-5}$)
[4]List of positional candidate genes from the genome wide association analysis and transcription binding factor sites regulated by each master regulator
